# Supplementary figures and images for: Asymmetrical localization of Nup107-160 subcomplex components within the nuclear pore complex in fission yeast
Source: PLoS Genet. 2019 Jun 6;15(6):e1008061. doi: 10.1371/journal.pgen.1008061 (PMC6553703; doi:10.1371/journal.pgen.1008061)

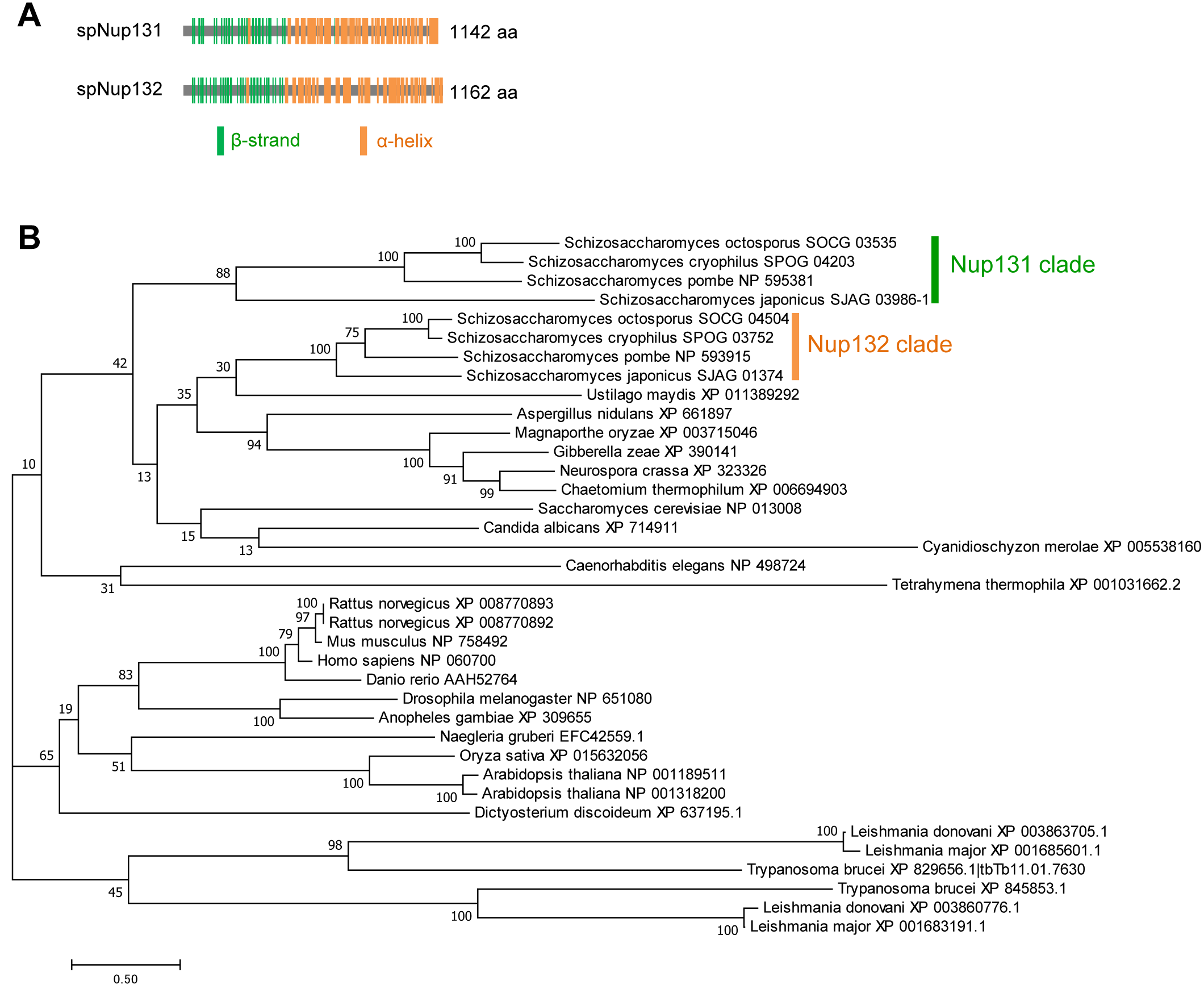

Supplement: S1 Fig — (A) Distribution of secondary structure elements on spNup131 and spNup132. Both spNup131 and spNup132 have structural features in common with Nup133 found in many organisms, such as the N-terminal β-propeller rich region assigned as the Nup133 N-terminal like domain (Pfam PF08801; amino acid residues 44–454 in spNup131 and 50–440 in spNup132) and the C-terminal α-helical stack region assigned as the Non-repetitive/WGA-negative nucleoporin C-terminal domain (Pfam PF03177; a.a. residues 581–1049 in spNup131 and 515–1084 in spNup132). (B) Species names and Genbank accession numbers are shown. spNup131- and spNup132-like proteins found in fission yeasts are indicated. The sequences were aligned using Muscle in MEGA7 software. The evolutionary history was inferred by using the Maximum Likelihood method [78]. The tree with the highest log likelihood (-26923.30) is shown. The bootstrap values are presented next to the branches. Evolutionary analyses were conducted in MEGA7 [79]. (TIF) [file pgen.1008061.s001.tif]

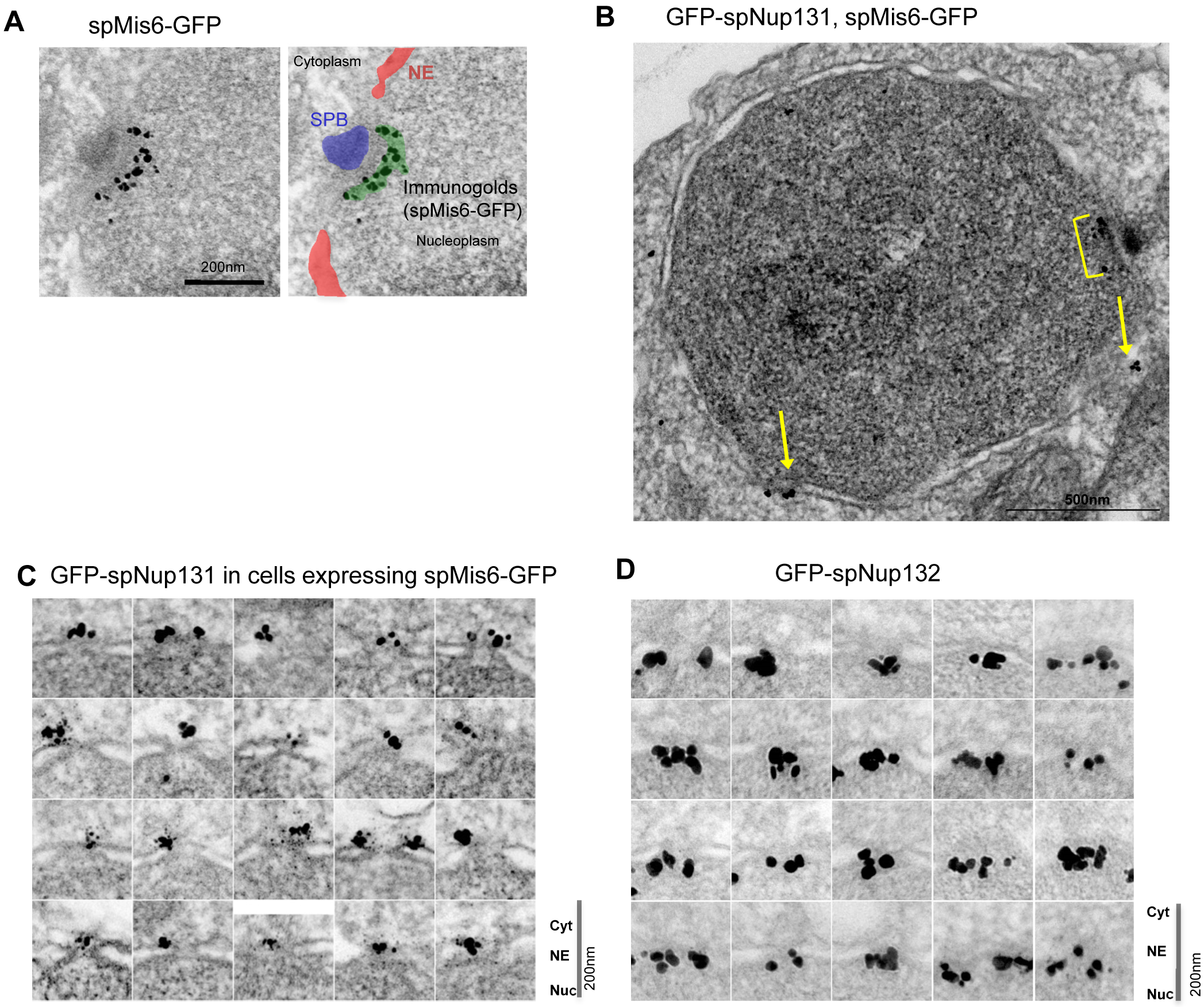

Supplement: S2 Fig — (A) IEM of spMis6-GFP. An original electron micrograph (left) and its duplicated image (right) indicating subcellular structures are shown. SPB, spindle pole body; NE, nuclear envelope. (B) IEM of co-expressed GFP-spNup131 and spMis6-GFP. A representative image is shown. Arrows indicate immunogold at the nuclear pores. The yellow-lined regions indicate immunogold near the SPB, corresponding to the signals from spMis6-GFP. (C, D) Immunoelectron micrographs of 20 nuclear pores used to generate the montage picture and distribution analysis in Fig 1C. Scale bars, 200 nm. (C) IEM of GFP-spNup131 and spMis6-GFP. (D) IEM of GFP-spNup132. (TIF) [file pgen.1008061.s002.tif]

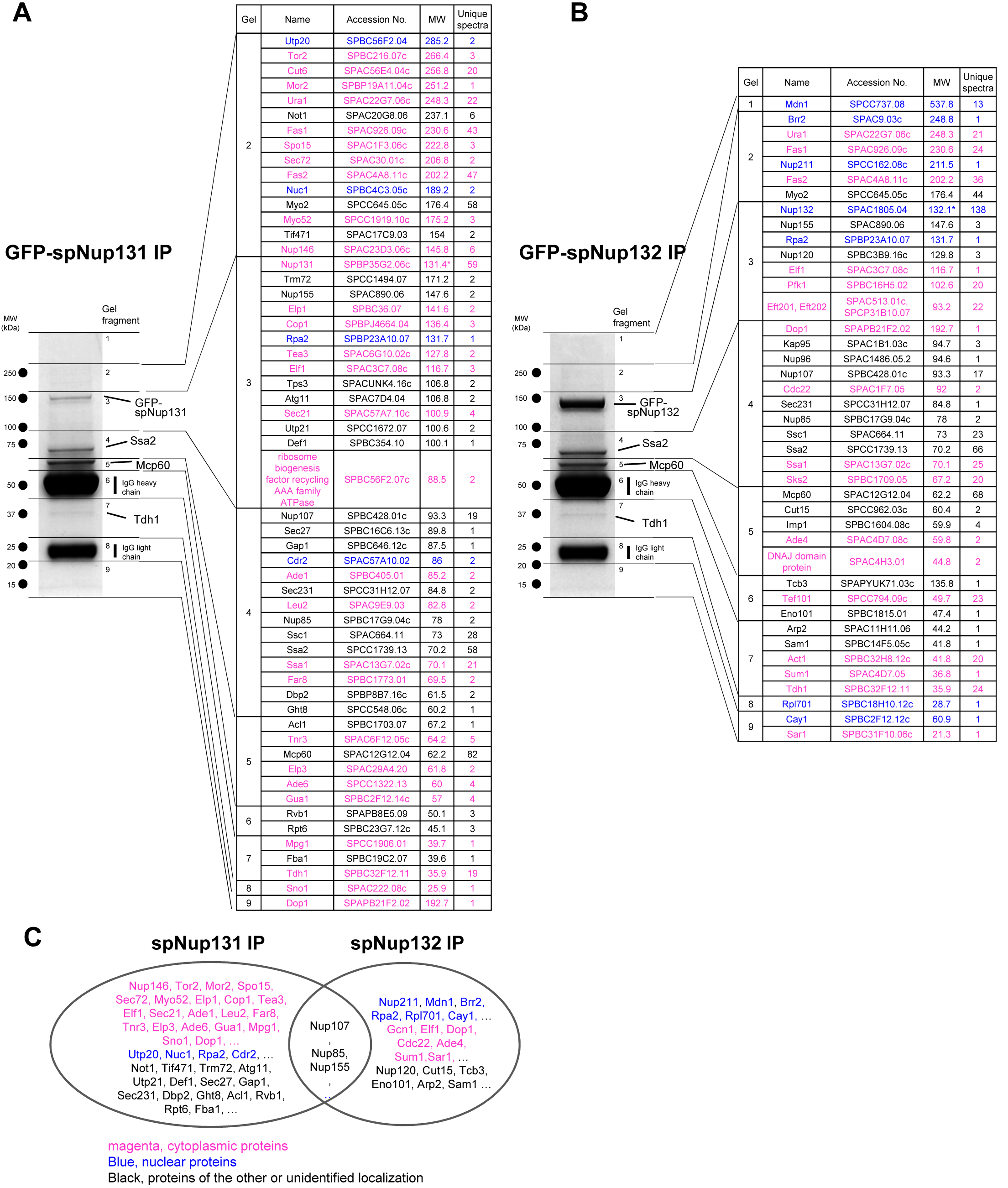

Supplement: S3 Fig — (A, B) Proteins bound to GFP-spNup131 and GFP-spNup132. Images of Coomassie-stained SDS-PAGE gels are shown. Dots indicate the positions of molecular weight marker proteins shown on the left. Each gel was cut at the positions shown by the horizontal lines on the gel image. Proteins that correspond to major bands in each gel fragment were deduced by LC/MS/MS analysis and are indicated on the right. The list on the right shows proteins specifically bound to GFP-spNup131 and GFP-spNup132, Nups, and abundant proteins (>20 spectra). Protein names are colored by their subcellular localizations according to gene ontology data (Pombase: https://www.pombase.org/): magenta, cytoplasmic proteins; blue, nuclear proteins; black, proteins of other or unidentified localizations. (C) Venn diagram showing proteins bound to GFP-spNup131 and GFP-spNup132 identified by LC/MS/MS analysis. Protein names are colored by their subcellular localizations: magenta, cytoplasmic proteins; blue, nuclear proteins; black, proteins of other or unidentified localizations. (TIF) [file pgen.1008061.s003.tif]

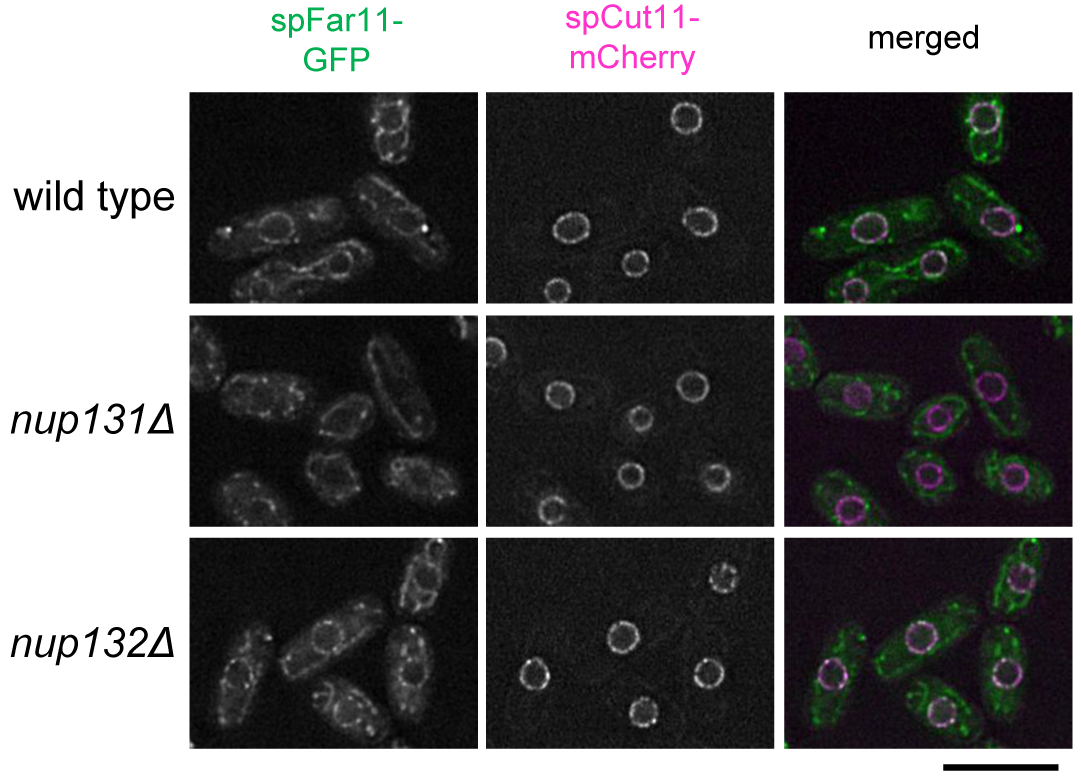

Supplement: S4 Fig — Cells were prepared and observed as described in Fig 2A. Scale bar, 10 μm. (TIF) [file pgen.1008061.s004.tif]

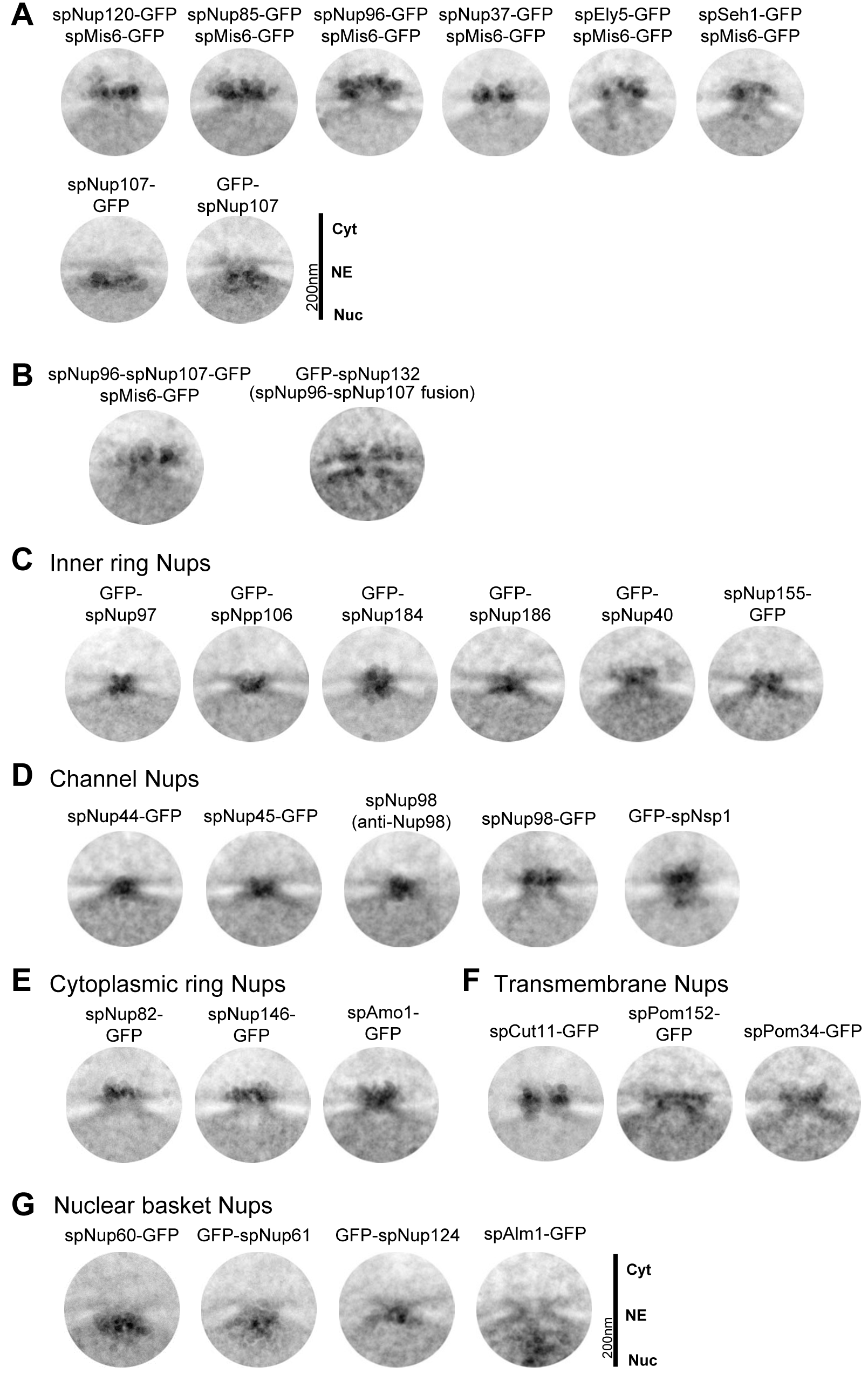

Supplement: S5 Fig — Projection images were made from 20 NPC IEM images for each Nups. (A) Outer ring Nups. Individual IEM images are available in S6 Dataset. (B) spNup96-spNup107-GFP fusion protein and GFP-spNup132 in spNup96-spNup107 fusion strain. Individual IEM images are available in S9 Dataset. (C) Inner ring Nups. (D) Channel Nups. (E) Cytoplasmic ring Nups. (F) Transmembrane Nups. (G) Nuclear basket Nups. Individual IEM images for (C)-(G) are available in S11 Dataset. (TIF) [file pgen.1008061.s005.tif]

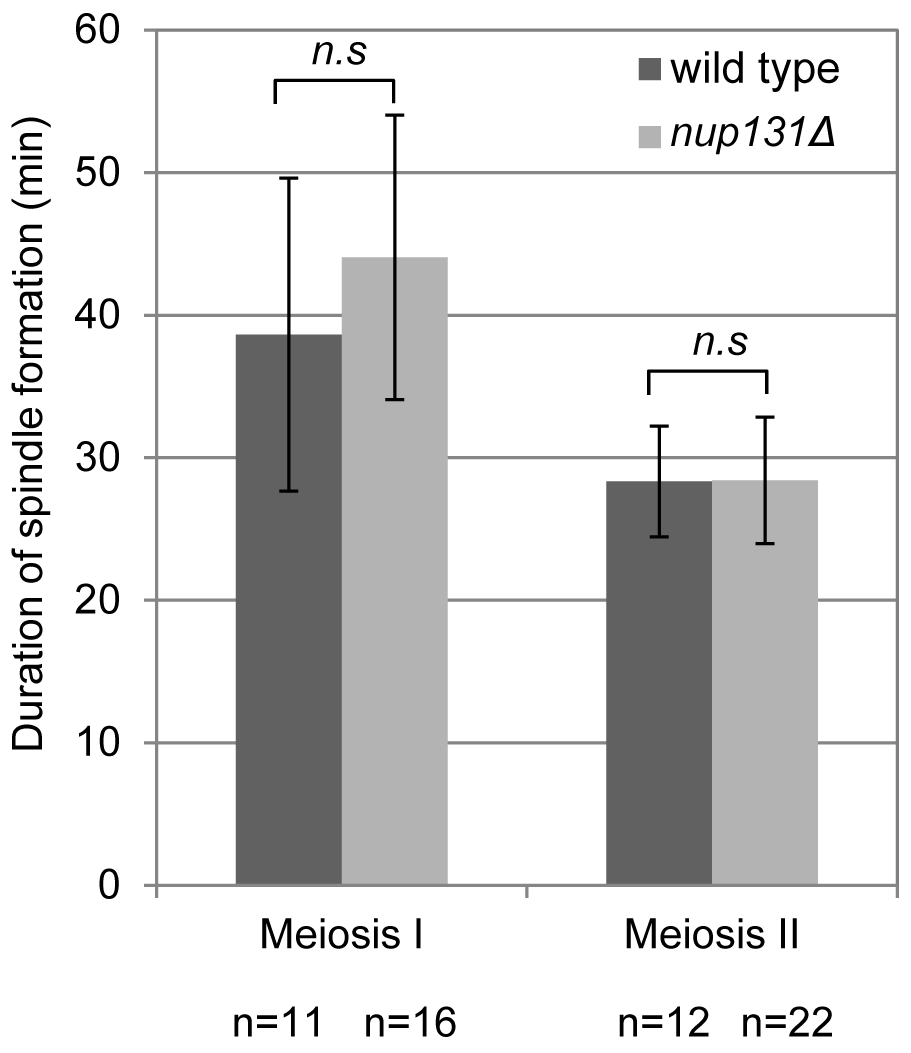

Supplement: S6 Fig — Durations of meiosis I and meiosis II were measured by time-lapse observation. Error bars represent standard deviations. The duration of meiosis I was 38.6 ± 11.0 min in wild type and 44.1 ± 10.0 min in nup131Δ cells (p = 0.41, student’s t-test); the duration of meiosis II was 28.3 ± 3.9 min in wild type and 28.4 ± 4.4 min in nup131Δ cells (p = 0.96, student’s t-test). n.s. stands for no significant difference. Numbers of observed cells are indicated at the bottom. (TIF) [file pgen.1008061.s006.tif]

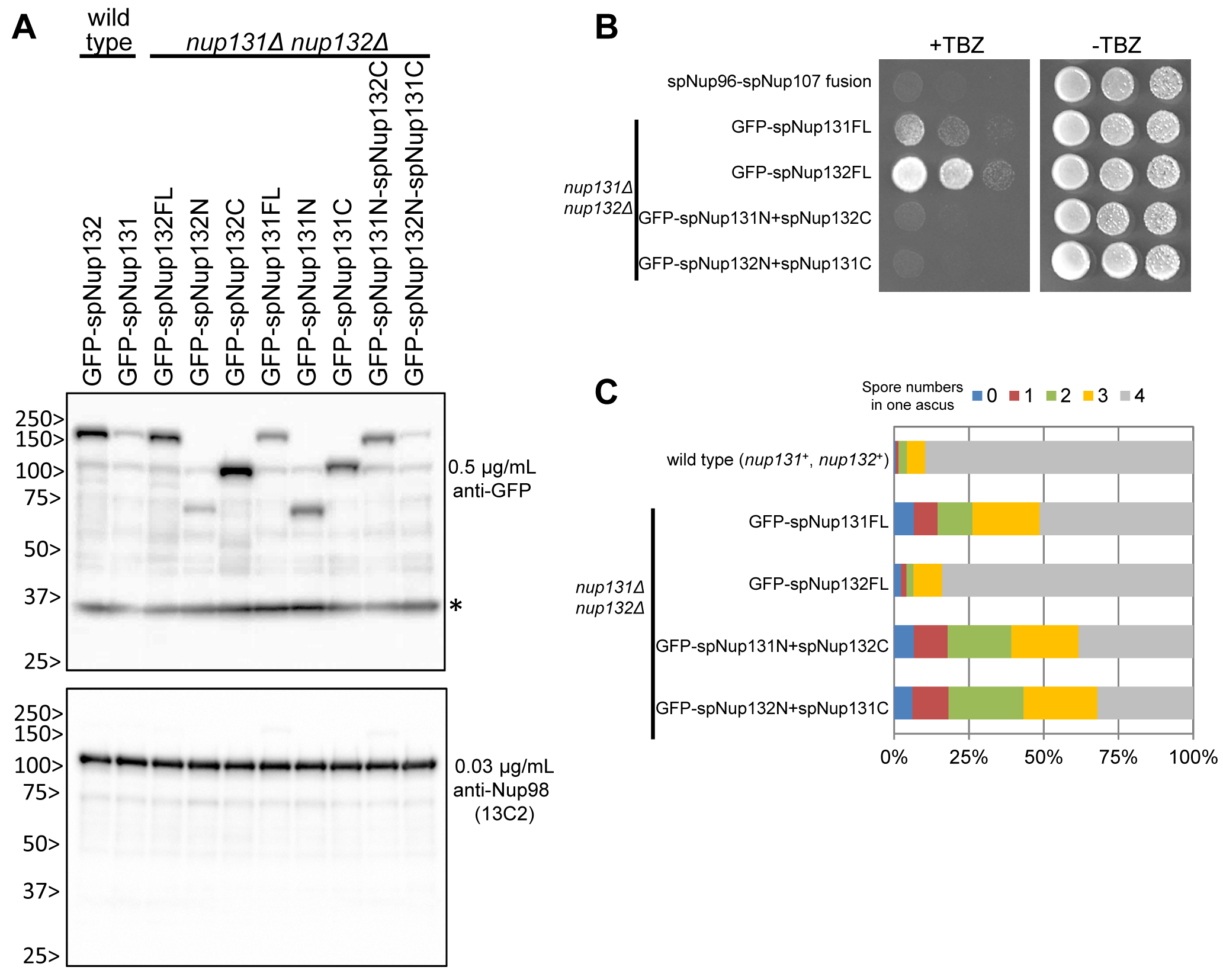

Supplement: S7 Fig — (A) Detection of GFP fused protein fragments by Western blot. nup131Δ nup132Δ cells expressing the indicated protein domains or chimeric proteins were analyzed by Western blot. Extracts prepared from wild type strains expressing GFP-spNup132 and GFP-spNup131 were applied to the left two lanes to examine the expression level of the endogenous proteins. Asterisk indicates a non-specific cross reaction of the anti-GFP antibody. spNup98 was detected with the anti-Nup98 antibody (13C2) as a loading control. (B) TBZ sensitivity of nup131Δ nup132Δ cells expressing the indicated protein domains or chimeric proteins. Five-fold serial dilutions of cells indicated were spotted on YES medium in the presence (+TBZ) or absence (-TBZ) of TBZ (used at 20μg/mL in this experiment). The plates were observed after 3–5 days incubation. (C) Spore formation of nup131Δ nup132Δ cells expressing the indicated proteins. The spore number in each ascus is indicated. More than 200 zygotes were counted for each strain. (TIF) [file pgen.1008061.s007.tif]

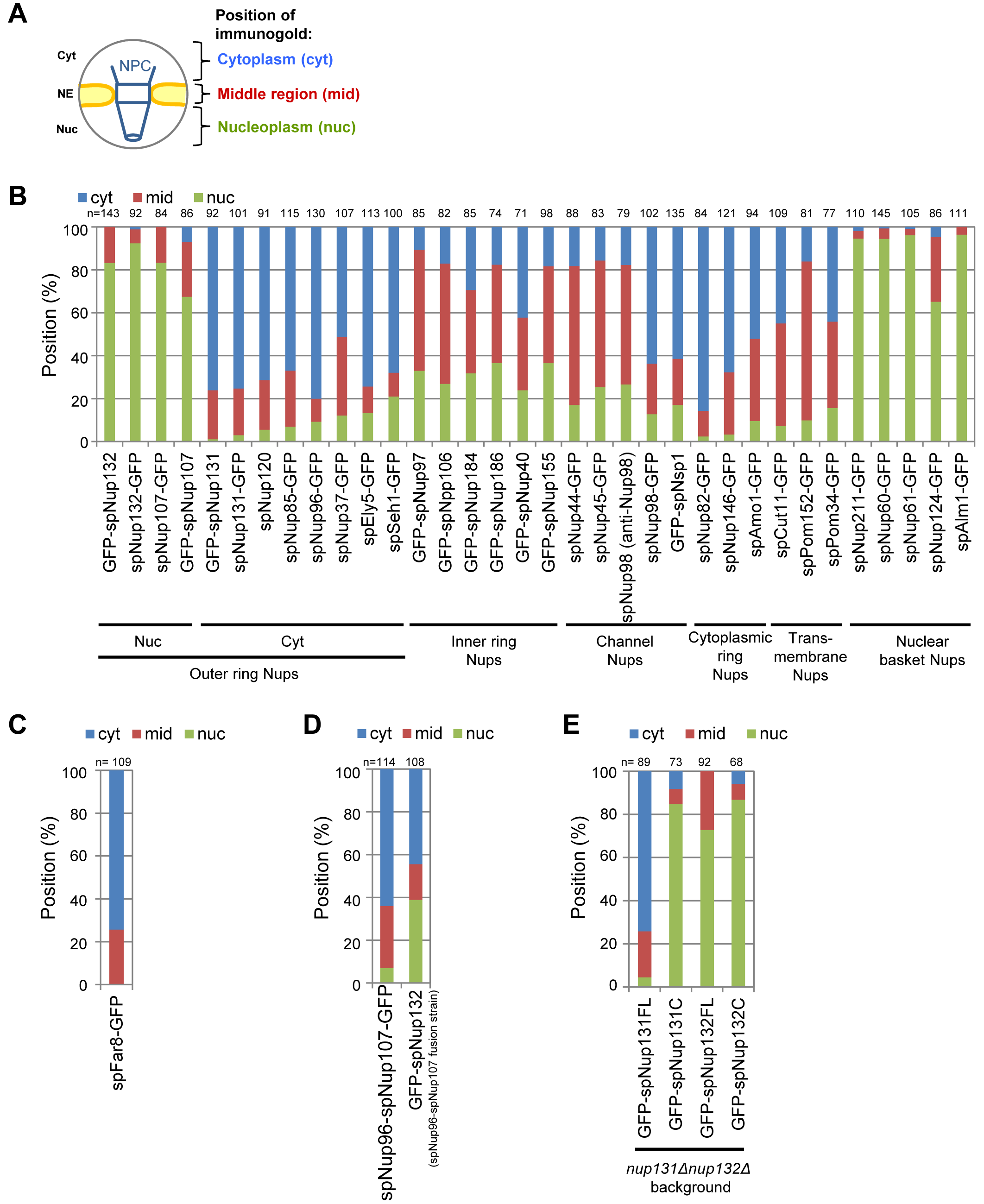

Supplement: S8 Fig — (A) The schematic drawing indicates the cytoplasmic, middle and nuclear regions within the NPC. (B) Distribution of GFP fused proteins indicated at the bottom. The number of immuno-gold particles in the IEM image data (Figs 1B, 1C, 3A, 4A and 7A–7E) was counted for each of the NPC regions. The colored bar graph indicates percentages of each region. The total numbers of gold particles counted are indicated at the top of each column. (C) IEM image data of Fig 2B were analyzed. (D) IEM image data of Fig 5B and 5C were analyzed. (E) IEM image data of Fig 6C were analyzed. (TIF) [file pgen.1008061.s008.tif]
