# Supplementary material for: Asymmetrical localization of Nup107-160 subcomplex components within the nuclear pore complex in fission yeast
Source: PLoS Genet. 2019 Jun 6;15(6):e1008061. doi: 10.1371/journal.pgen.1008061 (PMC6553703; doi:10.1371/journal.pgen.1008061)
Supplement: S1 Table — (DOCX) [file pgen.1008061.s009.docx]

**S1 Table. Nucleoporins in *S. pombe*, *S. cerevisiae*, and *H. sapiens****

| **Subcomplex** | ***S. pombe*** | ***S. cerevisiae*** | ***H. sapiens*** |
| --- | --- | --- | --- |
| Transmembrane Nups | spCut11 | scNdc1 | hsNdc1 |
|  | spPom34/Mug31 | scPom34 | ― |
|  | spPom152 | scPom152 | ― |
|  | spTts1 | scPom33 | hsTMEM33 |
|  |  | scPer33 |  |
|  | ― | ― | hsGp210/Nup210 |
|  | ― | ― | hsPom121 |
| Outer ring Nups | spEly5 | ― | hsELYS |
|  | spNup37 | ― | hsNup37 |
|  | spNup85 | scNup85 | hsNup85 |
|  | spNup107 | scNup84 | hsNup107 |
|  | spNup120 | scNup120 | hsNup160 |
|  | spNup131 | scNup133 | hsNup133 |
|  | spNup132 |  |  |
|  | spNup189c | scNup145c | hsNup96 |
|  | spSeh1 | scSeh1 | hsSeh1 |
|  | ―** | scSec13 | hsSec13 |
|  | ― | ― | hsNup43 |
| Inner ring Nups | spNup97/Mug87 | scNic96 | hsNup93 |
|  | spNpp106 |  |  |
|  | spNup184 | scNup188 | hsNup188 |
|  | spNup186 | scNup192 | hsNup205 |
|  | spNup155 | scNup157 | hsNup155 |
|  |  | scNup170 |  |
|  | spNup40 | scNup53 | hsNup35 (MP-44) |
|  |  | scNup59 |  |
| Channel Nups | spNsp1 | scNsp1 | hsNup62 |
|  | spNup44 | scNup57 | hsNup54 |
|  | spNup45 | scNup49 | hsNup58 |
|  | spNup189n | scNup100 | hsNup98 |
|  |  | scNup116 |  |
|  |  | scNup145n |  |
| Cytoplasmic Nups | spNup82 | scNup82 | hsNup88 |
|  | spNup146 | scNup159 | hsNup214 |
|  | spAmo1 | scNup42/Rip1 | hsNlp1/hCG1/NUPL2 |
|  | ― | ― | hsALADIN |
|  | ― | ― | hsNup358 |
| Nuclear basket Nups | spNup60 | scNup60 | - |
|  | spNup61 | scNup2 | hsNup50 |
|  | spNup124 | scNup1 | hsNup153 |
|  | spAlm1 | ― | ― |
|  | spNup211 | scMlp1 | hsTpr |
|  |  | scMlp2 |  |

*This Table is modified from Asakawa *et al*. 2014.

**Sec13 does not show nuclear periphery localization in *S. pombe*, thus it is not included in the “*S. pombe*” column and is represented as (-) (Asakawa *et al*. 2014).
