# Supplementary material for: Asymmetrical localization of Nup107-160 subcomplex components within the nuclear pore complex in fission yeast
Source: PLoS Genet. 2019 Jun 6;15(6):e1008061. doi: 10.1371/journal.pgen.1008061 (PMC6553703; doi:10.1371/journal.pgen.1008061)
Supplement: S2 Table — (DOCX) [file pgen.1008061.s010.docx]

**S2 Table. *S. pombe* strains used in this study**

| **Figs** | **Description** | **Strain name** | **Genotypes** | **Reference** |
| --- | --- | --- | --- | --- |
| Fig.1, S3Fig | GFP-spNup131 | HA1133 | *h^90^ ade6-216 ura4 leu1-32 lys1^+^::Pnup131-GFP-nup131^+^ nup131::ura4^+^* | Asakawa et al. 2014 |
| Fig.1,  S2D Fig,  S3 Fig,  Fig.5C,  S8B Fig | GFP-spNup132 | HA1131 | *h^90^ ade6-216 ura4 leu1-32 lys1^+^::Pnup132-GFP-nup132^+^ nup132::ura4^+^* | Asakawa et al. 2014 |
| Fig.1,  S2B,C Fig,  S8B Fig. | GFP-spNup131 spMis6-GFP | HA1374-9C | *h^90^ ade6-216 ura4 leu1-32 lys1^+^::Pnup131-GFP-nup131^+^ nup131::ura4^+^ mis6-GFP-LEU2* | This study |
| Fig.1,  S8B Fig. | spNup131-GFP | H03/C12 | *h^90^ ade6-216 ura4 leu1-32 lys1-131 nup131-GFP-HA-kan^r^* | Asakawa et al. 2014 |
| Fig.1,  S8B Fig. | spNup132-GFP | H02/C10 | *h^90^ ade6-216 ura4 leu1-32 lys1-131 nup132-GFP-HA-kan^r^* | Asakawa et al. 2014 |
| Fig.1 | GFP-spNup131 mCherry-spNup132 | HA1619 | *h^90^ ade6-216 ura4-D18 leu1-32 nup131::ura4+ nup132::ura4^+^ lys1^+^::GFP-nup131^+^ aur1^r^::mCherry-nup132^+^* | This study |
| Fig.1 | mCherry-spNup131 GFP-spNup132 | HA1618 | *h^90^ ade6-216 ura4-D18 leu1-32 nup131::ura4^+^ nup132::GFP-nup132^+^ lys1^+^::mCherry-nup131^+^* | This study |
| S2A Fig. | spMis6-GFP | CRLc27 | *h^-^ leu1-32 lys1-131 ade6-216 mis6-GFP::LEU2* | This study |
| Fig.2A,C | wild type spFar8-GFP  spCut11-mCherry | HA1932 | *h^-^ cut11-mCherry-hph far8-GFP-nat* | This study |
| Fig.2A,C | *nup131*Δ  spFar8-GFP  spCut11-mCherry | HA1940 | *h^-^ nup131::kan^r^ cut11-mCherry-hph far8-GFP-nat* | This study |
| Fig.2A,C | *nup132*Δ  spFar8-GFP  spCut11-mCherry | HA1933 | *h^-^ nup132::kan^r^ cut11-mCherry-hph far8-GFP-nat* | This study |
| Fig.2B,  S8C Fig. | spFar8-GFP | HA1824-9B | *h^-^ lys1-131 far8-GFP-nat* | This study |
| Fig.2D,E | Vector | HA2030 | *h^90^ ade6-216 ura4-D18 leu1-32 lys1^+^::pCST3(empty vector) nup131::ura4^+^ cut11-mCherry-hph far8-GFP-kan^r^* | This study |
| Fig.2D,E | spNup131op | HA2031 | *h^90^ ade6-216 ura4-D18 leu1-32 lys1^+^::Pnmt1-nup131^+^ nup131::ura4^+^ cut11-mCherry-hph far8-GFP-kan^r^* | This study |
| Fig.2D,E | spNup132op | HA2032 | *h90 ade6-216 ura4-D18 leu1-32 lys1+::Pnmt1-nup132+ nup131::ura4+ cut11-mCherry-hph far8-GFP-kanr* | This study |
| S4 Fig. | wild type spFar11-GFP  spCut11-mCherry | HA1936 | *h^-^ cut11-mCherry-hph far11-GFP-nat* | This study |
| S4 Fig. | *nup131*Δ  spFar11-GFP  spCut11-mCherry | HA1942 | *h^-^ nup131::kan^r^ cut11-mCherry-hph far11-GFP-nat* | This study |
| S4 Fig. | *nup132*Δ  spFar11-GFP  spCut11-mCherry | HA1937 | *h^-^ nup132::kan^r^ cut11-mCherry-hph far11-GFP-nat* | This study |
| Fig.3A,  S8B Fig. | spNup211-GFP | YN023-2-2A | *h^90^ leu1-32 lys1-131 ura4-D18 ade6-216 nup211-GFP::ura4^+^* | Asakawa et al. 2014 |
| Fig.3B,C | wild type spNup211-GFP spCut11-mCherry | HA1833-1C | *h^-^ ura4 lys1 cut11-mCherry-hph nup211-GFP-ura4^+^* | This study |
| Fig.3B,C | *nup131*Δ  spNup211-GFP  spCut11-mCherry | HA1832-1A | *h^-^ ura4 lys1 nup131::kanr cut11-mCherry-hph nup211-GFP-ura4^+^* | This study |
| Fig.3B,C | *nup132*Δ  spNup211-GFP  spCut11-mCherry | HA1833-1B | *h^-^ ura4 lys1 nup132::nat cut11-mCherry-hph nup211-GFP-ura4^+^* | This study |
| Fig. 3C | *nup132*Δ +vector | HA2040 | *h^-^ ura4 lys1^+^::pCST3 nup132::nat cut11-mCherry-hph nup211-GFP-ura4^+^* | This study |
| Fig. 3C | *nup132*Δ +*nup132*^+^ | HA2042 | *h^-^ ura4 lys1^+^::Pnmt1-nup132^+^(cDNA) nup132::nat cut11-mCherry-hph nup211-GFP-ura4^+^* | This study |
| Fig.4,  S5,S8B Figs. | spNup120-GFP spMis6-GFP | HA1628 | *h^90^ ade6-216 ura4-D18 leu1-32 lys1-131 nup120-GFP-HA-kanr mis6-GFP-hph* | This study |
| Fig.4,  S5,S8B Figs. | spNup85-GFP spMis6-GFP | HA1629 | *h^90^ ade6-216 ura4-D18 leu1-32 lys1-131 nup85-GFP-HA-kanr mis6-GFP-hph* | This study |
| Fig.4,  S5,S8B Figs. | spNup96-GFP spMis6-GFP | HA1626 | *h^90^ ade6-216 ura4-D18 leu1-32 lys1-131 nup189c-GFP-HA-kan^r^ mis6-GFP-hph* | This study |
| Fig.4,  S5,S8B Figs. | spEly5-GFP spMis6-GFP | HA1633 | *h^90^ ade6-216 ura4-D18 leu1-32 lys1-131 ely5-GFP-HA-kanr mis6-GFP-hph* | This study |
| Fig.4,  S5,S8B Figs. | spNu37-GFP spMis6-GFP | HA1627 | *h^90^ ade6-216 ura4-D18 leu1-32 lys1-131 nup37-GFP-kan^r^ mis6-GFP-hph* | This study |
| Fig.4,  S5,S8B Figs. | spSeh1-GFP spMis6-GFP | HA1636 | *h^90^ ade6-216 ura4-D18 leu1-32 lys1-131 seh1-GFP-HA-kan^r^ mis6-GFP-hph* | This study |
| Fig.4,  S5,S8B Figs. | spNup107-GFP | H16/D11 | *h^90^ ade6-216 ura4-D18 leu1-32 lys1-131 nup107-GFP-HA-kan^r^* | Asakawa et al. 2014 |
| Fig.4,  S8B Fig. | GFP-spNup107 | HA1657 | *h^90^ ade6-216 ura4-D18 leu1-32 lys1-131 GFP-nup107-kan^r^* | This study |
| Fig.4 | spNup85-GFP mCherry-spNup131 | HA1965 | *h^90^ ade6-216 ura4-D18 leu1-32 nup131::ura4^+^ lys1^+^::Pnup131-mCherry-nup131 nup85-GFP-kan^r^* | This study |
| Fig.4 | spNup85-GFP mCherry-spNup132 | HA1966 | *h^90^ ade6-216 ura4-D18 leu1-32 lys1-131 nup132::ura4^+^ aur1^r^::Pnup132-mCherry-nup132 nup85-GFP-kan^r^* | This study |
| Fig.4 | spNup107-GFP mCherry-spNup131 | HA2020 | *h^90^ ade6-216 ura4-D18 leu1-32 nup131::ura4^+^ lys1^+^::Pnup131-mCherry-nup131 nup107-GFP-hph* | This study |
| Fig.4 | spNup107-GFP mCherry-spNup132 | HA2021 | *h^90^ ade6-216 ura4-D18 leu1-32 nup132::ura4^+^ lys1^+^::Pnup132-mCherry-nup132 nup96-GFP-hph* | This study |
| Fig.5A,D,G | wild type | AY160-14D | *h^90^ ade6-216 ura4 leu1-32 lys1-131* | Hayashi et al. 2009 |
| Fig.5A | spNup96-spNup107-GFP | HA1774-2B | *h^90^ ade6-216 ura4 leu1-32 lys1-131 nup189::FL5-nup107full-GFP-kanr nup107::nat* | This study |
| Fig.5B,  S5, S8D Figs. | spNup96-spNup107-GFP  spMis6-GFP | HA1905 | *h^90^ ade6-216 ura4 leu1-32 lys1-131 nup189::FL5-nup107full-GFP-kanr nup107::nat mis6-GFP-hph* | This study |
| Fig.5C,  S5, S8D Figs. | spNup96-spNup107 fusion  GFP-spNup132 | HA1783-14C | *h^90^ ade6-216 ura4 leu1 lys1^+^::GFP-nup132 nup132::ura4^+^ nup189^+^::FL5-nup107full-hph nup107::nat* | This study |
| Fig.5D,G | spNup96-spNup107 fusion | HA1776-6A | *h^90^ ade6-216 ura4 leu1 lys1 nup189^+^::FL5-nup107full-hph nup107::nat* | This study |
| Fig.5E,F,  S6 Fig. | wild type  mCherry-spAtb2 | HA1181 | *h^90^ ade6-210 leu1-32 lys1-131 ura4-D18 aur1^r^-nda3pro-mCherry-atb2^+^* | This study |
| Fig.5E,F | spNup96-spNup107 fusion  mCherry-spAtb2 | HJY896 | *h^90^ ade6-216 leu1-32 lys1-131 ura4-D18 nup189^+^::FL5-nup107full-hph nup107::nat aur1^r^::Pnda3-GFP-atb2^+^* | This study |
| S6 Fig. | *nup131*Δ | HA2107 | *h^90^ ade6-210 ura4-D18 leu1-32 lys1-131 nup131::ura4^+^ aur1^r^::Pnda3-mCherry-atb2^+^* | This study |
| Fig.6B,  S7 Fig. | spNup132FL | HJY760 | *h^90^ ade6-216 ura4-D18 leu1-32 lys1-131 nup131::ura4^+^ nup132::ura4^+^ cut11-mCherry-hph lys1^+^::Pnup132-GFPs65t-nup132FL-Tnmt1* | This study |
| Fig.6B,  S7 Fig. | spNup131FL | HJY773 | *h^90^ ade6-216 ura4-D18 leu1-32 lys1-131 nup131::ura4^+^ nup132::ura4^+^ cut11-mCherry-hph*  *lys1^+^-Pnup132-GFPs65t-nup131FL* | This study |
| Fig.6B,  S7 Fig. | spNup132C | HJY769 | *h^90^ ade6-216 ura4-D18 leu1-32 lys1-131 nup131::ura4^+^ nup132::ura4^+^ cut11-mCherry-hph lys1^+^-Pnup132-GFPs65t-nup132C* | This study |
| Fig.6B,  S7 Fig. | spNup131C | HJY756 | *h^90^ ade6-216 ura4-D18 leu1-32 lys1-131 nup131::ura4^+^ nup132::ura4^+^ cut11-mCherry-hph*  *lys1^+^-Pnup132-GFPs65t-nup131C* | This study |
| Fig.6B,  S7 Fig. | spNup132N | HJY855 | *h^90^ ade6-216 ura4-D18 leu1-32 lys1-131 nup131::ura4^+^ nup132::ura4^+^ cut11-mCherry-hph*  *lys1^+^::Pnup132-GFPs65t-nup132N-Tnmt1* | This study |
| Fig.6B,  S7 Fig. | spNup131N | HJY856 | *h^90^ ade6-216 ura4-D18 leu1-32 lys1-131 nup131::ura4^+^ nup132::ura4^+^ cut11-mCherry-hph*  *lys1^+^::Pnup132-GFPs65t-nup131N-Tnmt1* | This study |
| Fig.6B,  S7 Fig. | spNup131N+spNup132C | HJY763 | *h^90^ ade6-216 ura4-D18 leu1-32 lys1-131 nup131::ura4^+^ nup132::ura4^+^ cut11-mCherry-hph lys1^+^-Pnup132-GFPs65t-nup131N-nup132C* | This study |
| Fig.6B,  S7 Fig. | spNup132N+spNup131C | HJY771 | *h^90^ ade6-216 ura4-D18 leu1-32 lys1-131 nup131::ura4^+^ nup132::ura4^+^ cut11-mCherry-hph lys1^+^-Pnup132-GFPs65t-nup132N-nup131C* | This study |
| Fig.6C,  S8E Fig. | GFP-spNup132FL | HJY728 | *h^90^ ade6-216 ura4-D18 leu1-32 lys1-131 nup131::ura4^+^ nup132::ura4^+^ lys1^+^::Pnup132-GFPs65t-nup132FL-Tnmt1* | This study |
| Fig.6C,  S8E Fig. | GFP-spNup132C | HJY782 | *h^90^ ade6-216 ura4-D18 leu1-32 lys1-131 nup131::ura4^+^ nup132::ura4^+^ lys1^+^::Pnup132-GFPs65t-nup132L+C-Tnmt1* | This study |
| Fig.6C,  S8E Fig. | GFP-spNup131FL | HJY810 | *h^90^ ade6-216 ura4-D18 leu1-32 lys1-131 nup131::ura4^+^ nup132::ura4^+^ lys1^+^-Pnup132-GFPs65t-nup131FL* | This study |
| Fig.6C,  S8E Fig. | GFP-spNup131C | HJY743 | *h^90^ ade6-216 ura4-D18 leu1-32 lys1-131 nup131::ura4^+^ nup132::ura4^+^ lys1^+^::Pnup132-GFPs65t-nup131L+C-Tnmt1* | This study |
| S7 Fig. | GFP-spNup132 | HA1523 | *h^90^ ade6-216 leu1-32 ura4-D18 lys1^+^::Pnup132-GFP::nup132^+^ nup132::ura4^+^ cut11-mCherry-hph* | This study |
| S7 Fig. | GFP-spNup131 | HA1524 | *h^90^ ade6-216 leu1-32 ura4-D18 lys1^+^::Pnup131-GFP::nup131^+^ nup131::ura4^+^ cut11-mCherry-hph* | This study |
| Fig.7,  S5, S8 Figs | GFP-spNup97 | HA1330-1C | *h^90^ ade6-216 ura4-D18 leu1-32 nup97::LEU2 lys1^+^::GFP-nup97* | Asakawa et al. 2014 |
| Fig.7,  S5, S8 Figs | GFP-spNpp106 | HA1658 | *h^90^ ade6-216 ura4-D18 leu1-32 lys1-131 GFP-npp106-kanr* | This study |
| Fig.7,  S5, S8 Figs | GFP-spNup184 | HA1656 | *h^90^ ade6-216 ura4-D18 leu1-32 lys1-131 GFP-nup184-kan^r^* | This study |
| Fig.7,  S5, S8 Figs | GFP-spNup186 | HA1659 | *h^90^ ade6-216 ura4-D18 leu1-32 lys1-131 GFP-nup186-kan^r^* | This study |
| Fig.7,  S5, S8 Figs | GFP-spNup40 | HA1134 | *h^90^ ade6-216 ura4-D18 leu1-32 lys1^+^::Pnup40-GFP-nup40^+^ nup40::ura4^+^* | Asakawa et al. 2014 |
| Fig.7,  S5, S8 Figs | spNup155-GFP | AHP001 | *h^90^ ade6-216 ura4-D18 leu1-32 lys1-131 nup155-GFP-kan^r^* | Asakawa et al. 2014 |
| Fig.7,  S5, S8 Figs | spNup44-GFP | H04/D06 | *h^90^ ade6-216 ura4-D18 leu1-32 lys1-131 nup44-GFP-HA-kan^r^* | Asakawa et al. 2014 |
| Fig.7,  S5, S8 Figs | spNup45-GFP | HA1622 | *h^90^ ade6-216 ura4 leu1-32 lys1-131 nup45-GFP-kan^r^* | Asakawa et al. 2014 |
| Fig.7,  S5, S8 Figs | spNup98-GFP | HA800-7A | *h^90^ ade6-216 ura4-D18 leu1-32 lys1-131 nup189N-GFP-kan^r^* | Asakawa et al. 2014 |
| Fig.7,  S5, S8 Figs | spNup98 (anti-Nup98) | AY160-14D | *h^90^ ade6-216 ura4 leu1-32 lys1-131* | Hayashi et al. 2009 |
| Fig.7,  S5, S8 Figs | GFP-spNsp1 | AHP018 | *h^90^ GFP-nsp1-kan^r^ ade6-216 ura4-D18 leu1-32 lys1-131* | Asakawa et al. 2014 |
| Fig.7,  S5, S8 Figs | spNup82-GFP | H04/H10 | *h^90^ nup82-GFP-HA-kan^r^ ade6-216 ura4-D18 leu1-32 lys1-131* | Asakawa et al. 2014 |
| Fig.7,  S5, S8 Figs | spNup146-GFP | H03/D05 | *h^90^ ade6-216 ura4-D18 leu1-32 lys1-131 nup146-GFP-HA-kan^r^* | Asakawa et al. 2014 |
| Fig.7,  S5, S8 Figs | spAmo1-GFP | HA1062-4C | *h^90^ ade6-216 ura4-D18 leu1-32 lys1-131 amo1-GFP-kan^r^* | Asakawa et al. 2014 |
| Fig.7,  S5, S8 Figs | spCut11-GFP | H14/C04 | *h^90^ ade6-216 ura4-D18 leu1-32 lys1-131 cut11-GFP-HA-kan^r^* | Asakawa et al. 2014 |
| Fig.7,  S5, S8 Figs | spPom152-GFP | H04/D07 | *h^90^ pom152-GFP-HA-kan^r^ ade6-216 ura4-D18 leu1-32 lys1-131* | Asakawa et al. 2014 |
| Fig.7,  S5, S8 Figs | spPom34-GFP | AHP016 | *h^90^ ade6-216 ura4-D18 leu1-32 lys1-131 pom34-GFP-kan^r^* | Asakawa et al. 2014 |
| Fig.7,  S5, S8 Figs | spNup60-GFP | AHP011 | *h^90^ nup60-GFP-kan^r^ ade6-216 ura4-D18 leu1-32 lys1-131* | Asakawa et al. 2014 |
| Fig.7,  S5, S8 Figs | GFP-spNup61 | HA1132 | *h^90^ ade6-216 ura4-D18 leu1-32 lys1^+^::Pnup61-GFP::nup61^+^ nup61::ura4^+^* | Asakawa et al. 2014 |
| Fig.7,  S5, S8 Figs | GFP-spNup124 | HA1135 | *h^90^ ade6-216 ura4-D18 leu1-32 lys1^+^::Pnup124-GFP::nup124^+^ nup124::ura4^+^* | Asakawa et al. 2014 |

,
