# Supplementary material for: Asymmetrical localization of Nup107-160 subcomplex components within the nuclear pore complex in fission yeast
Source: PLoS Genet. 2019 Jun 6;15(6):e1008061. doi: 10.1371/journal.pgen.1008061 (PMC6553703; doi:10.1371/journal.pgen.1008061)
Supplement: S3 Table — (DOCX) [file pgen.1008061.s011.docx]

**S3 Table. Dilution ratios of primary and secondary antibodies used for IEM**

| Figure | Strain | Primary antibody dilution* | Secondary antibody dilution* |
| --- | --- | --- | --- |
| Fig 1 | GFP-spNup131  GFP-spNup131 spMis6-GFP  spNup131-GFP  spNup132-GFP | 1:400 | 1:400 |
|  | GFP-spNup132 | 1:400 | 1:100 |
| Fig 2B | spFar8-GFP | 1:400 | 1:400 |
| Fig 3A | spNup211-GFP | 1:400 | 1:400 |
| Fig 4A | spNup120-GFP spMis6-GFP  spNup85-GFP spMis6-GFP  spNup37-GFP spMis6-GFP | 1:400 | 1:200 |
|  | spNup96-GFP spMis6-GFP  spEly5-GFP spMis6-GFP  spSeh1-GFP spMis6-GFP | 1:1000 | 1:800 |
|  | spNup107-GFP  GFP-spNup107 | 1:400 | 1:400 |
| Fig 5B | spNup96-spNup107-GFP spMis6-GFP | 1:400 | 1:400 |
| Fig 5C | GFP-spNup132 | 1:400 | 1:400 |
| Fig 6C | GFP-spNup131FL  GFP-spNup131C  GFP-spNup132FL | 1:400 | 1:400 |
|  | GFP-spNup132C | 1:400 | 1:1000 |
| Fig 7A | GFP-spNup97 | 1:400 | 1:200 |
|  | GFP-spNup184 | 1:400 | 1:800 |
|  | GFP-spNpp106  GFPspNup186  GFP-spNup40  spNup155-GFP | 1:400 | 1:400 |
| Fig 7B | spNup44-GFP  spNup45-GFP  spNup98-GFP  GFP-spNsp1 | 1:400 | 1:400 |
| Fig 7C | spNup82-GFP  spNup146-GFP  spAmo1-GFP | 1:400 | 1:400 |
| Fig 7D | spCut11-GFP | 1:400 | 1:200 |
|  | spPom152-GFP  spPom34-GFP | 1:400 | 1:400 |
| Fig 7E | spNup60-GFP  spNup61-GFP | 1:400 | 1:200 |
|  | GFP-spNup124  spAlm1-GFP | 1:400 | 1:400 |

*A rabbit polyclonal anti-GFP antibody (600-401-215, Rockland Immunochemicals, Limerick, PA, USA) was used as a primary antibody; a goat anti-rabbit Alexa 594 FluoroNanogold Fab’ fragment (7304, Nanoprobes Inc., Yaphank, NY, USA) was used as a secondary antibody.
