# Supplementary material for: Asymmetrical localization of Nup107-160 subcomplex components within the nuclear pore complex in fission yeast
Source: PLoS Genet. 2019 Jun 6;15(6):e1008061. doi: 10.1371/journal.pgen.1008061 (PMC6553703; doi:10.1371/journal.pgen.1008061)

S4 Dataset

Individual IEM images of 20 NPCs used for superimposed images of Figure 3a (spNup211-GFP)

spNup211-GFP

projection

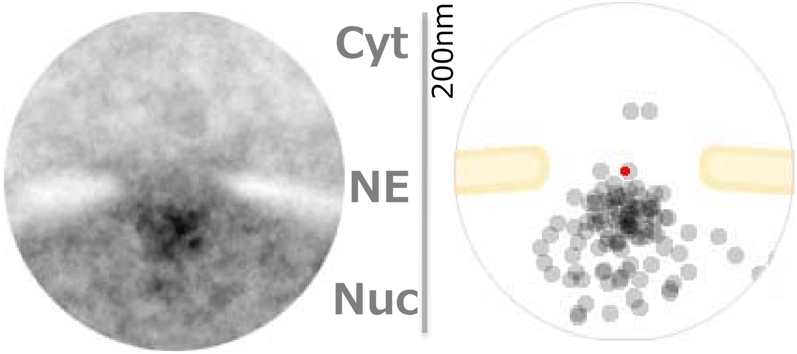

20 NPCs

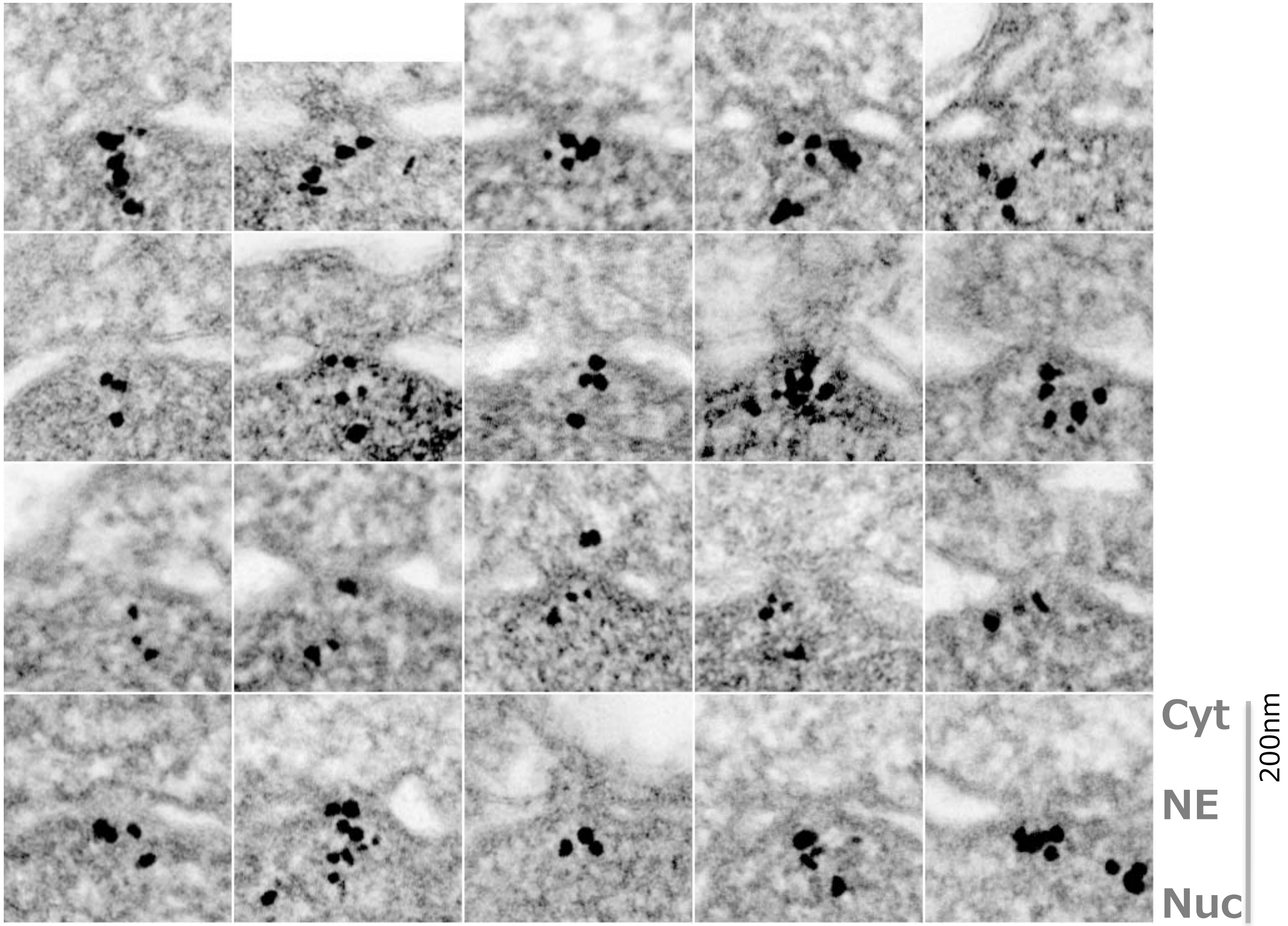

Supplement: S4 Dataset — (PDF) [file pgen.1008061.s015.pdf]
