# Supplementary material for: Asymmetrical localization of Nup107-160 subcomplex components within the nuclear pore complex in fission yeast
Source: PLoS Genet. 2019 Jun 6;15(6):e1008061. doi: 10.1371/journal.pgen.1008061 (PMC6553703; doi:10.1371/journal.pgen.1008061)

S6 Dataset

Individual IEM images of 20 NPCs used for superimposed images of Figure 4a (pNup120-GFP, spNup85-GFP, spNup96-GFP, spNup37-GFP, spEly5-GFP, spSeh1-GFP, spNup107-GFP and GFP-spNup107)

Nup120-GFP

projection

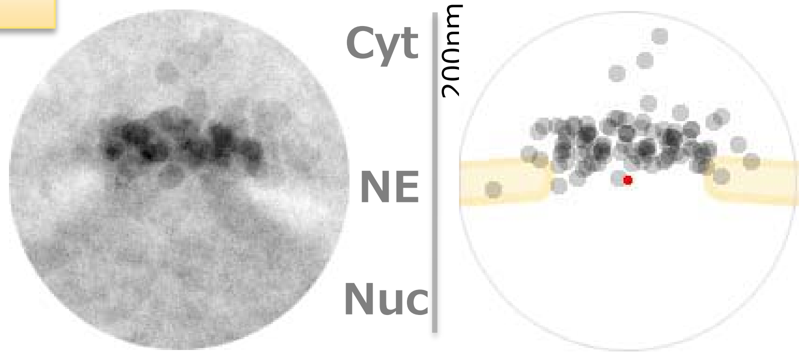

20 NPCs

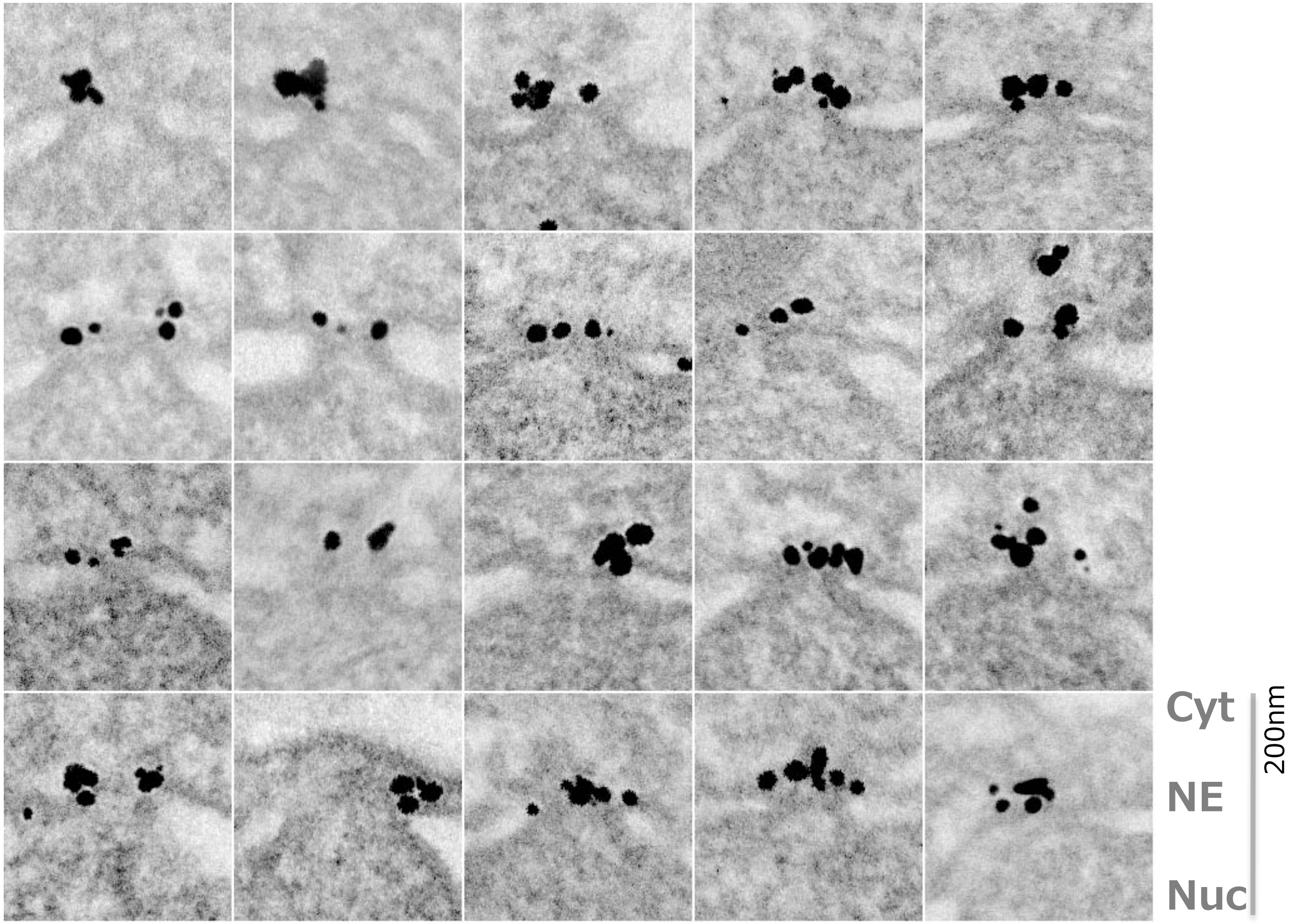

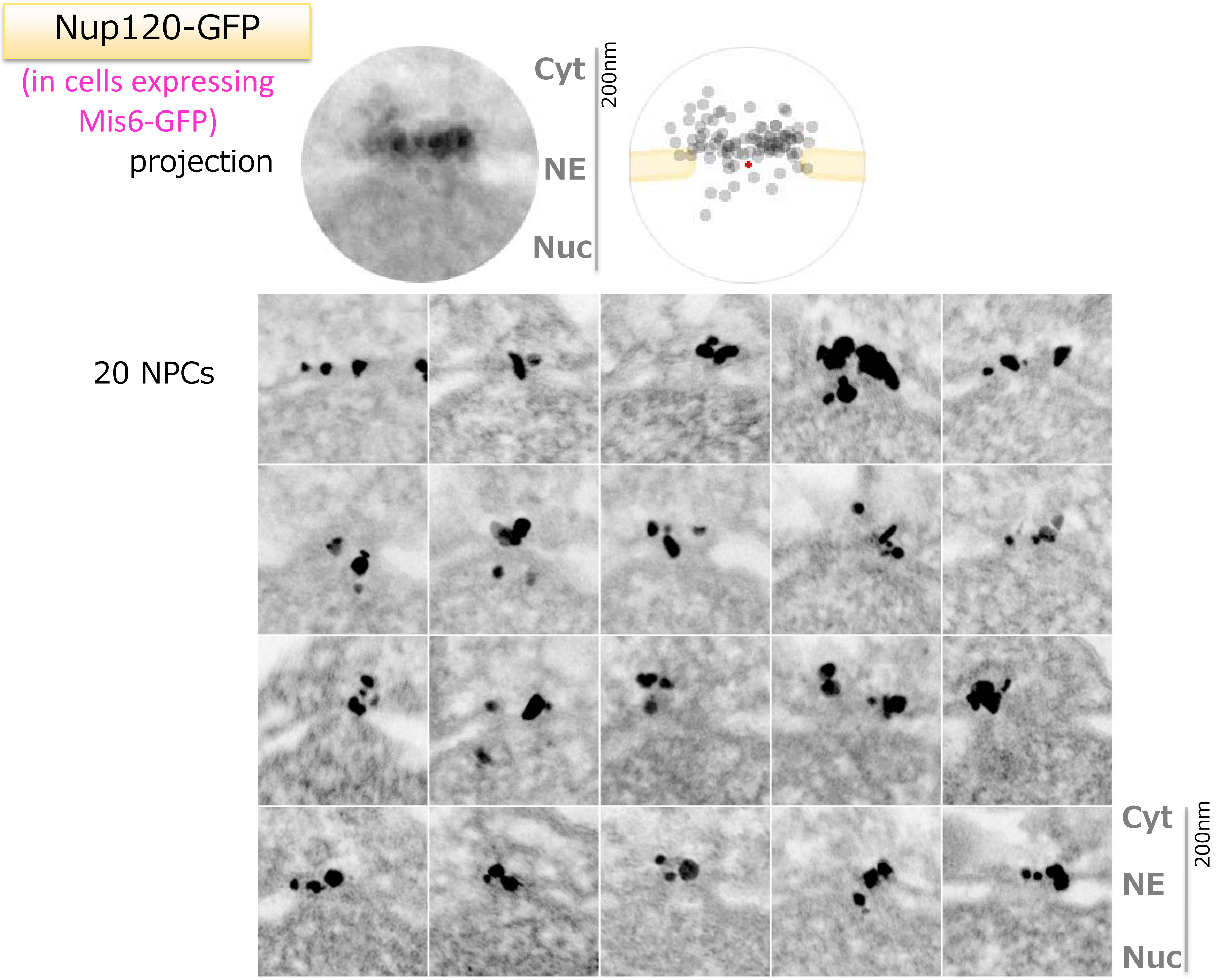
Nup120-GFP
(in cells expressing
Mis6-GFP)
projection
Cyt
NE
Nuc
200nm
20 NPCs
Cyt
NE
Nuc
200nm

Nup85-GFP

projection

20 NPCs

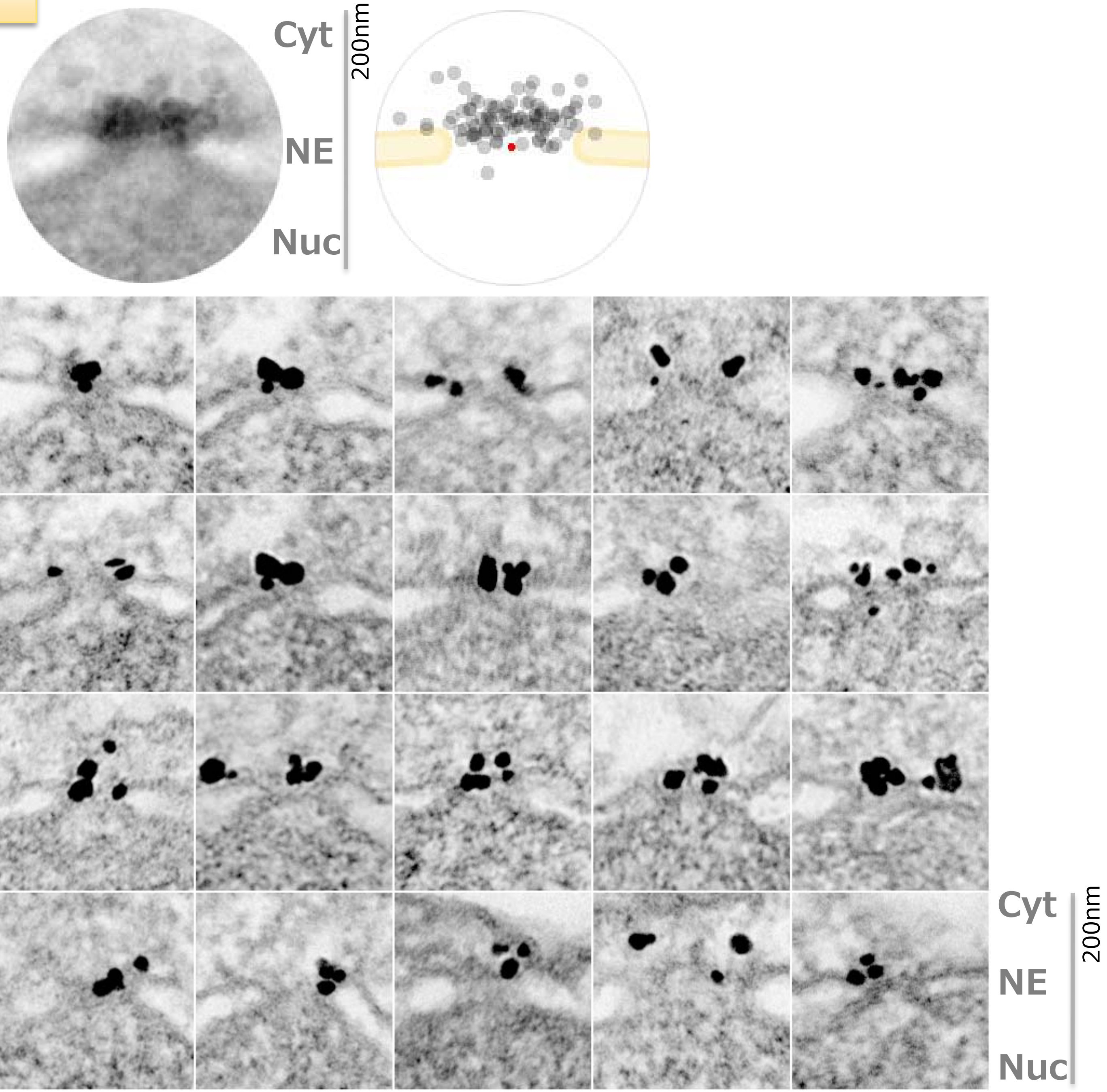

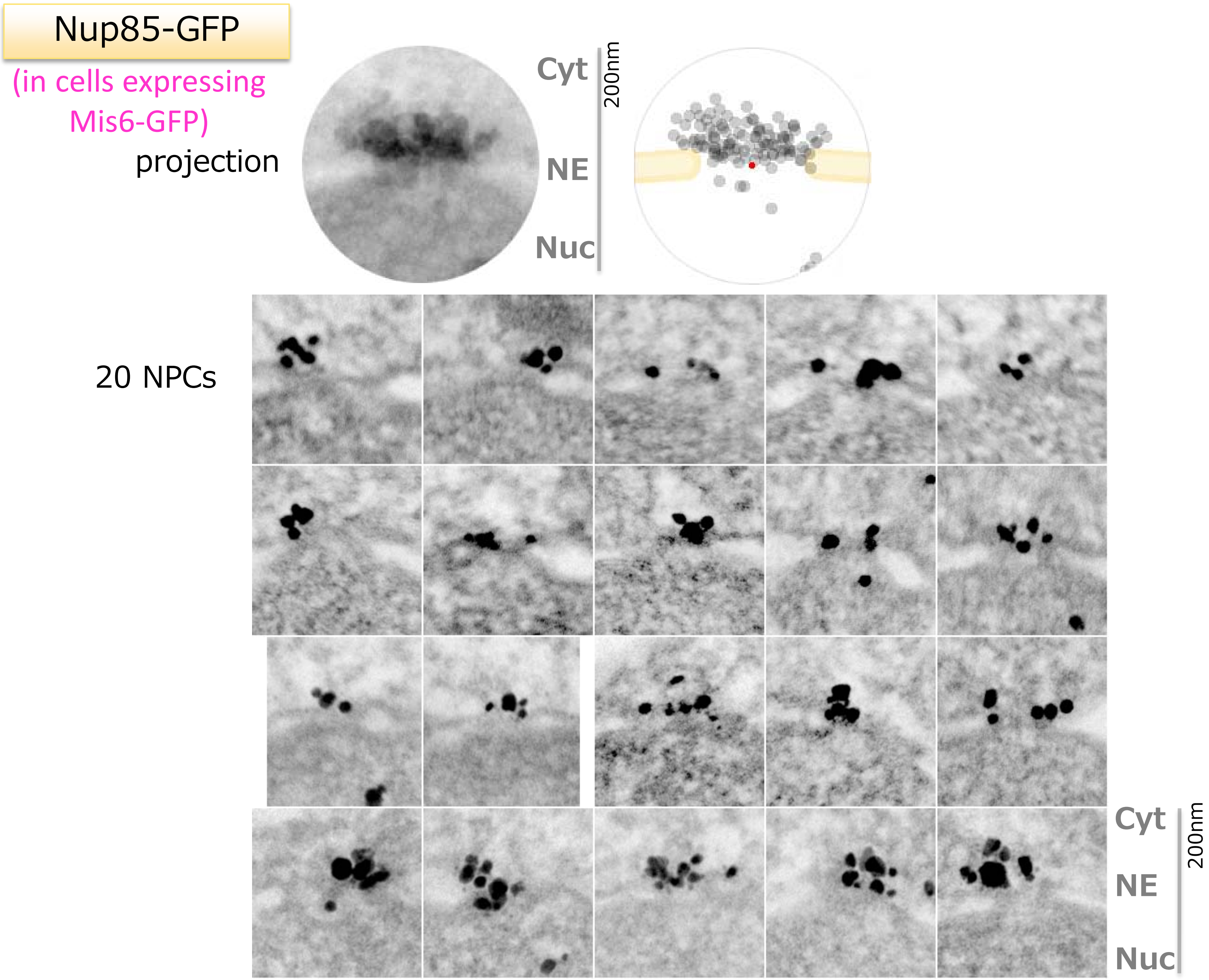
Nup85-GFP
(in cells expressing
Mis6-GFP)
projection
Cyt
200nm
NE
Nuc
20 NPCs
Cyt
200nm
NE
Nuc

Nup96-GFP

projection

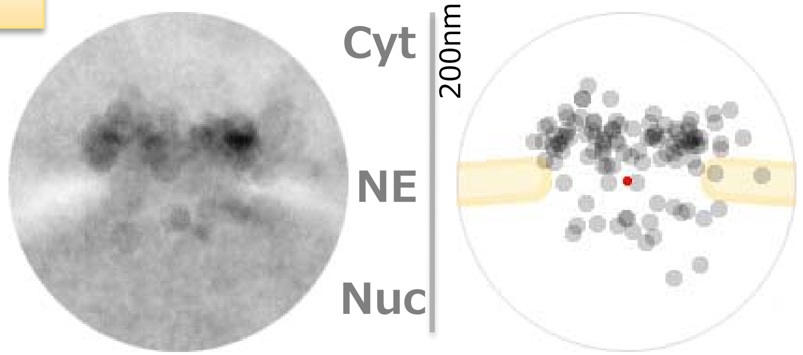

20 NPCs

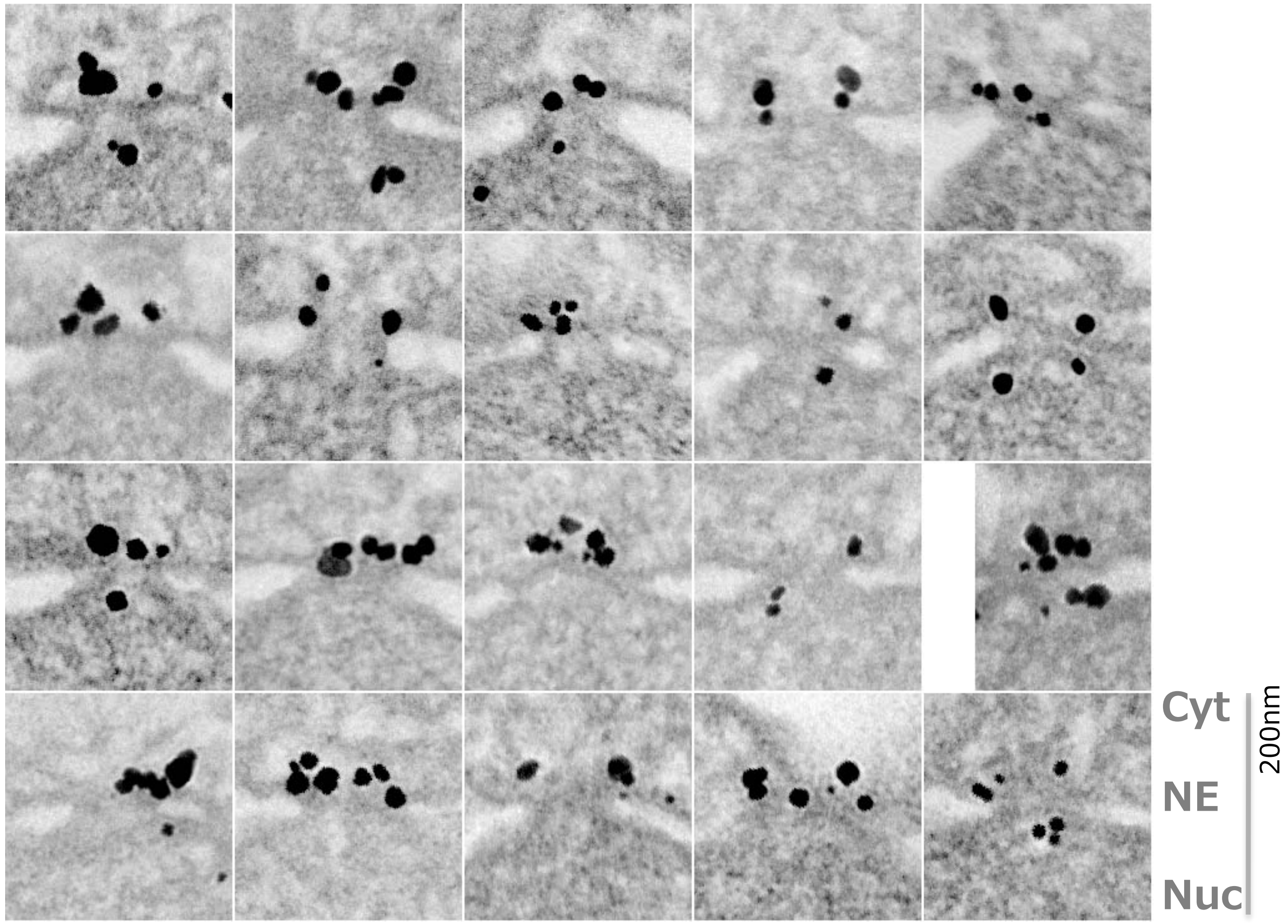

Nup96-GFP

(in cells expressing Mis6-GFP)
projection

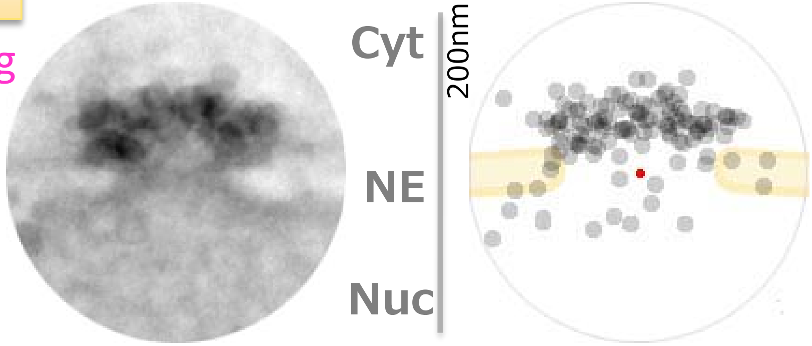

20 NPCs

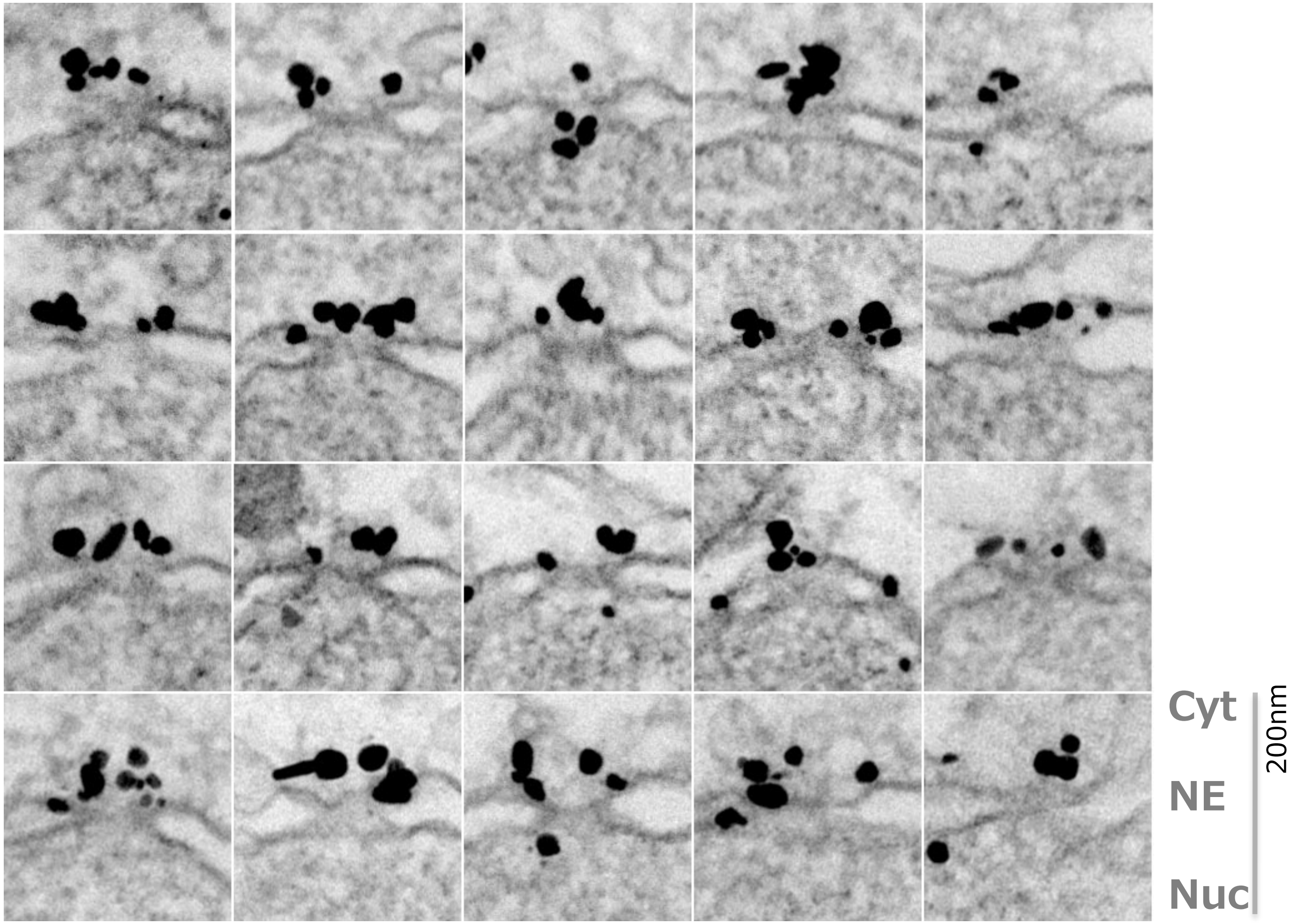

Nup37-GFP

projection

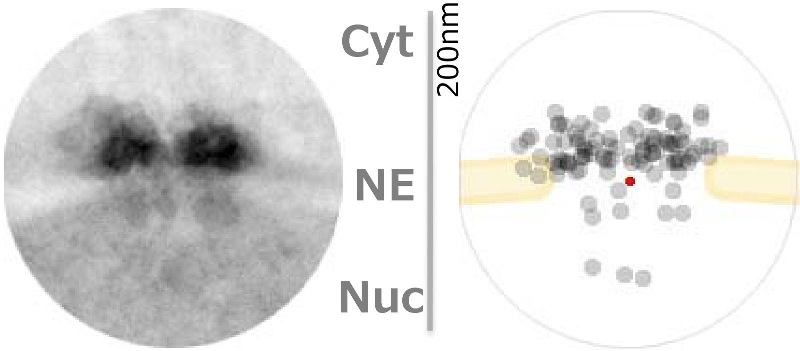

20 NPCs

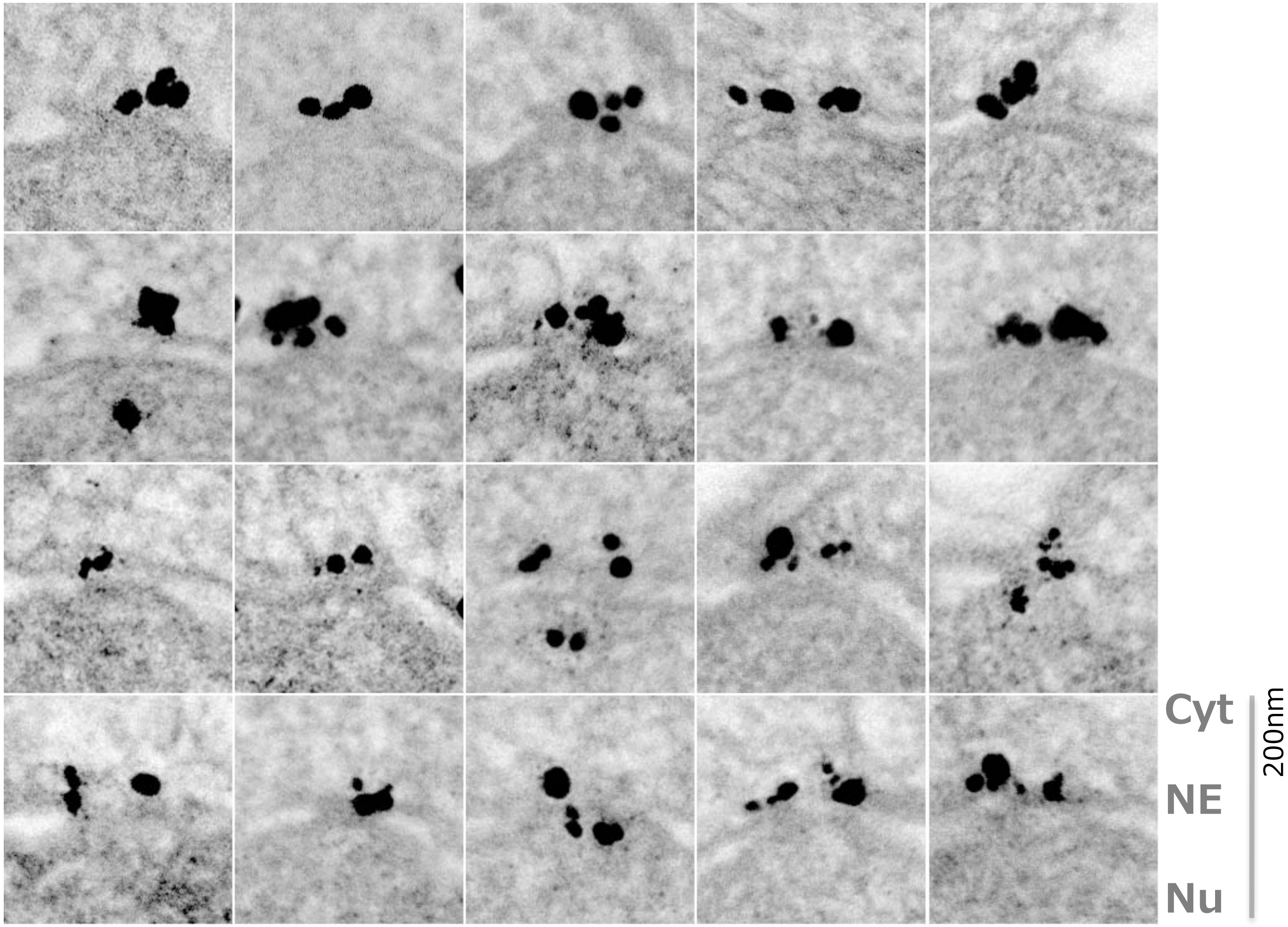

Nup37-GFP

(in cells expressing Mis6-GFP)

projection

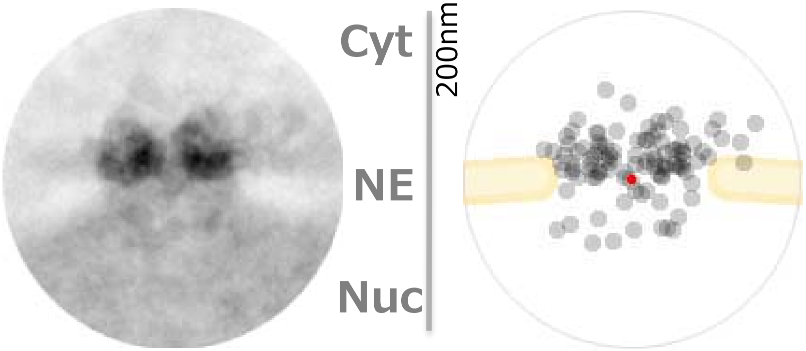

20 NPCs

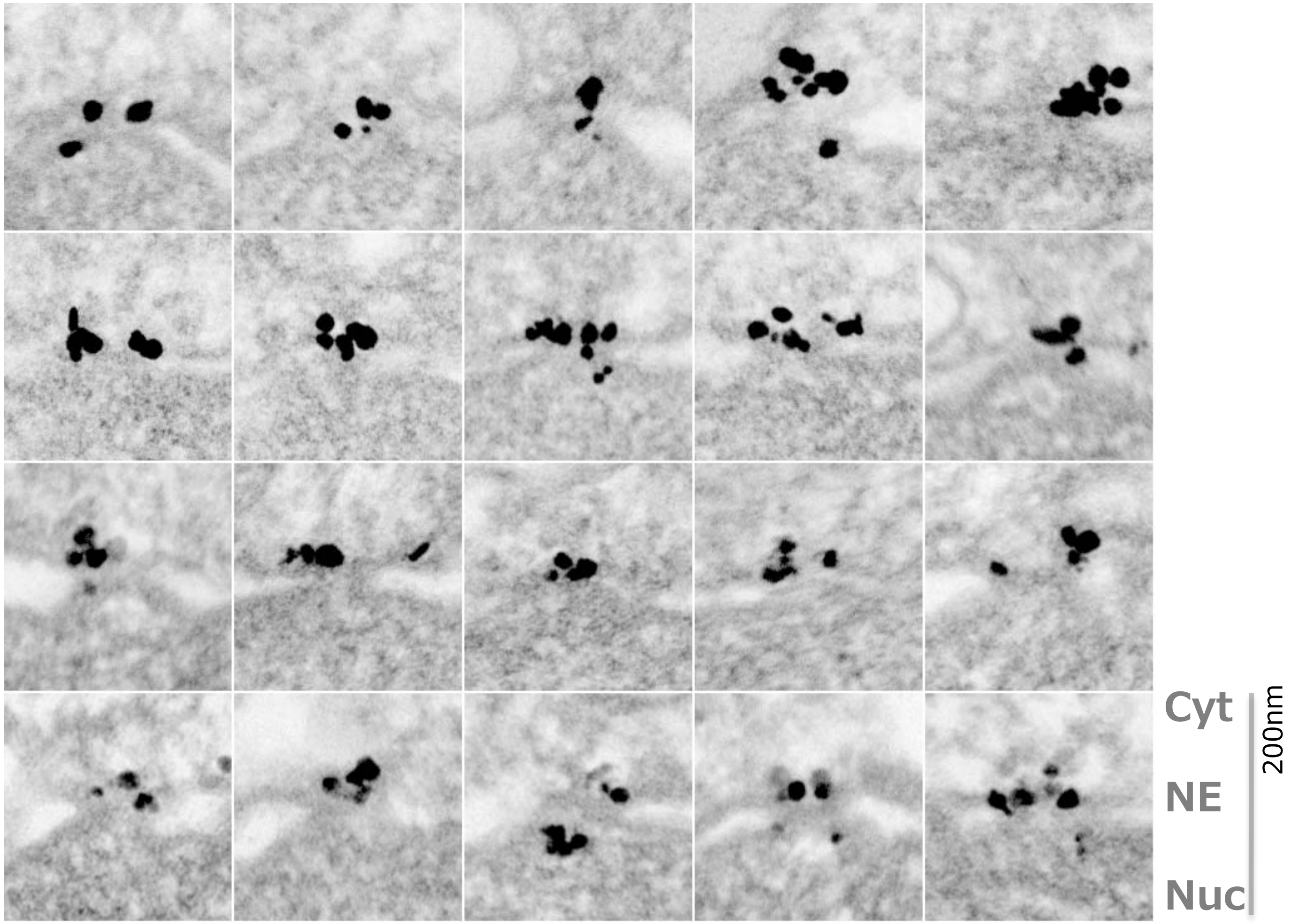

Ely5-GFP

projection

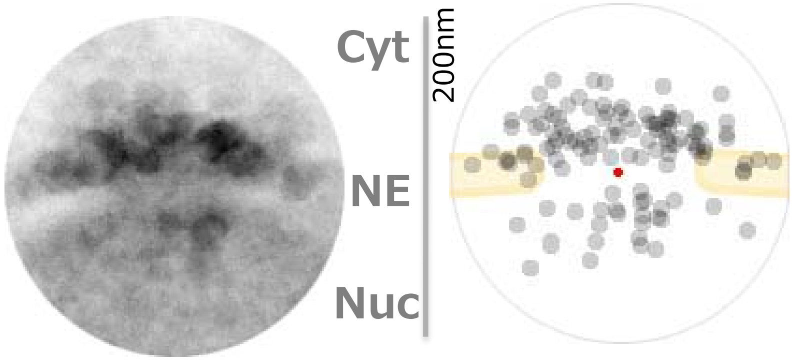

20 NPCs

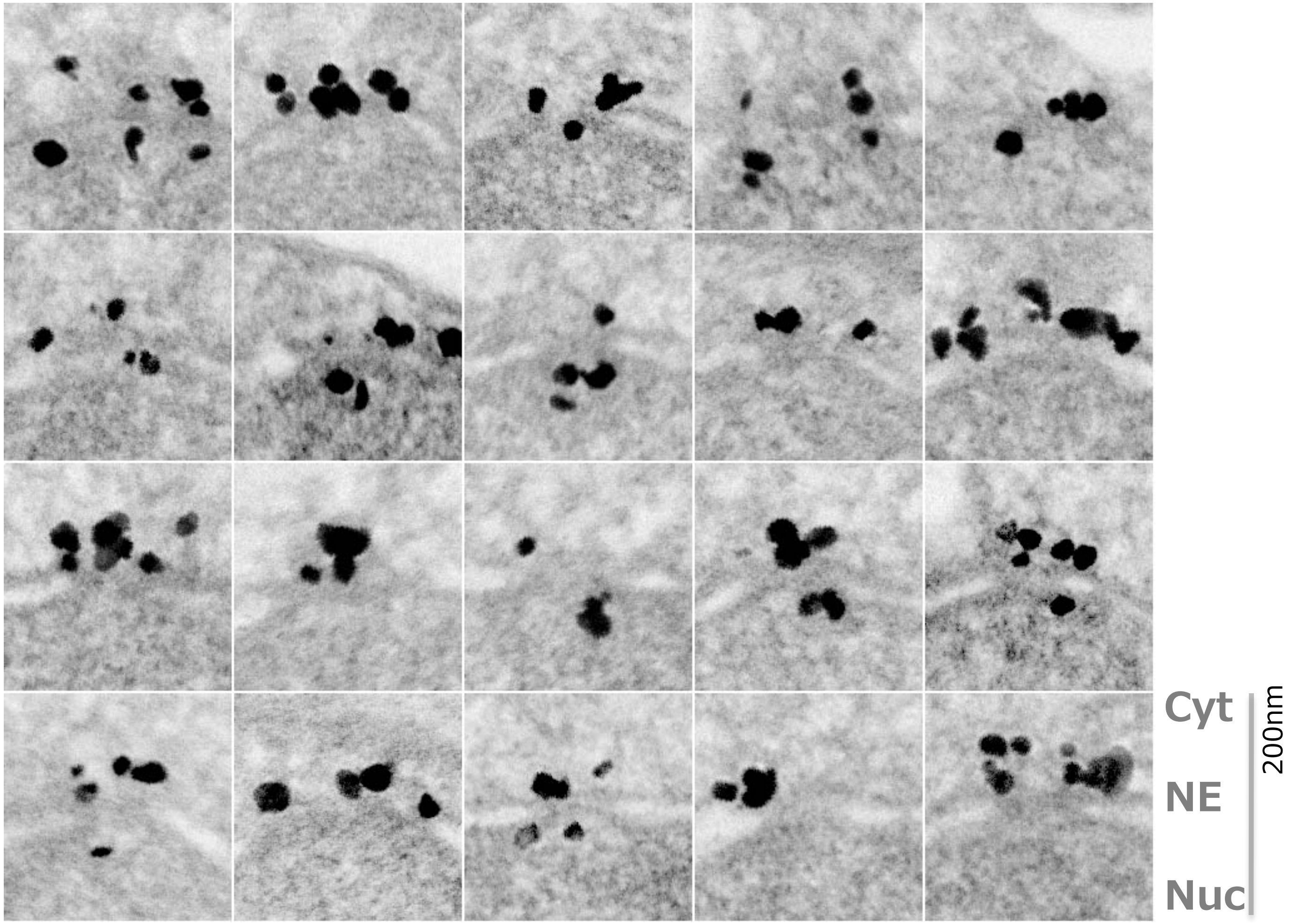

Ely5-GFP

(in cells expressing Mis6-GFP)

projection

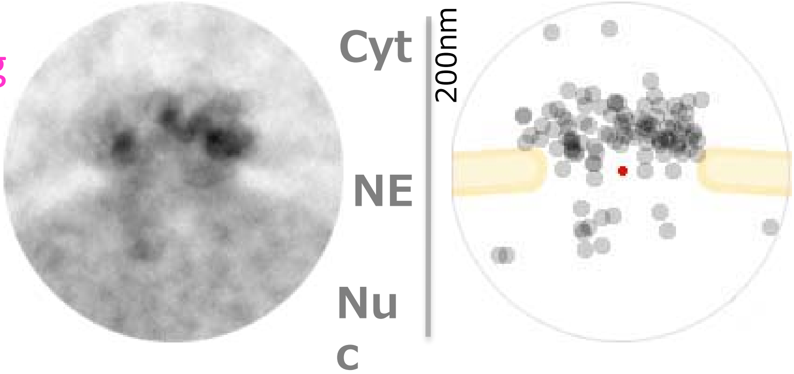

20 NPCs

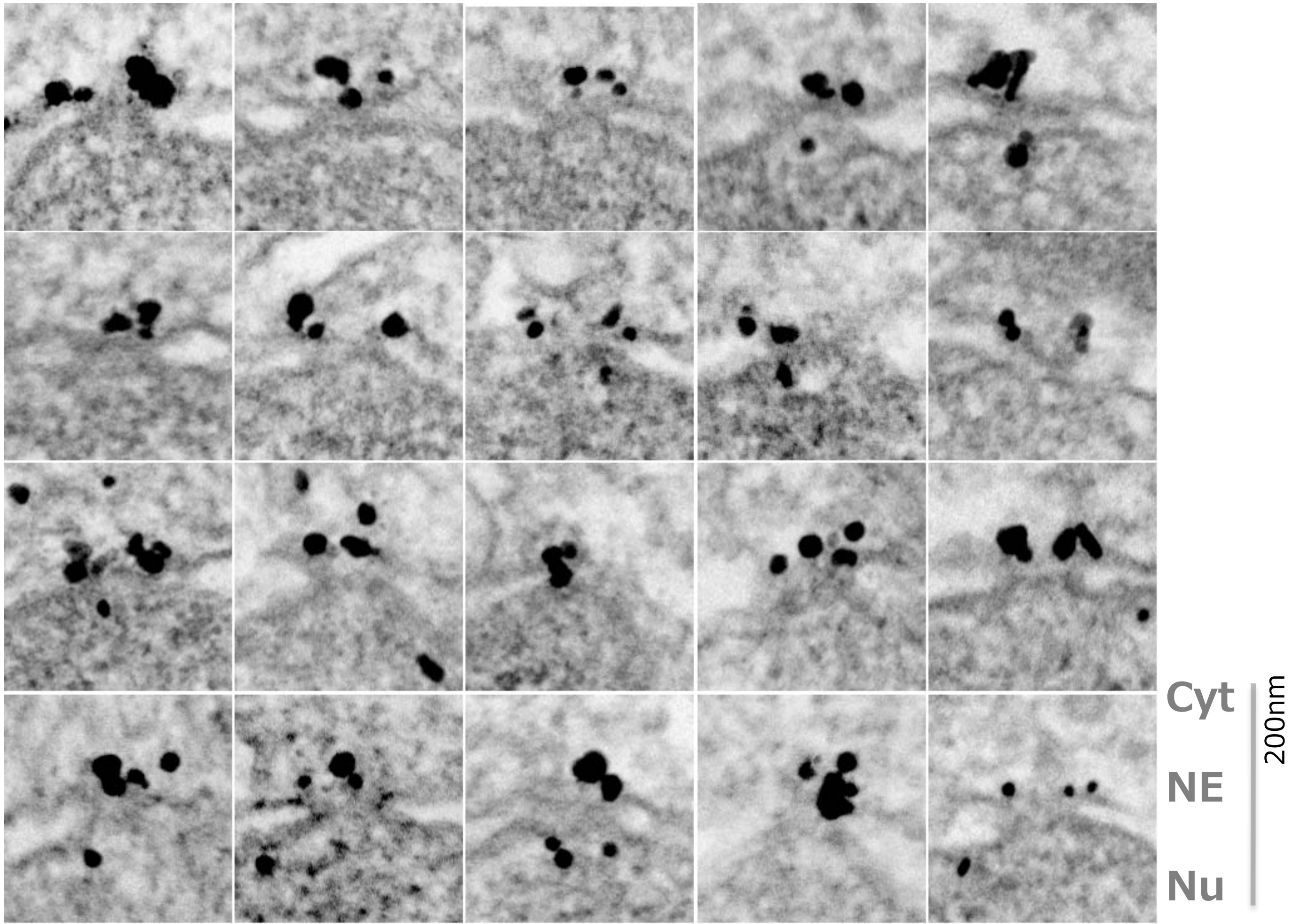

Seh1-GFP

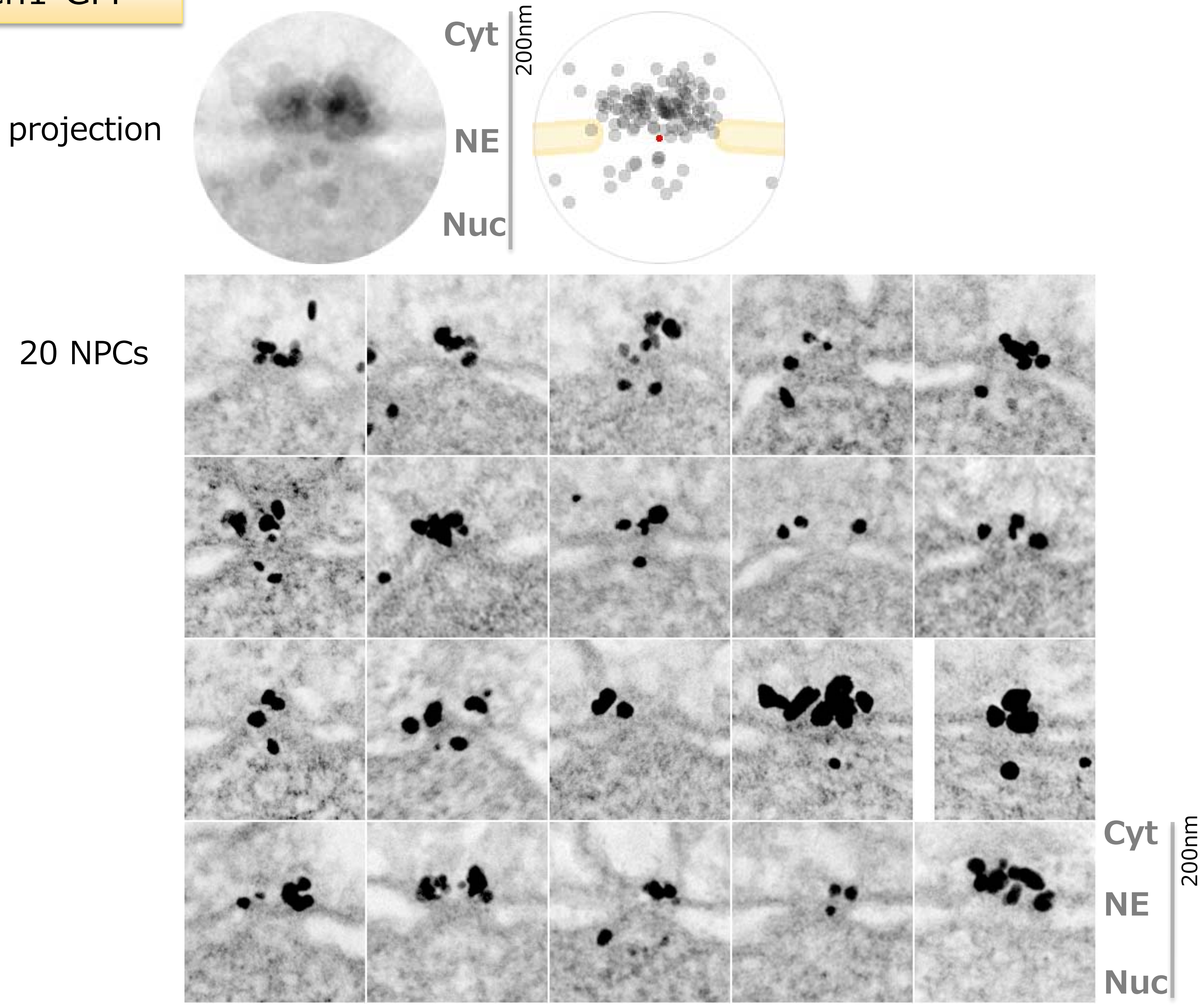

Seh1-GFP
(in cells expressing Mis6-GFP)
projection

20 NPCs

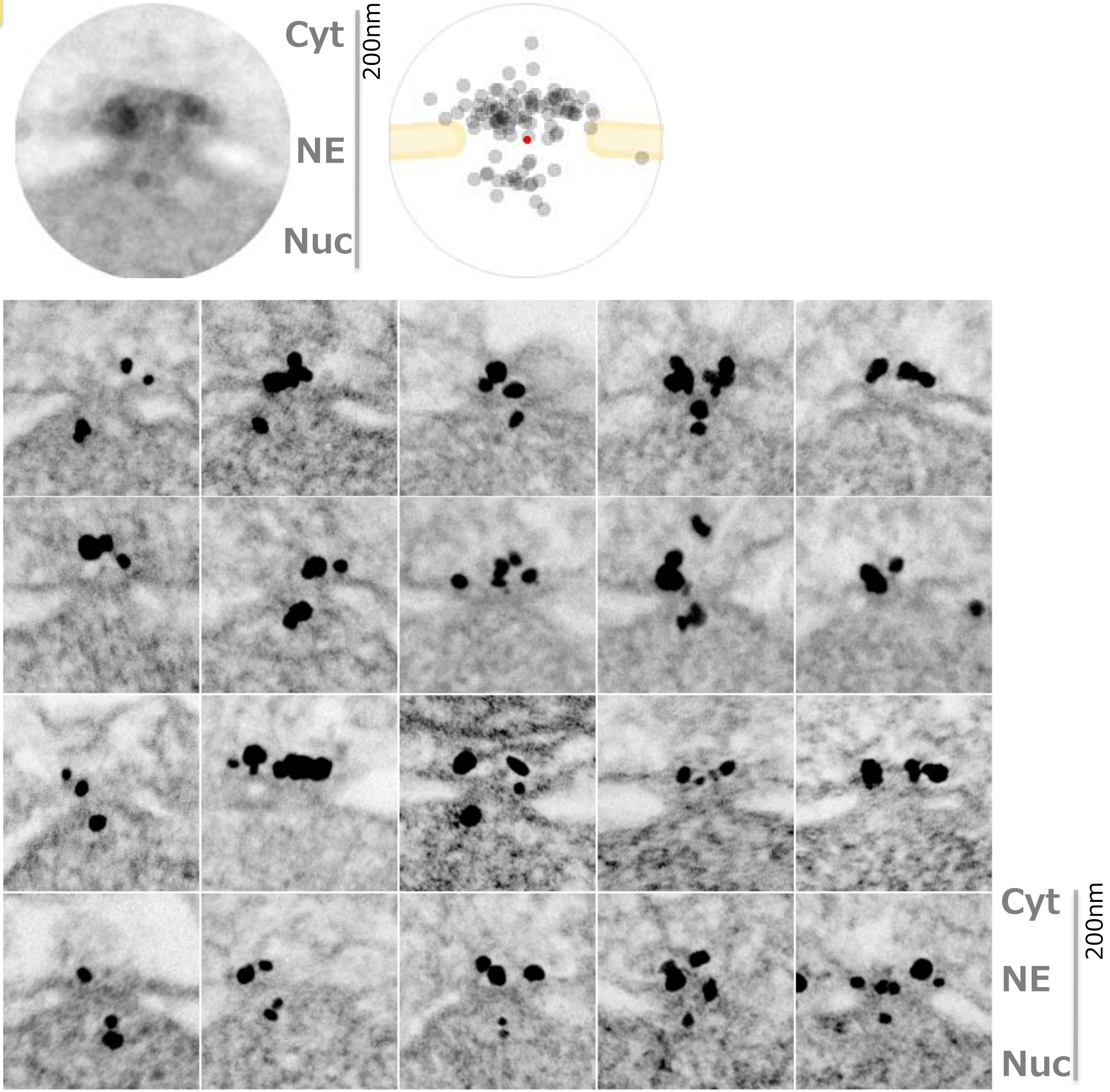

Nup107-GFP

projection

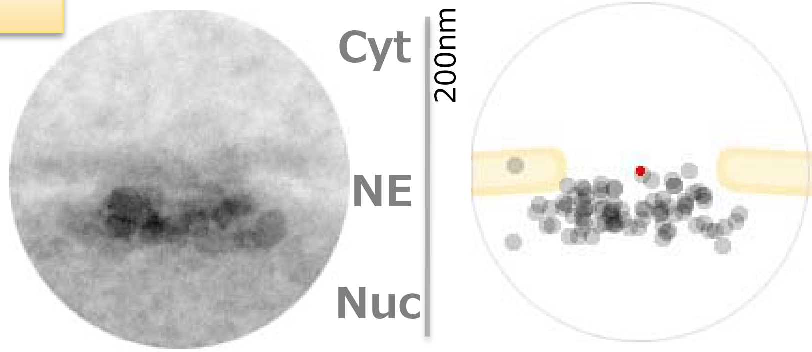

20 NPCs

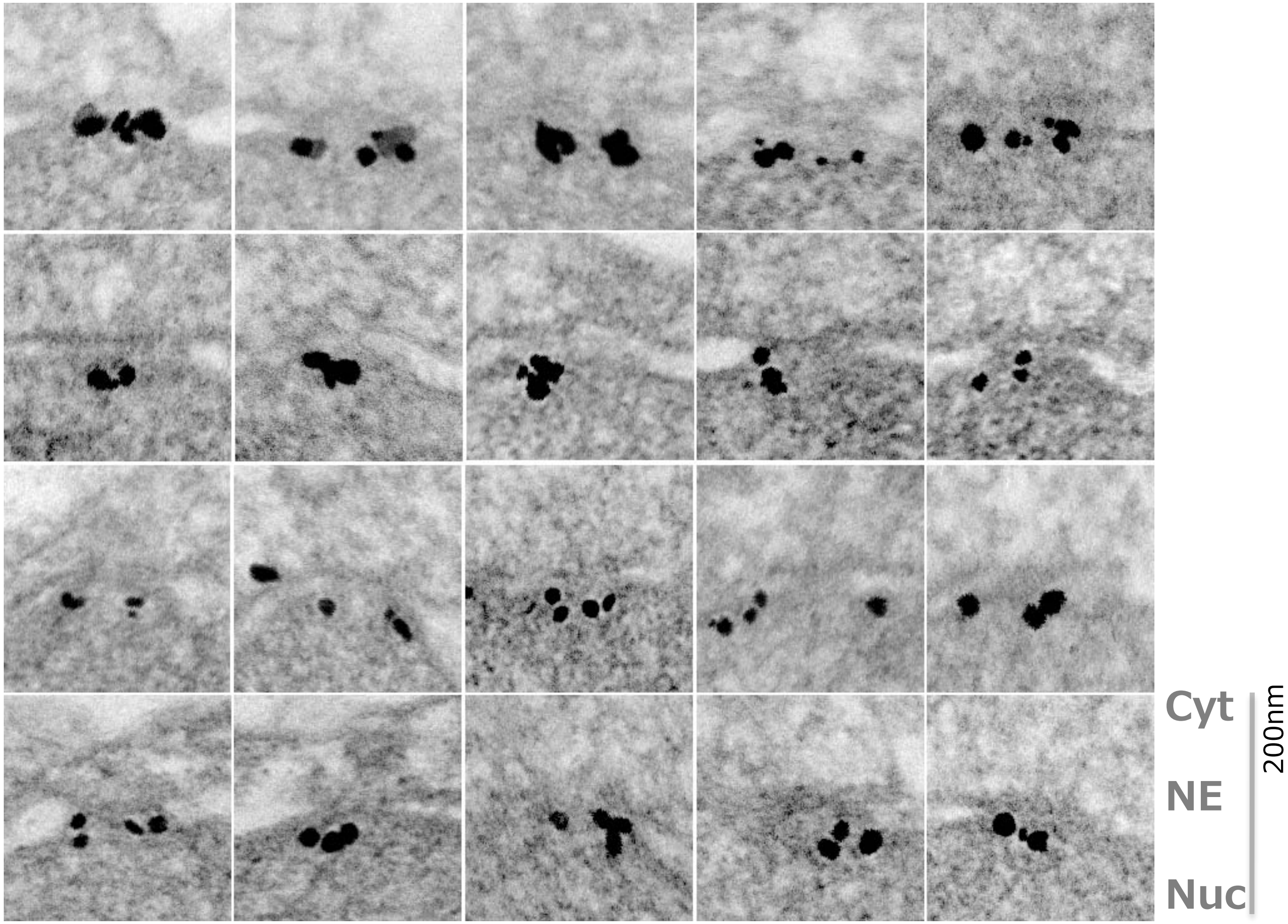

GFP-Nup107

projection

20 NPCs

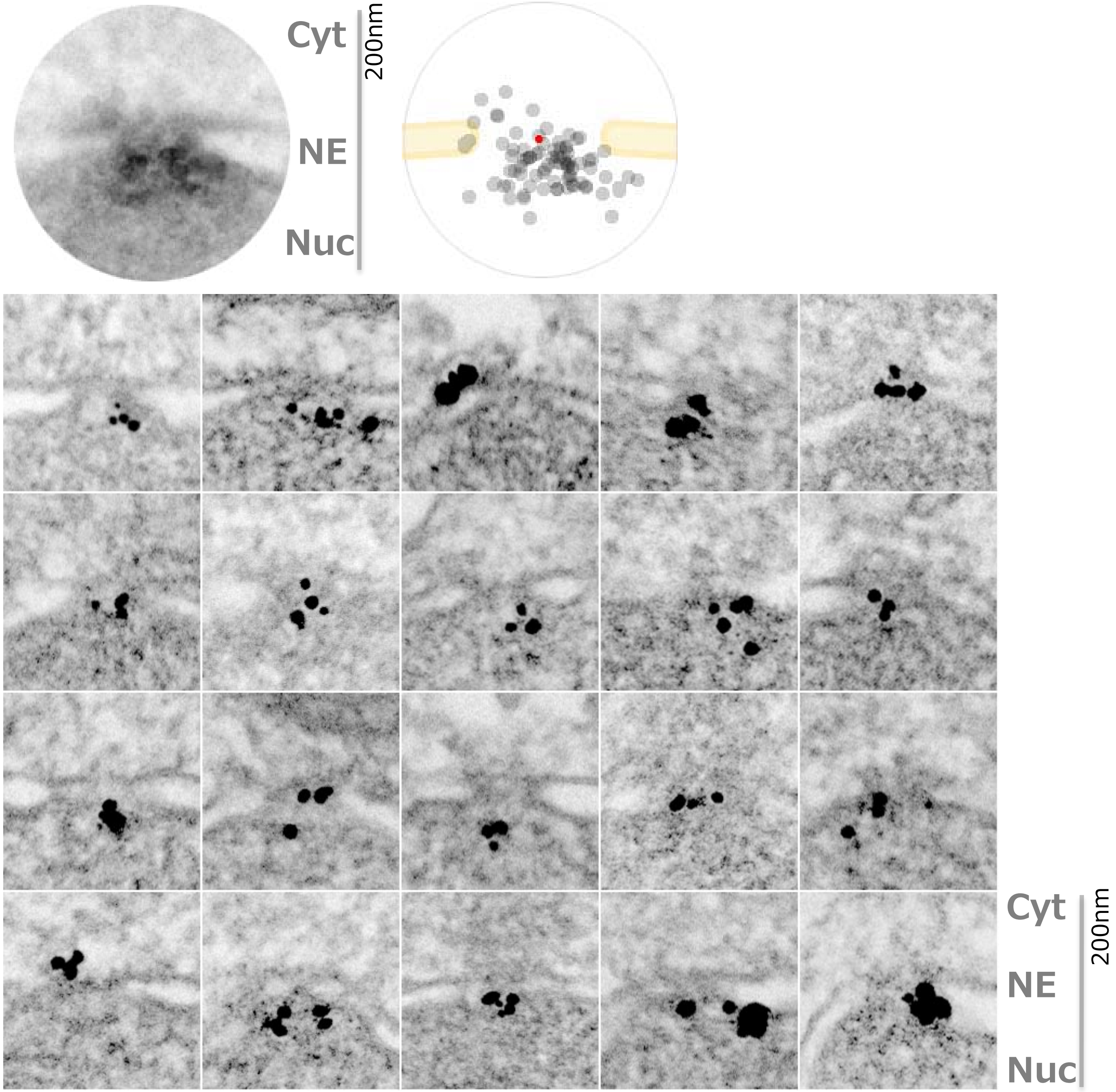

Supplement: S6 Dataset — (PDF) [file pgen.1008061.s017.pdf]
