# Supplementary material for: Asymmetrical localization of Nup107-160 subcomplex components within the nuclear pore complex in fission yeast
Source: PLoS Genet. 2019 Jun 6;15(6):e1008061. doi: 10.1371/journal.pgen.1008061 (PMC6553703; doi:10.1371/journal.pgen.1008061)

# S9 Dataset

Individual IEM images of 20 NPCs used for superimposed images of Figure 5b (spNup96-spNup107-GFP) and 5c (GFP-spNup132 in the presence of the spNup96-spNup107 fusion protein)

Nup96-Nup107-GFP
(*nup107Δ* background)

(in cells expressing
Mis6-GFP)

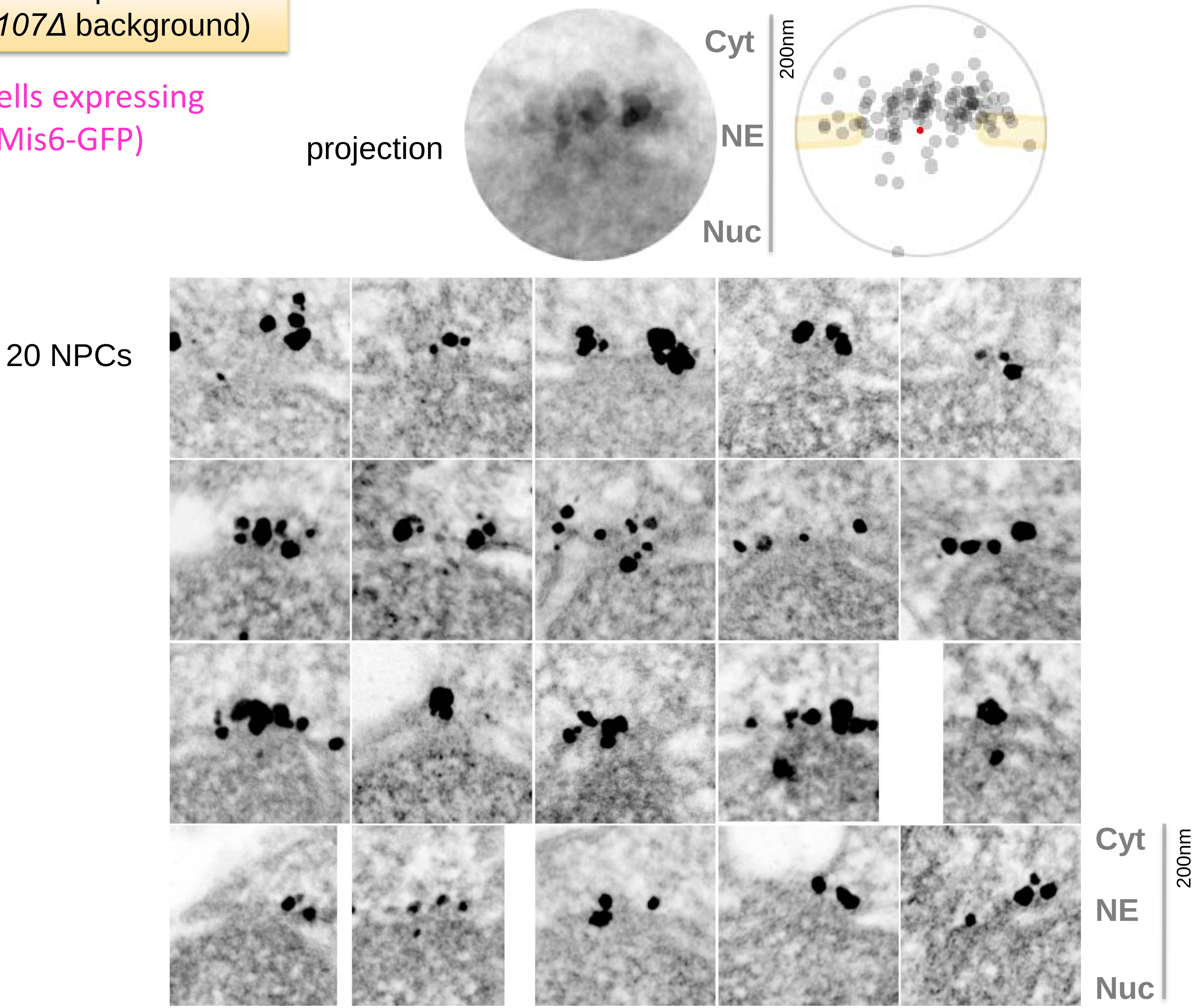

GFP-Nup132
(Nup96-Nup107,
*nup107Δ* background)

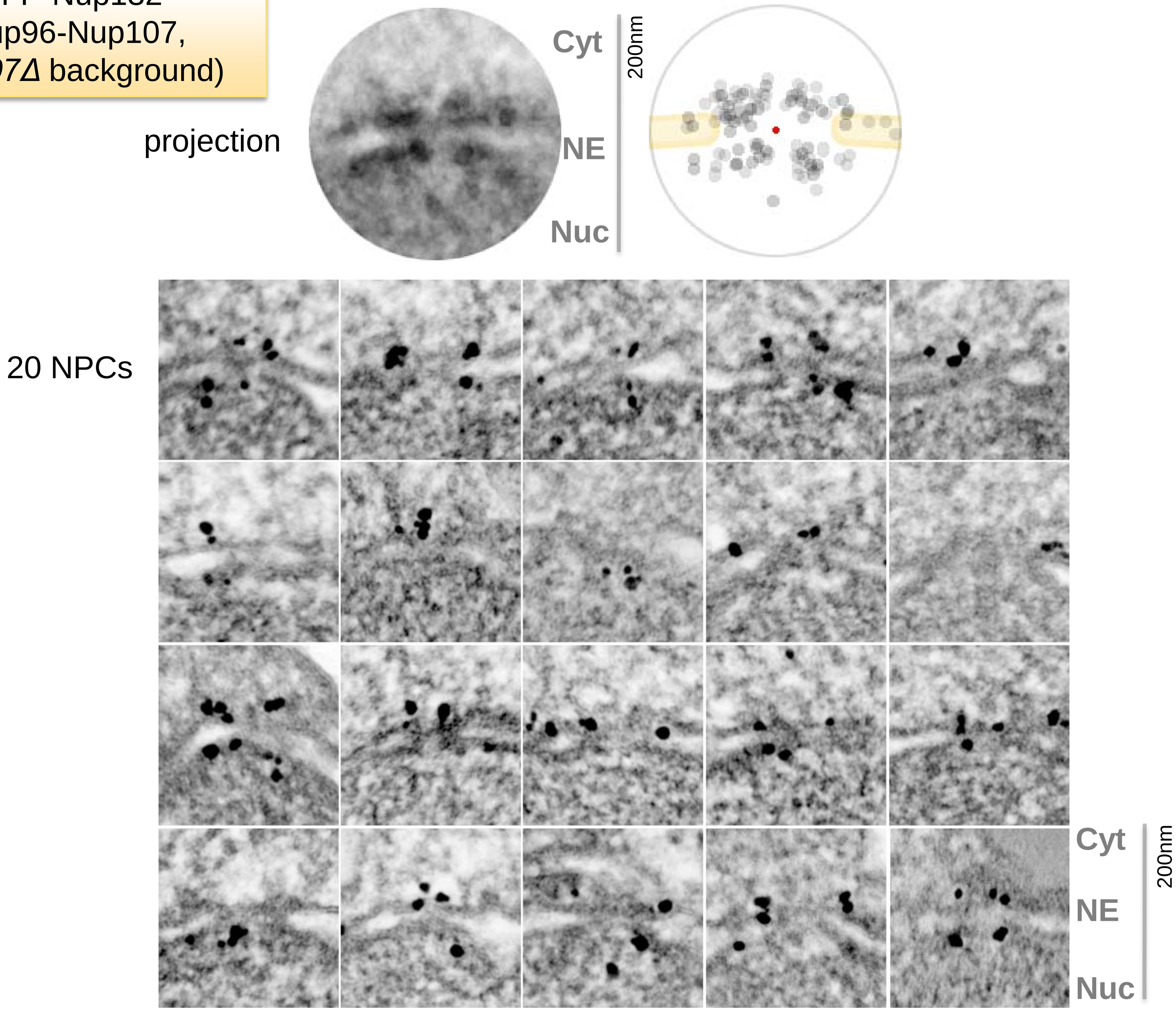

Supplement: S9 Dataset — (PDF) [file pgen.1008061.s020.pdf]
