# Supplementary material for: Asymmetrical localization of Nup107-160 subcomplex components within the nuclear pore complex in fission yeast
Source: PLoS Genet. 2019 Jun 6;15(6):e1008061. doi: 10.1371/journal.pgen.1008061 (PMC6553703; doi:10.1371/journal.pgen.1008061)

S10 Dataset

Individual IEM images of 20 NPCs used for superimposed images of Figure 6c (full length GFP-spNup131 and GFP-spNup132, and their C-terminal domains in the *nup131Δnup132Δ* mutant cells)

GFP-Nup131FL
(*nup131Δ nup132Δ* background)

projection

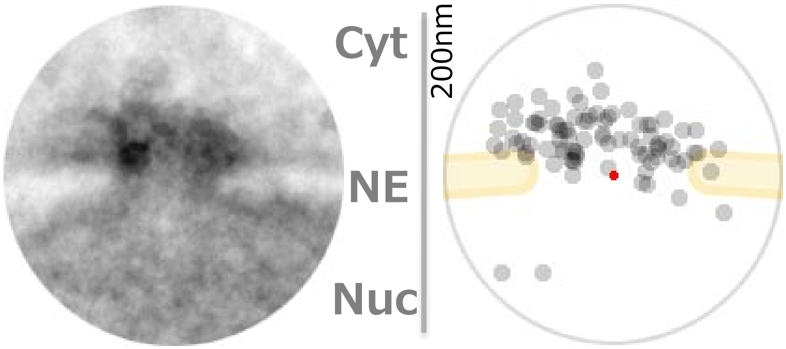

20 NPCs

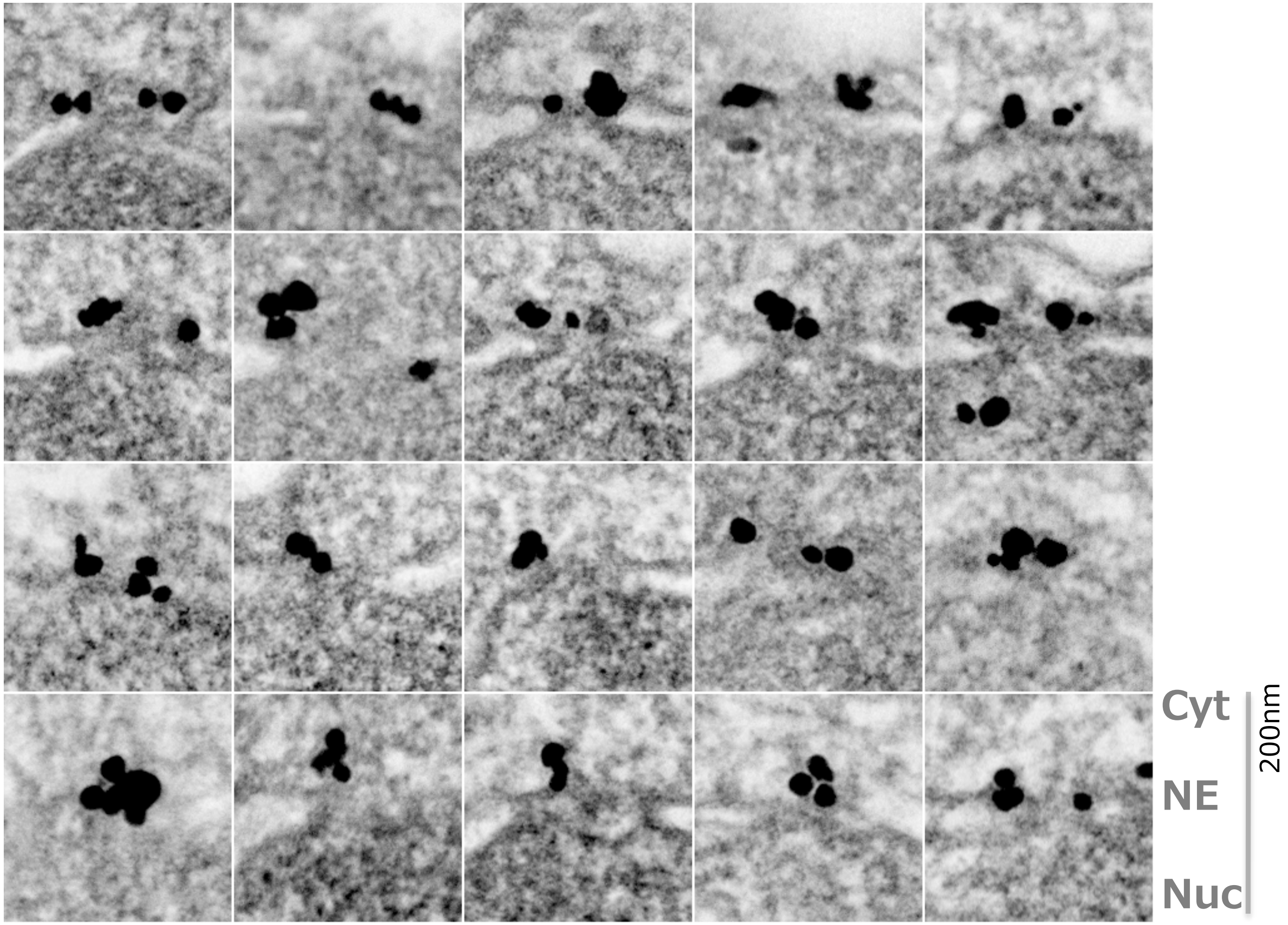

GFP-Nup131C
(*nup131Δ nup132Δ*
background)

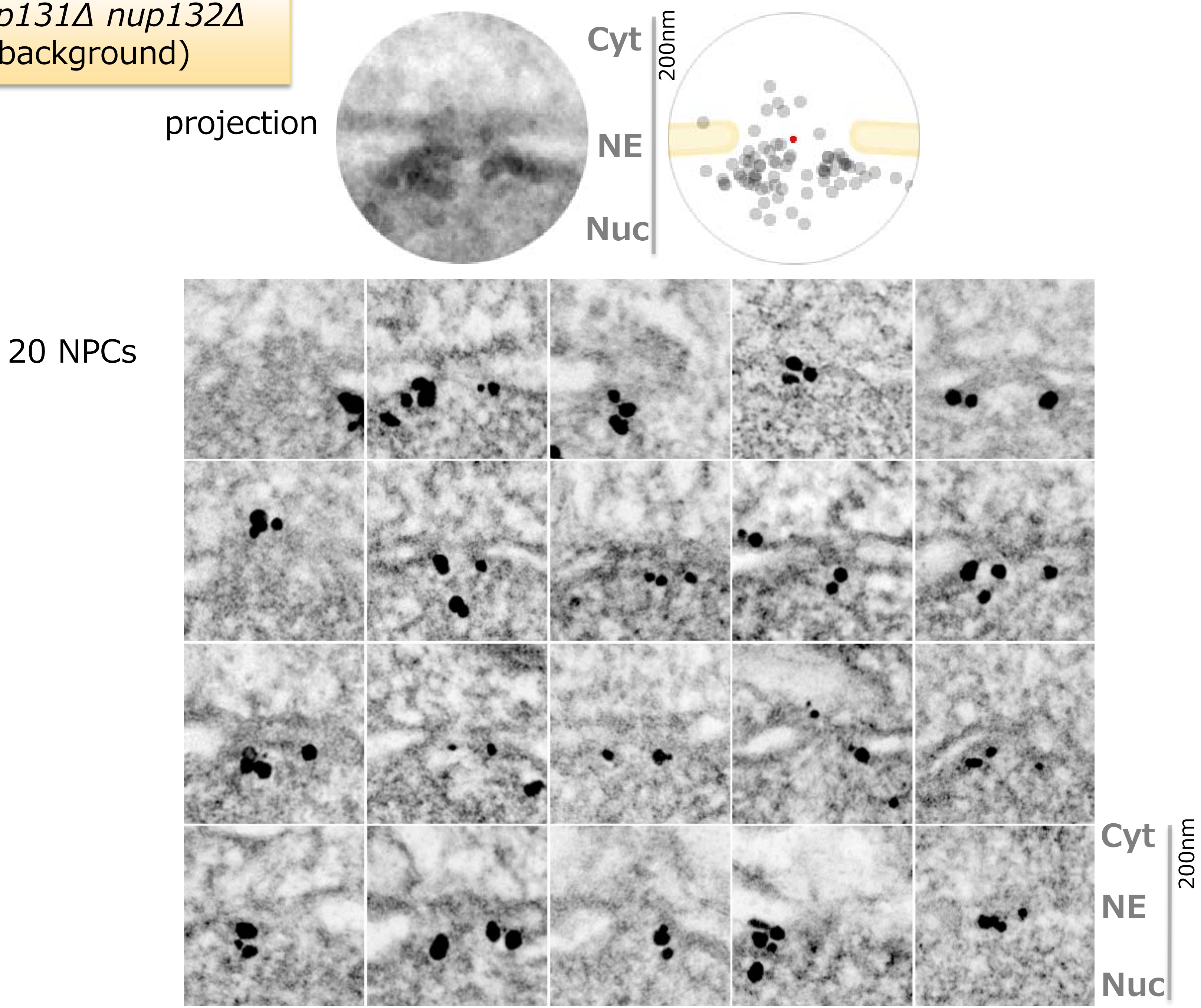

GFP-Nup132FL
(*nup131Δ nup132Δ* background)

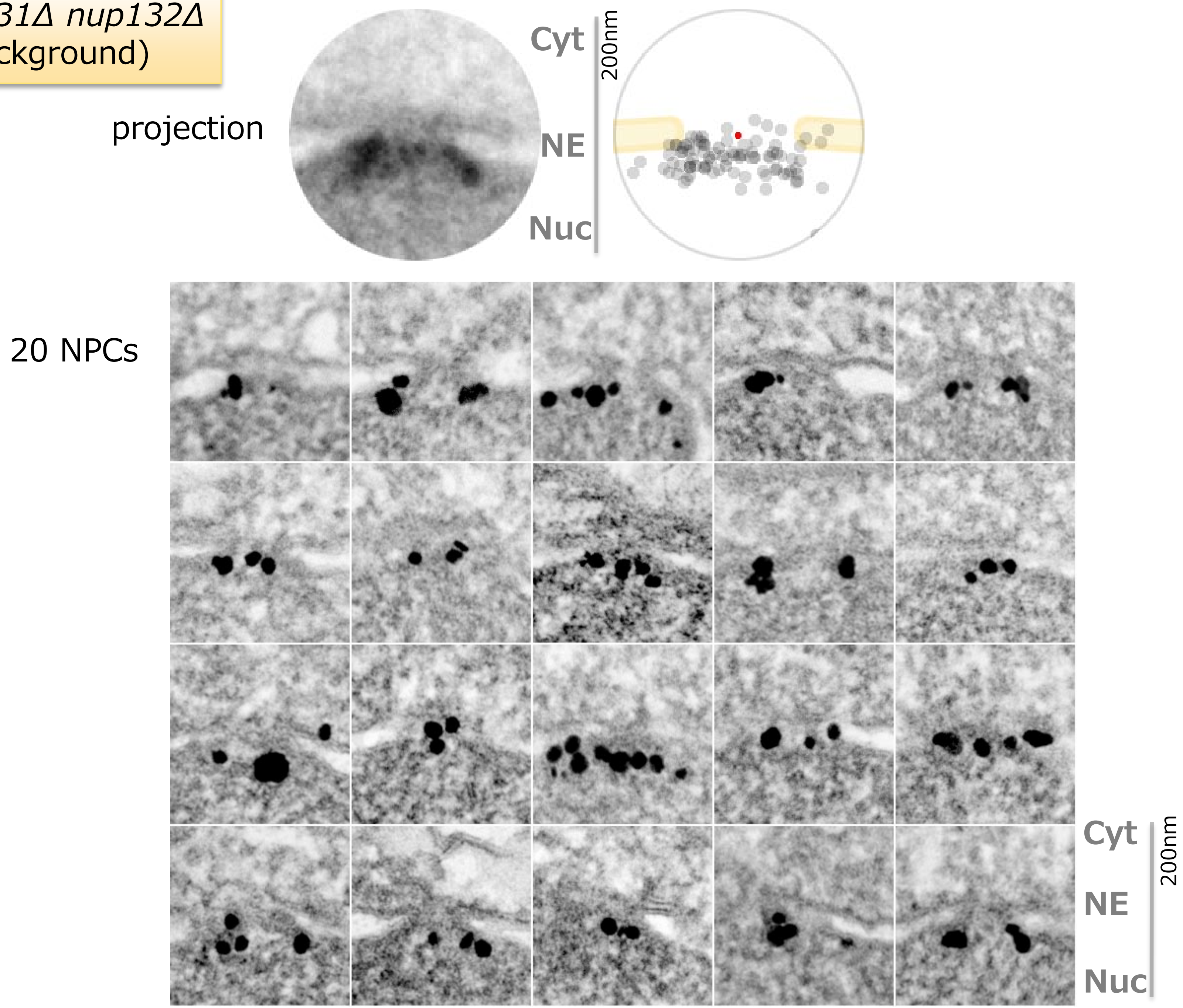

GFP-Nup132C
(*nup131Δ nup132Δ*
background)

projection

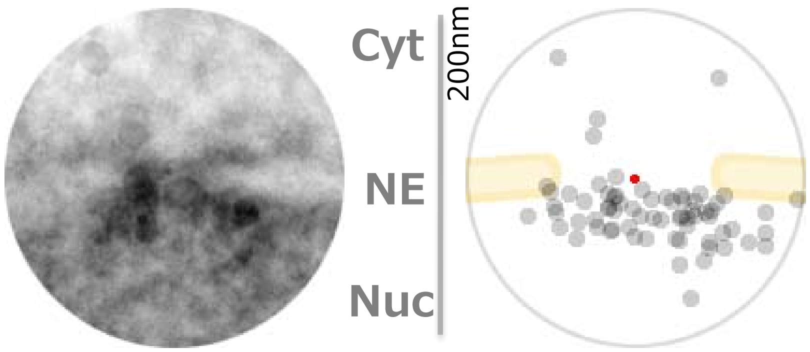

20 NPCs

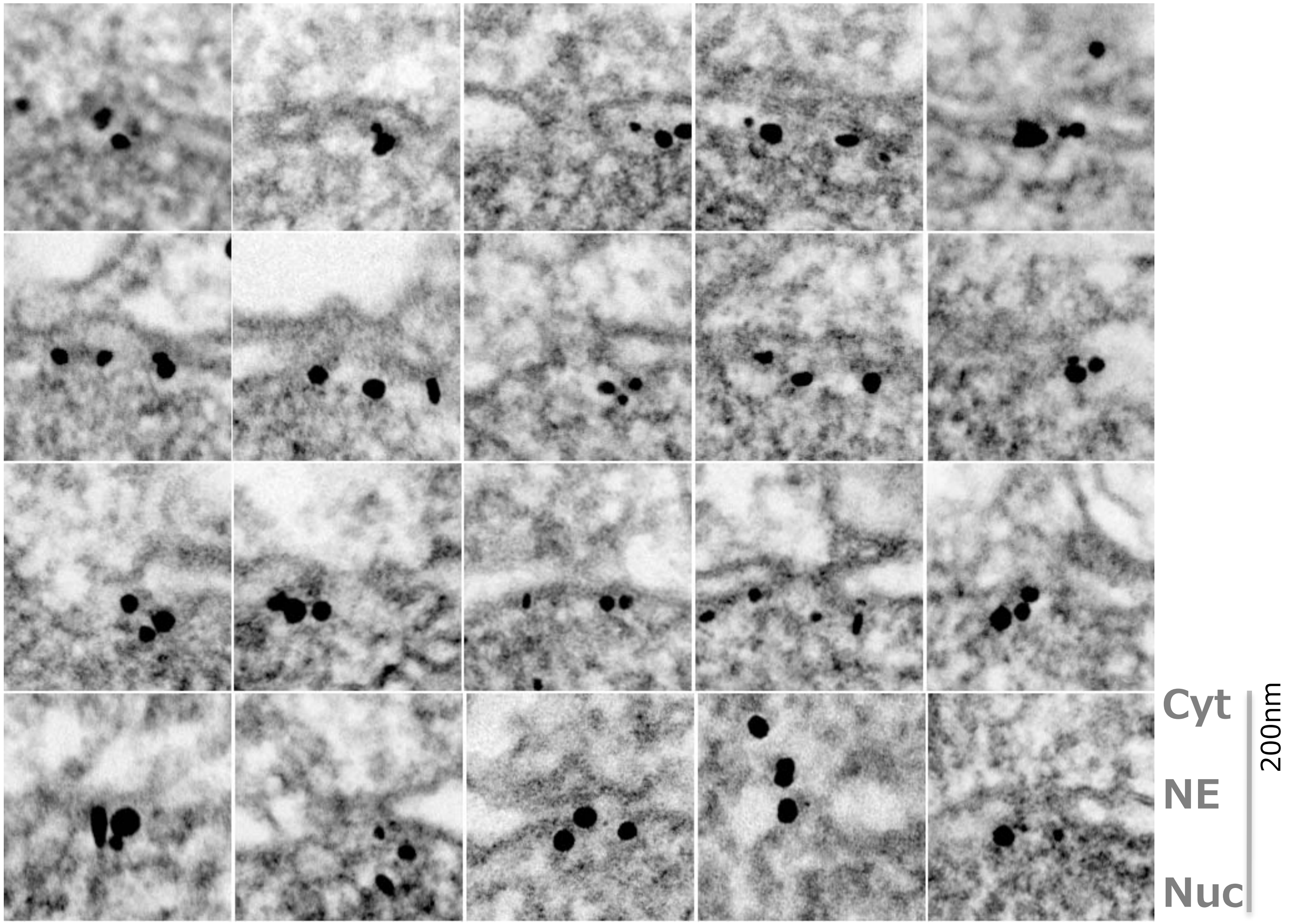

Supplement: S10 Dataset — (PDF) [file pgen.1008061.s021.pdf]
