# Supplementary material for: Asymmetrical localization of Nup107-160 subcomplex components within the nuclear pore complex in fission yeast
Source: PLoS Genet. 2019 Jun 6;15(6):e1008061. doi: 10.1371/journal.pgen.1008061 (PMC6553703; doi:10.1371/journal.pgen.1008061)

S11 Dataset

Individual IEM images of 20 NPCs used for superimposed images of Figure 7 (inner ring Nups, channel Nups, cytoplasmic ring Nups, transmembrane Nups and nuclear basket Nups)

Nup93 subcomplex Nups

Nup97,Npp106,Nup184,Nup186, Nup40, Nup155

GFP−Nup97

projection

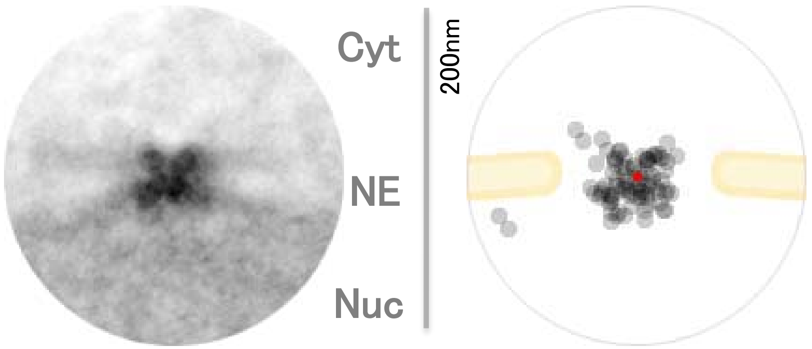

20 NPCs

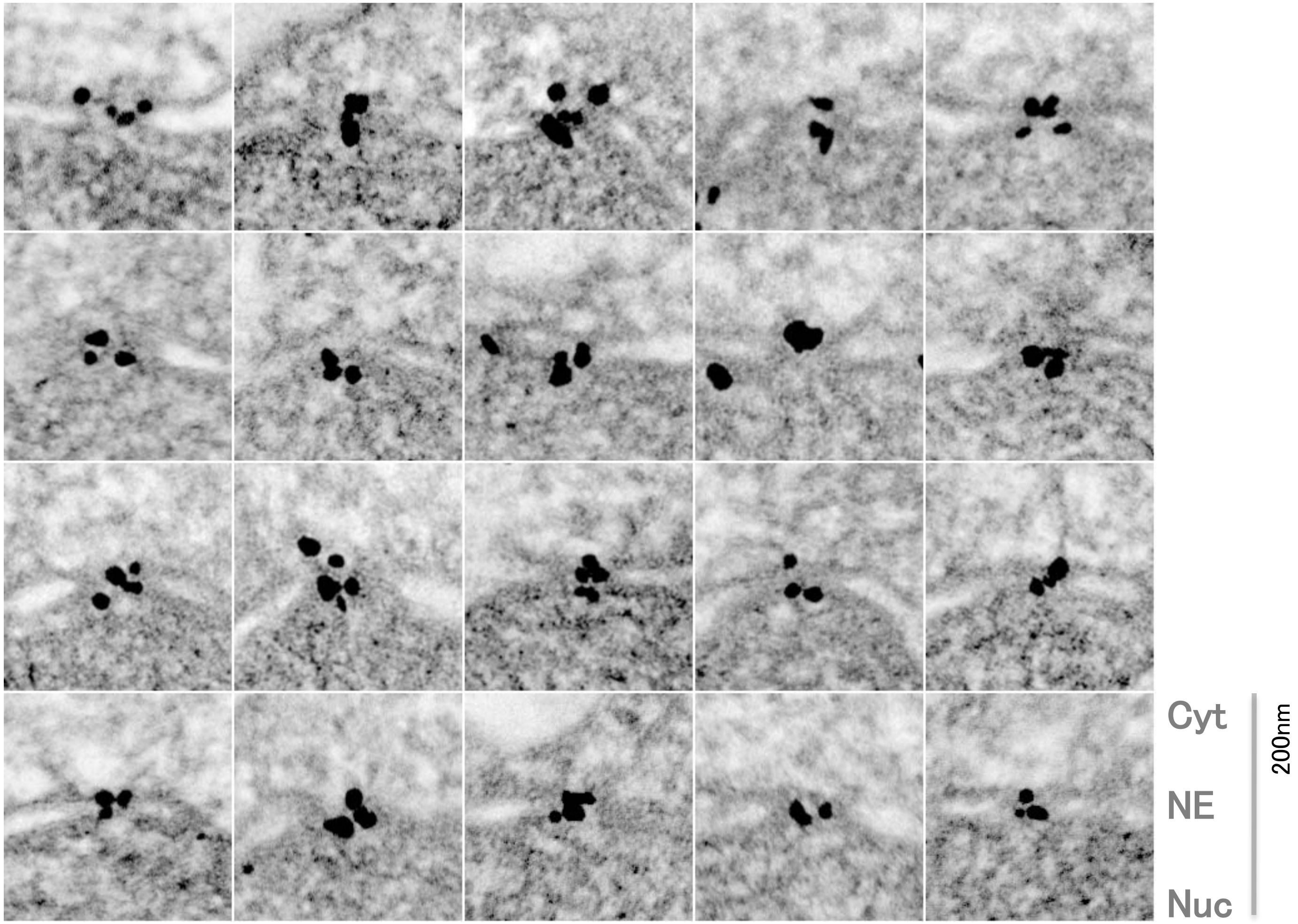

GFP−Npp106

projection

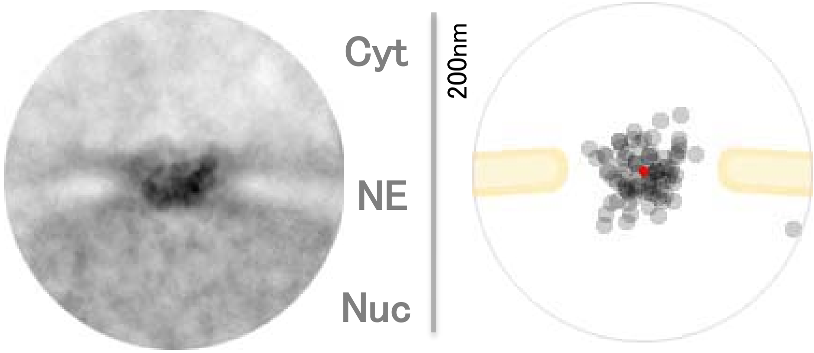

20 NPCs

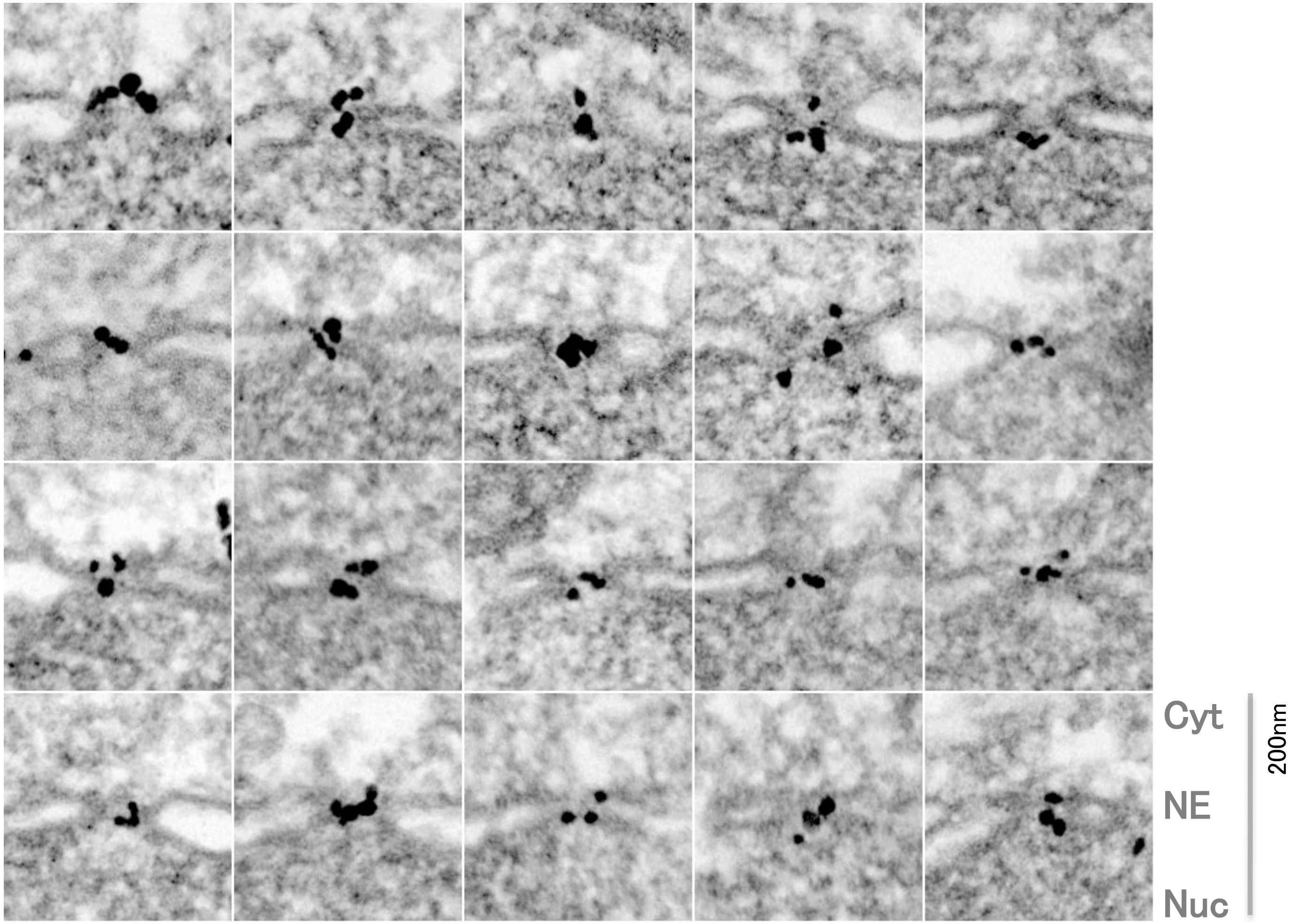

GFP-Nup184

projection

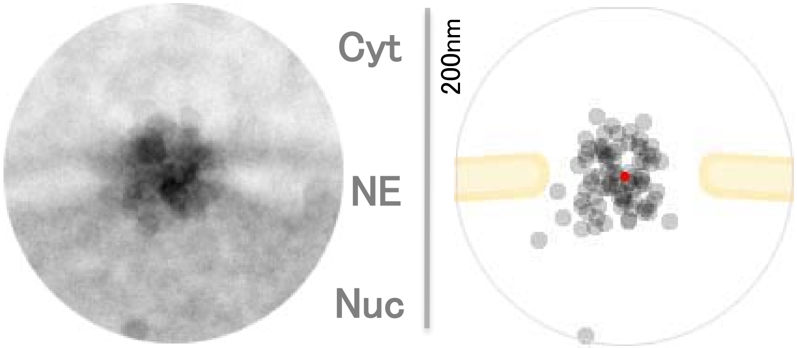

20 NPCs

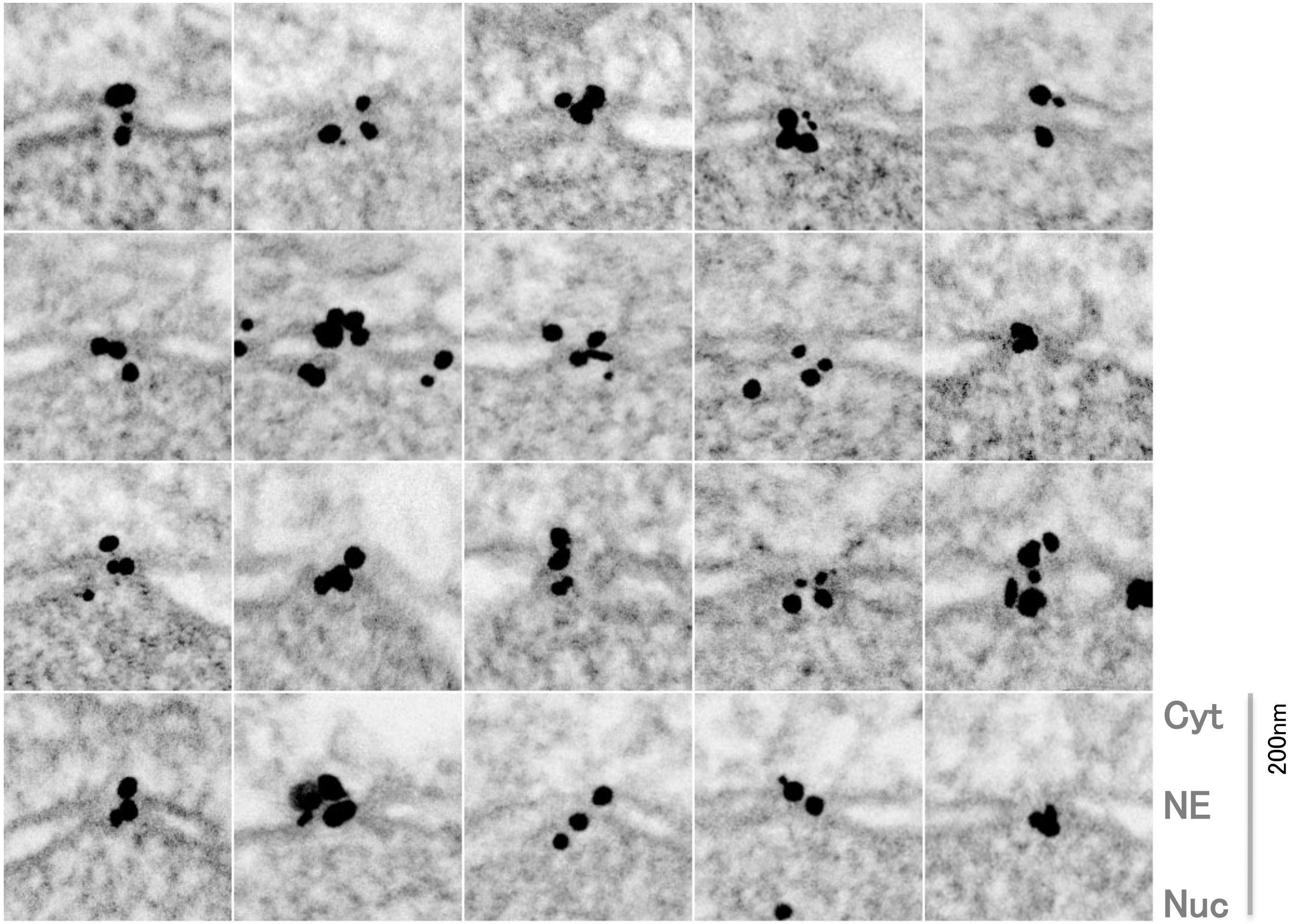

GFP–Nup186

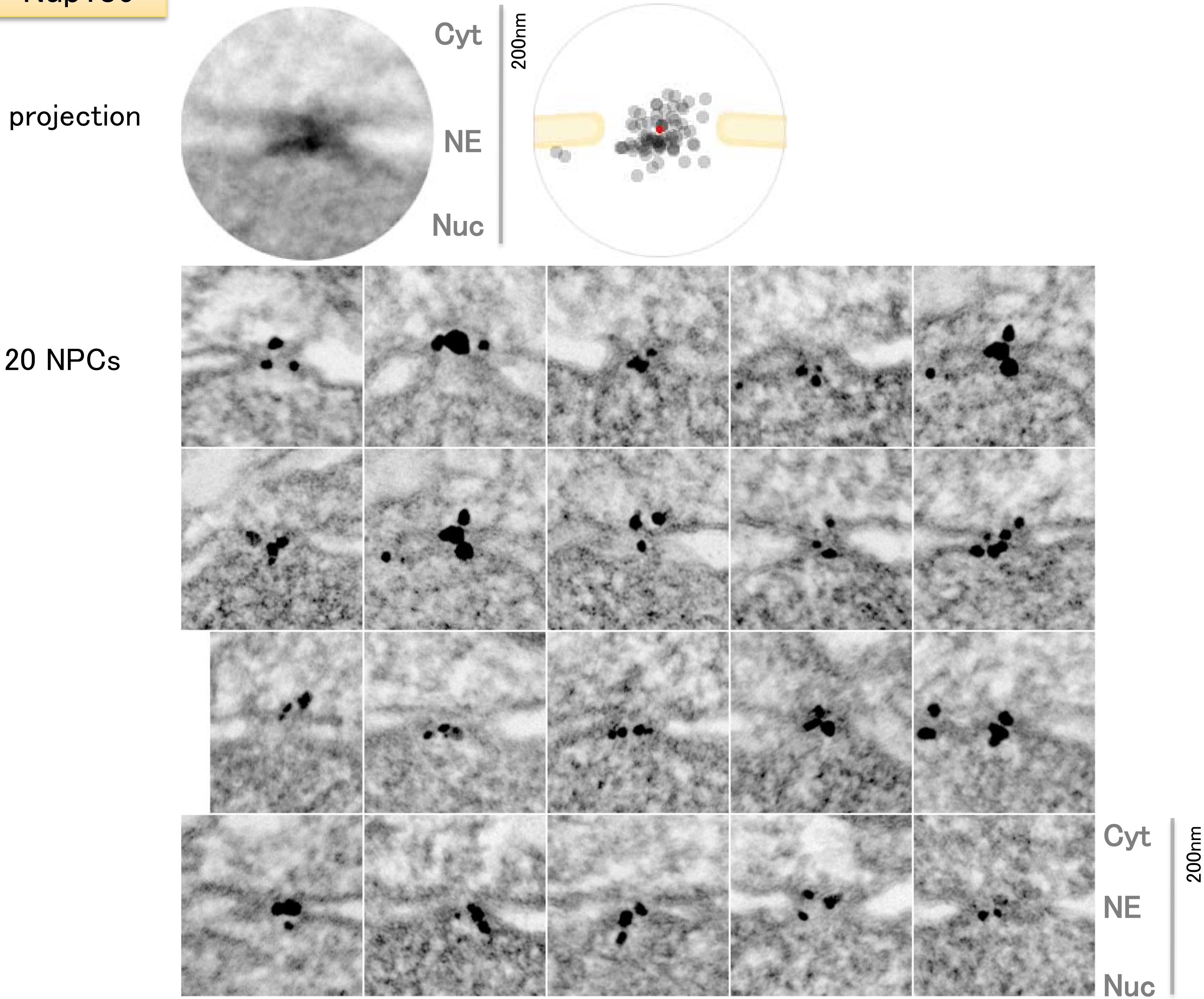

GFP–Nup40

projection

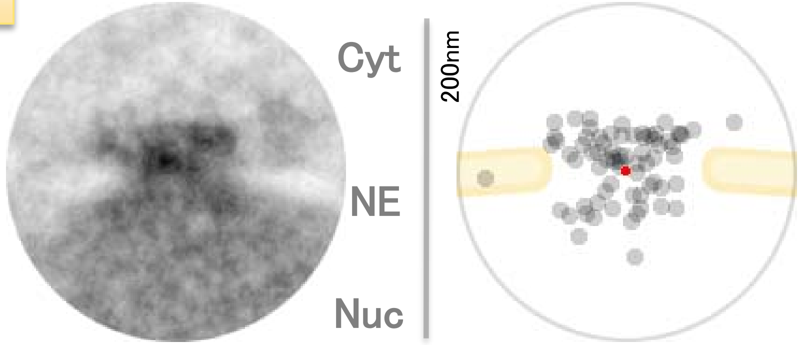

20 NPCs

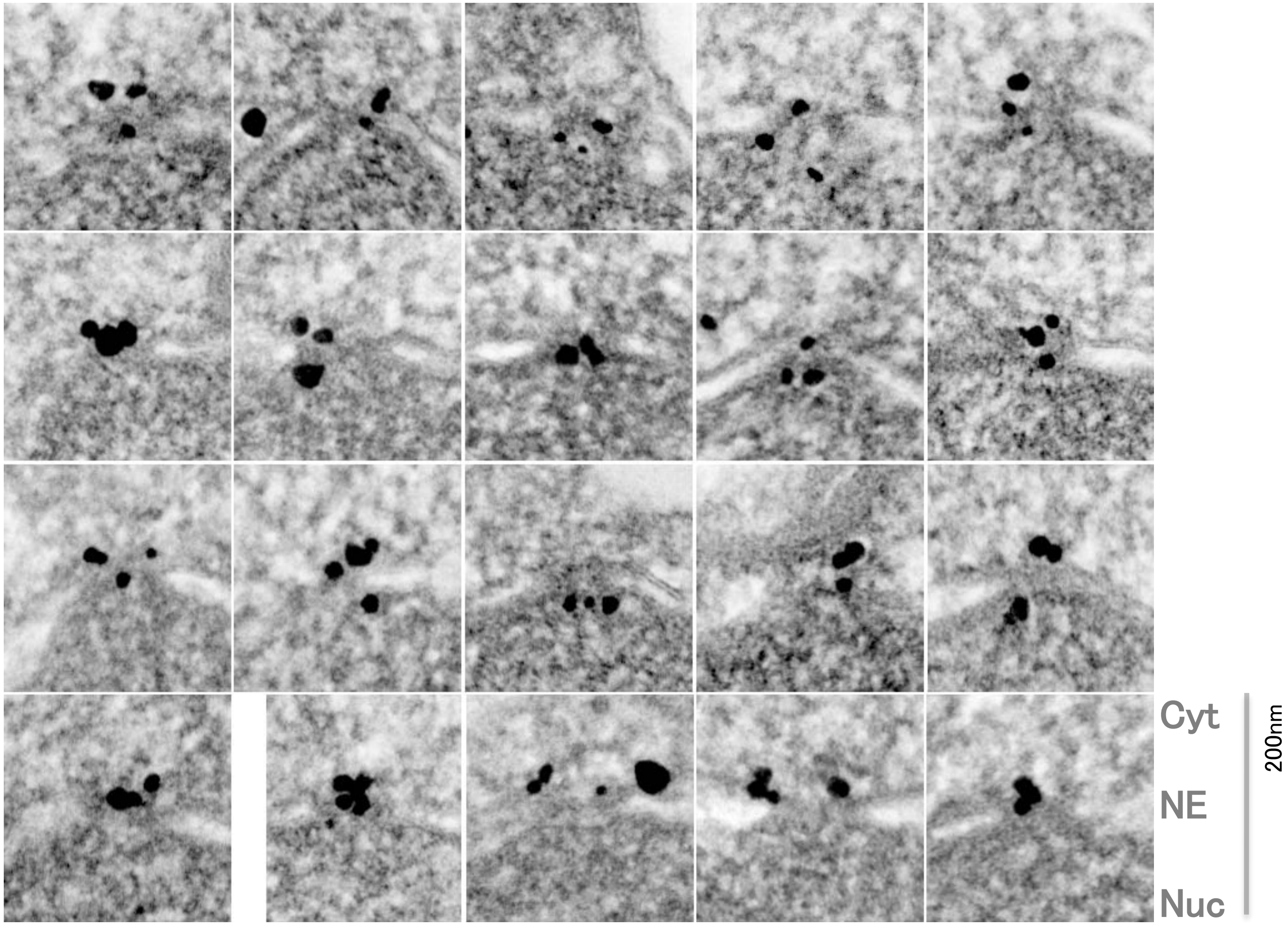

Nup155-GFP

projection

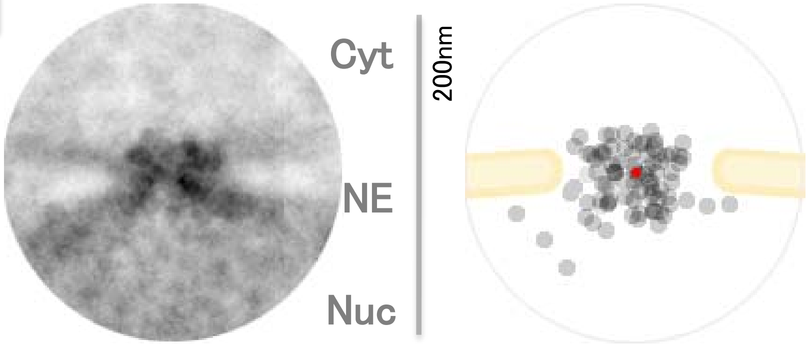

20 NPCs

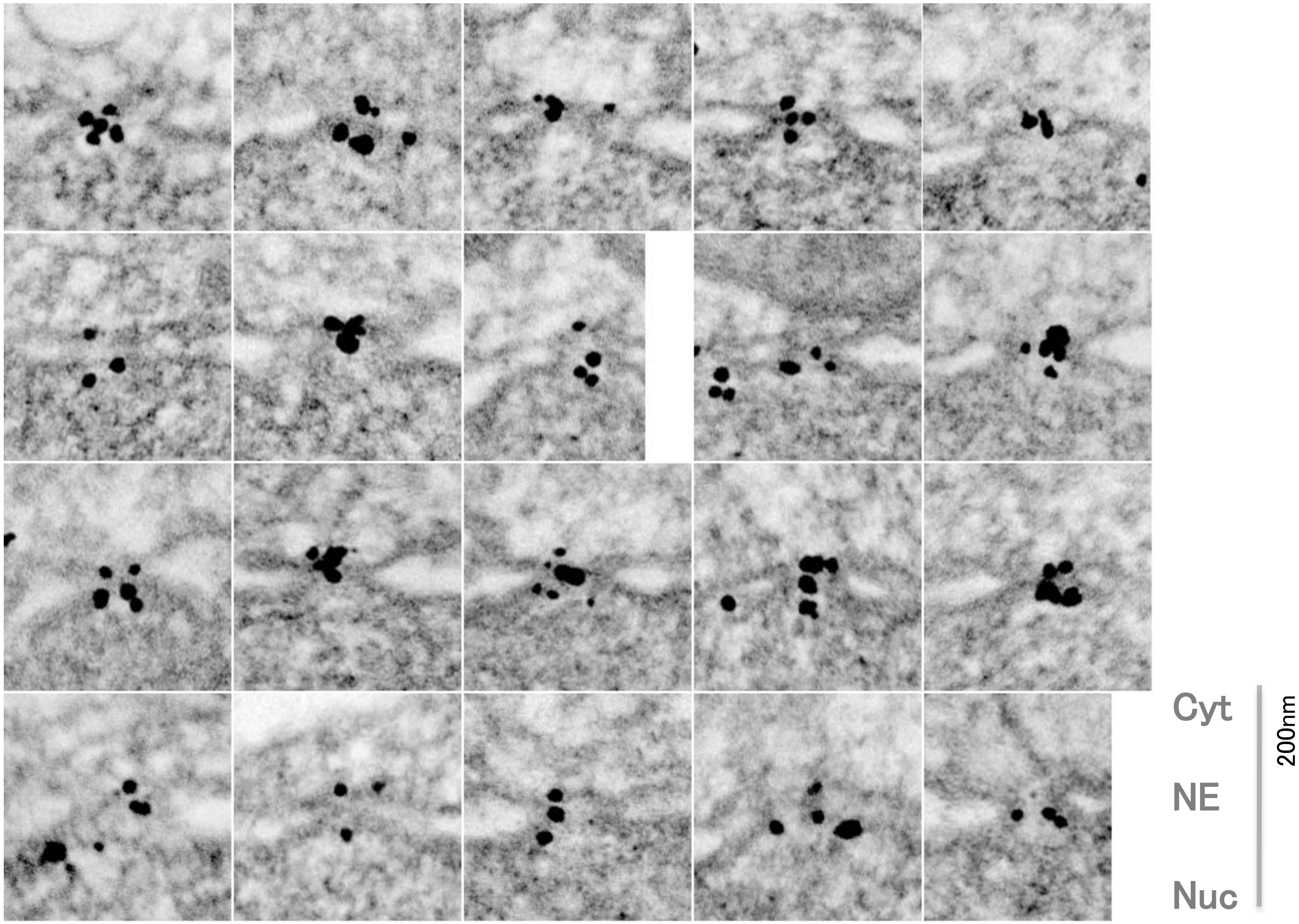

Channel Nups

Nup44,Nup45, Nup98, Nsp1

Nup44-GFP

projection

20 NPCs

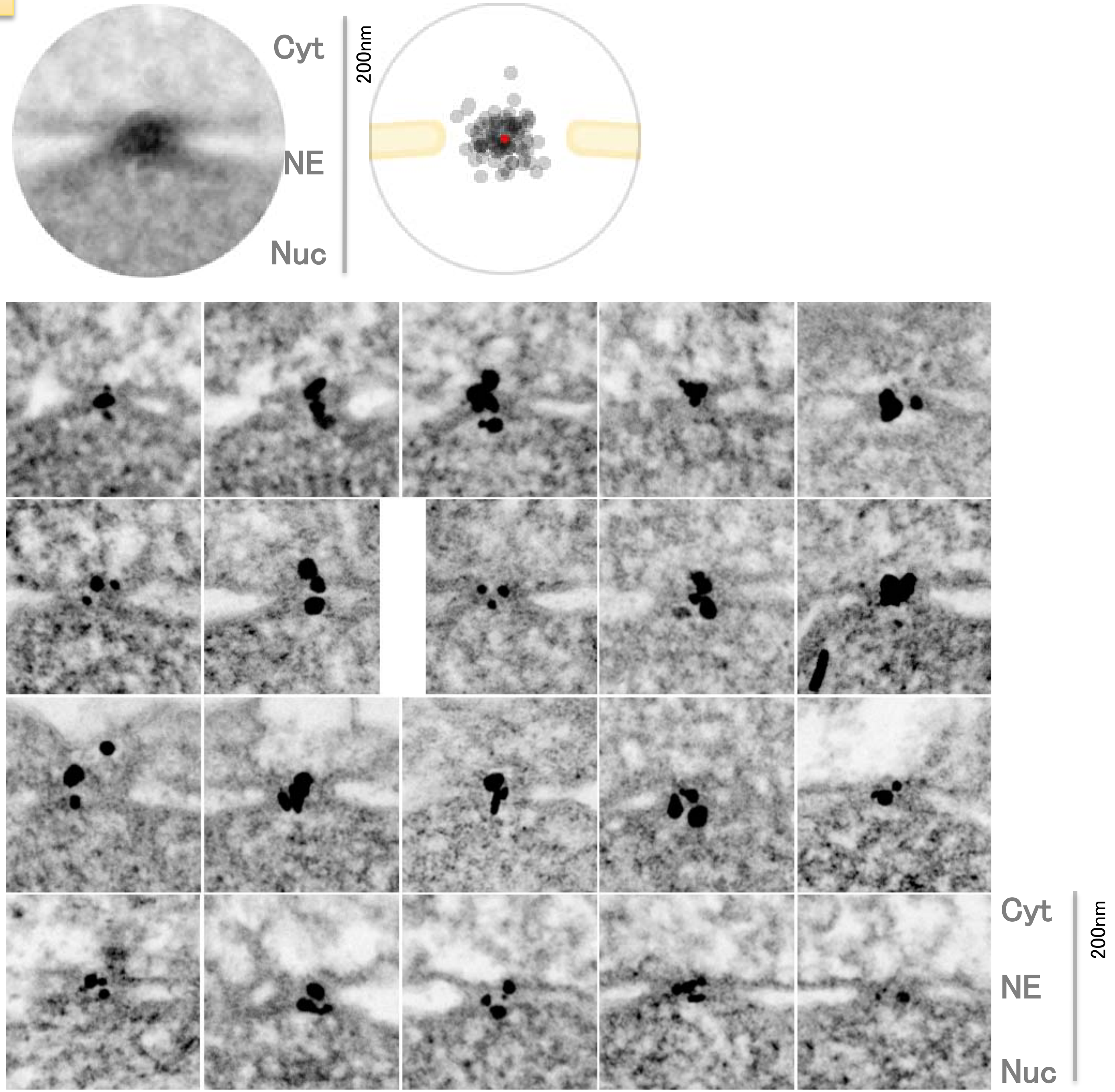

Nup45-GFP

projection

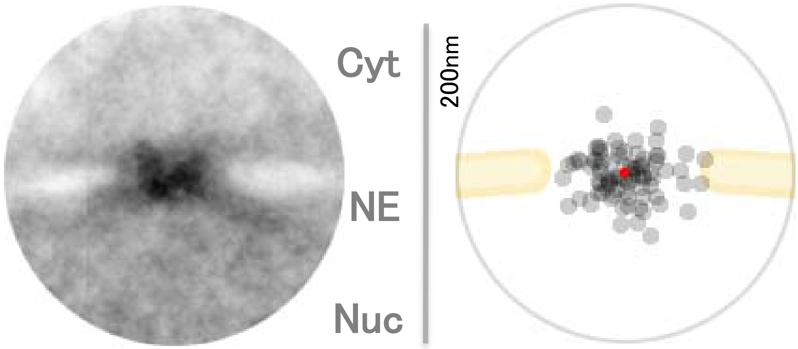

20 NPCs

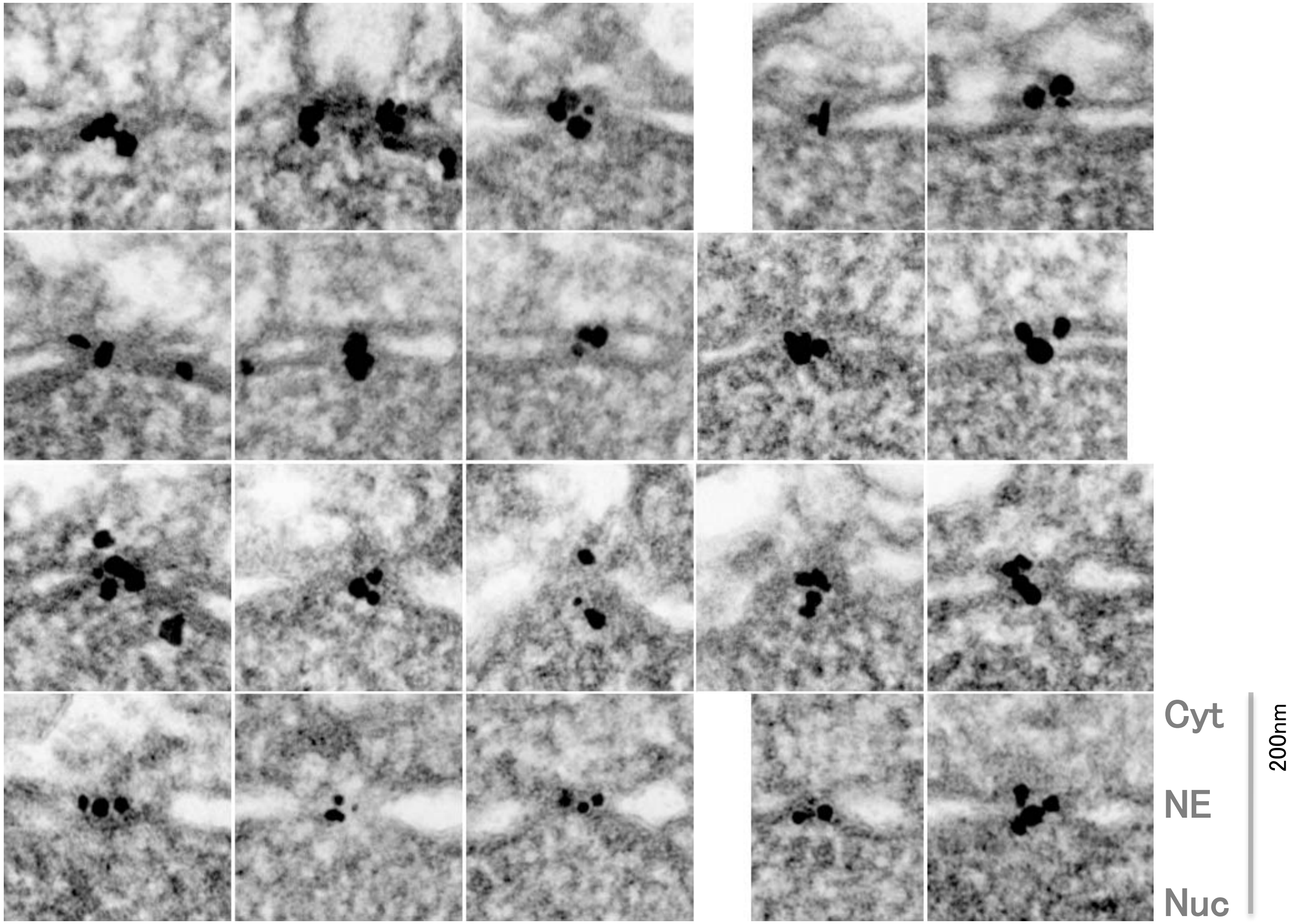

Nup98-GFP

projection

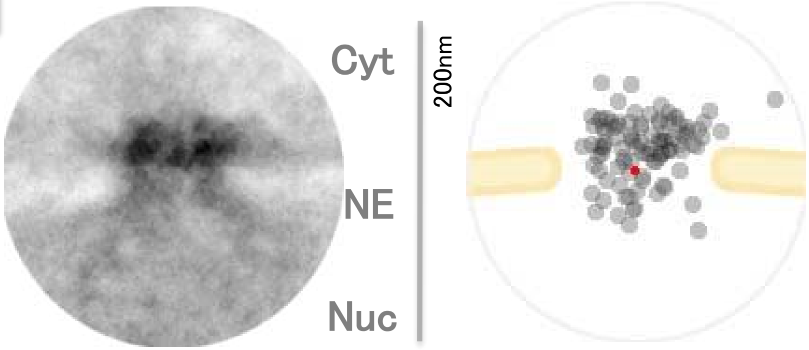

20 NPCs

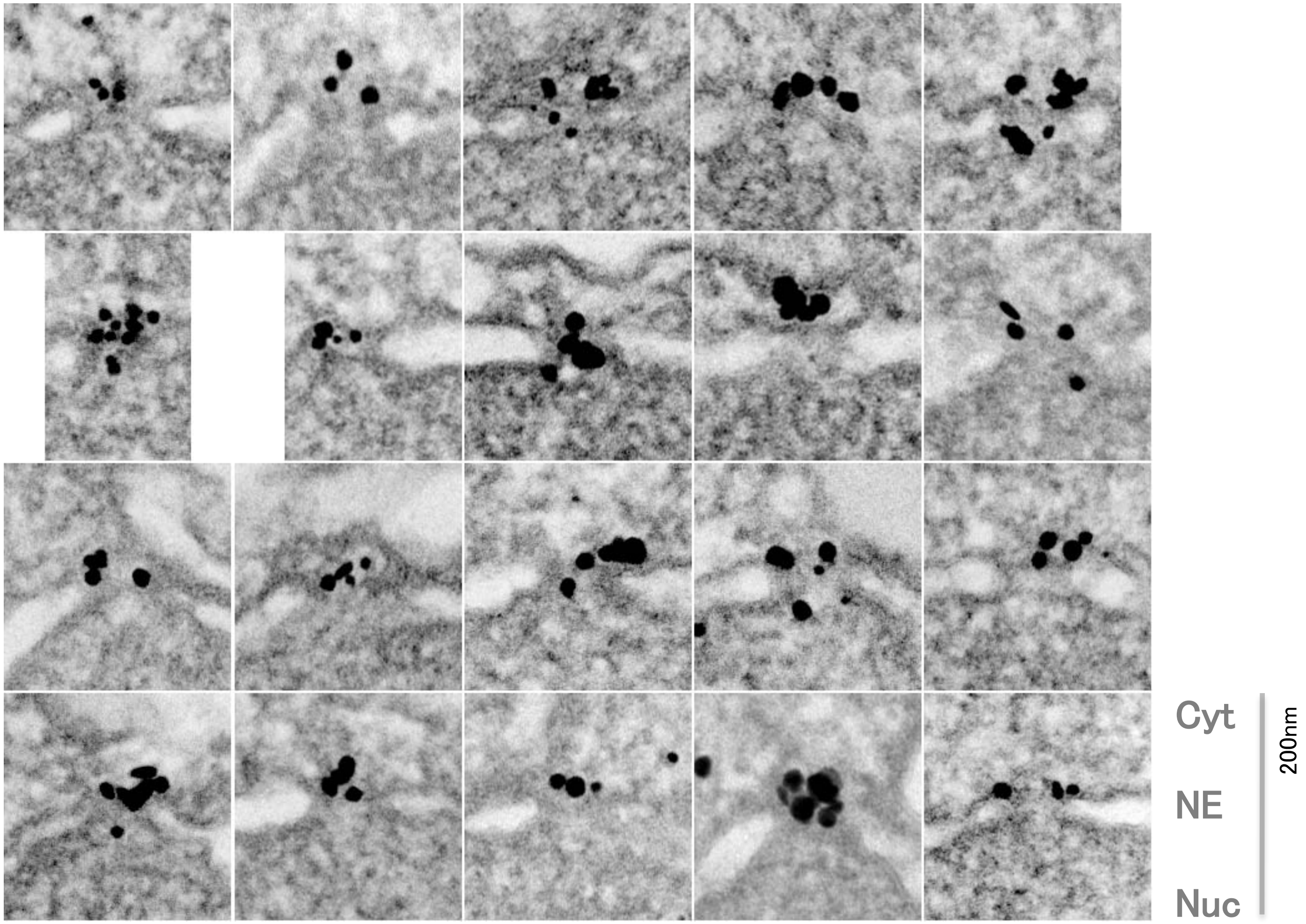

Nup98
(anti-Nup98)

projection

20 NPCs

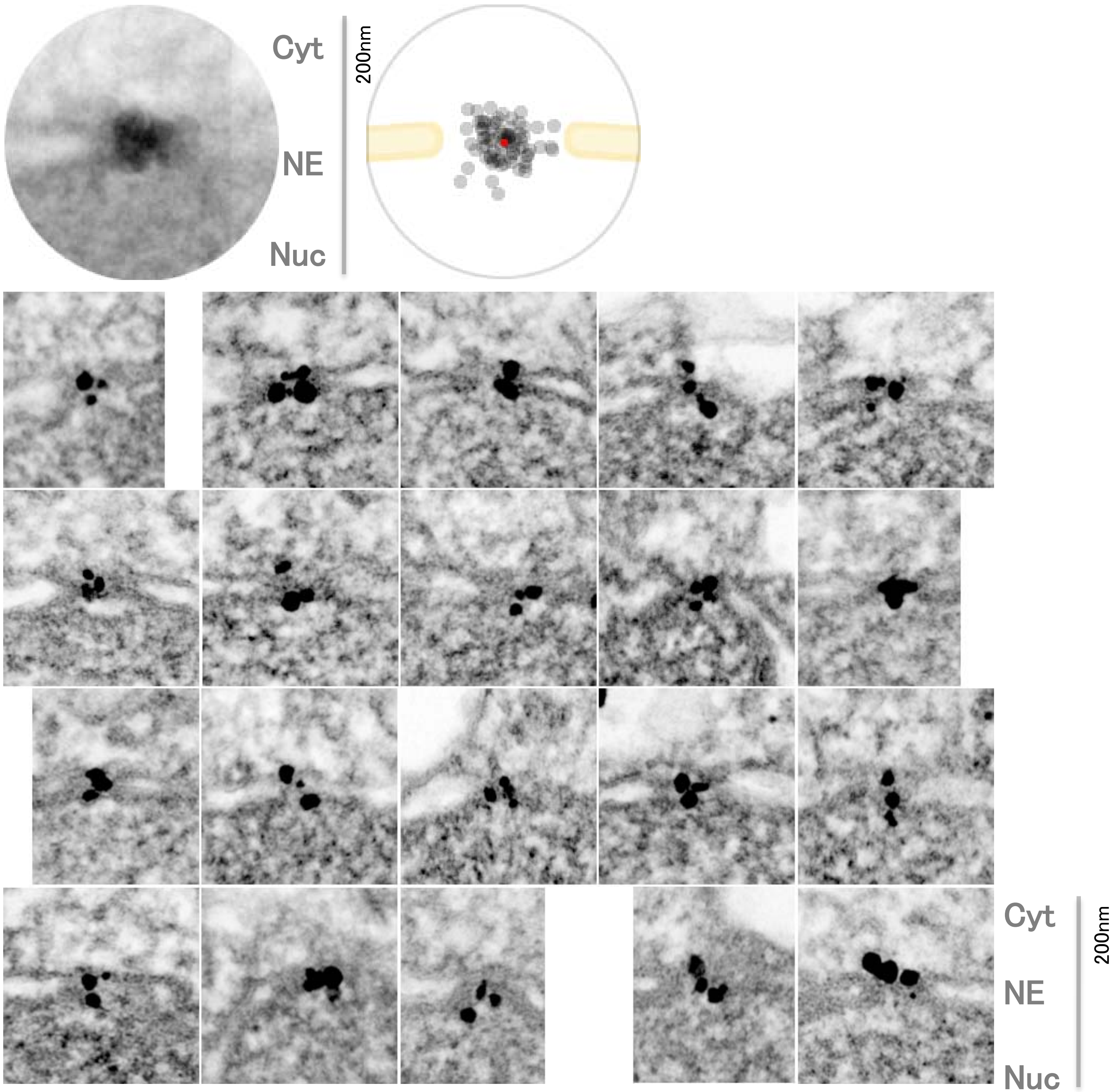

GFP–Nsp1

projection

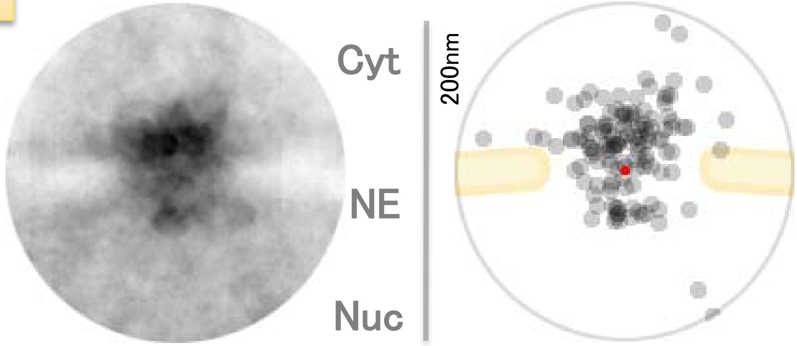

20 NPCs

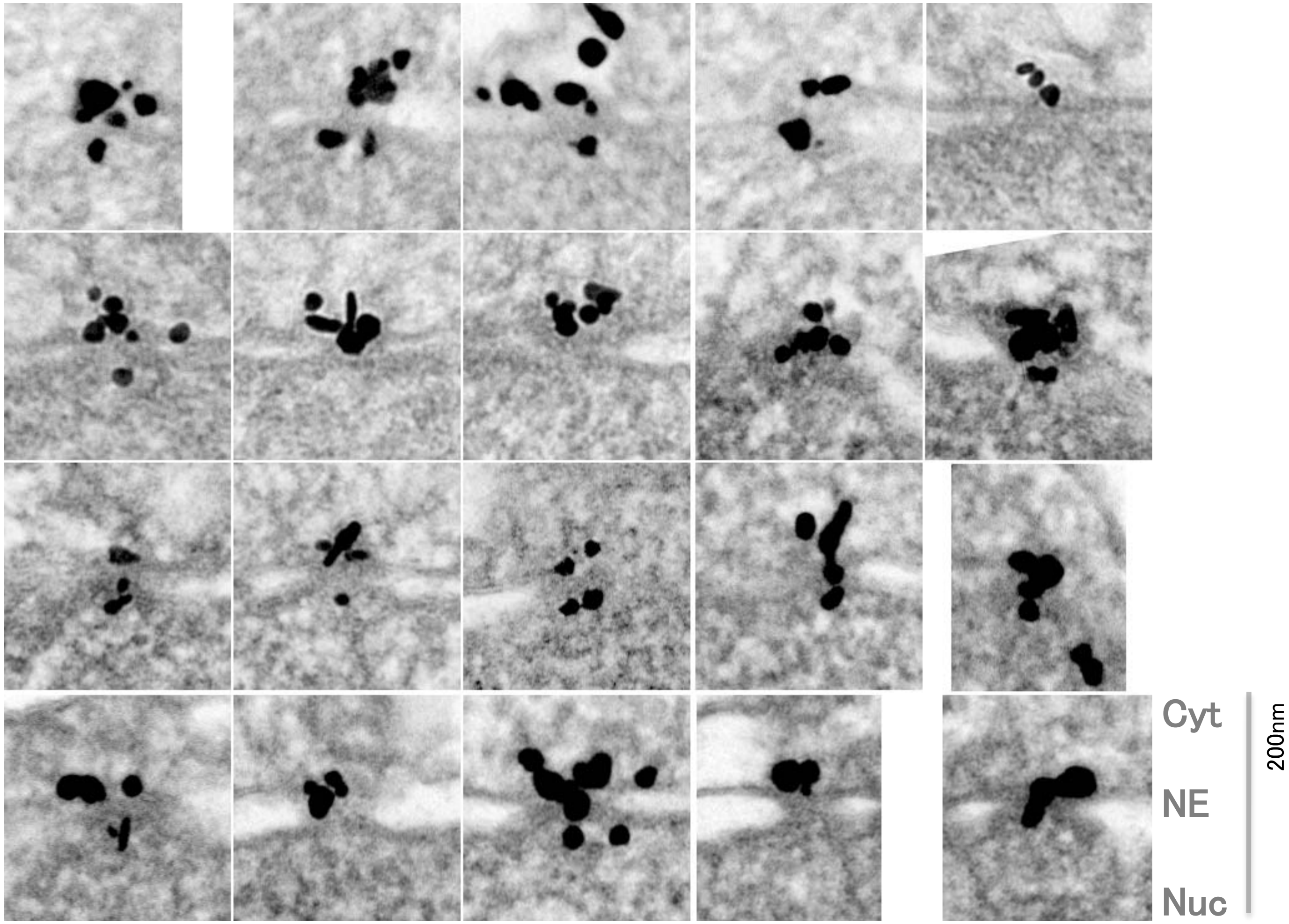

Cytoplasmic ring Nups

Nup82, Nup146

Nup82-GFP

projection

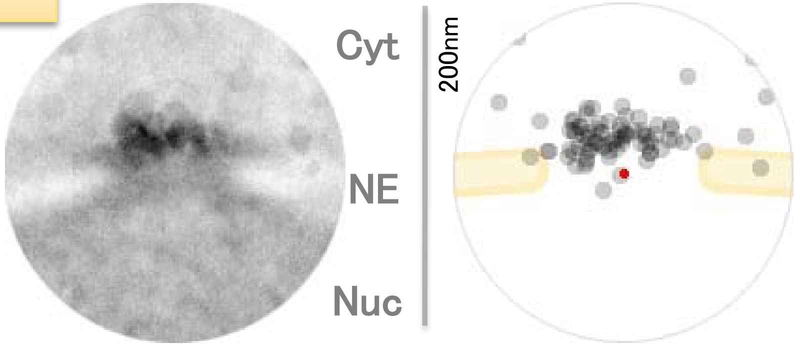

20 NPCs

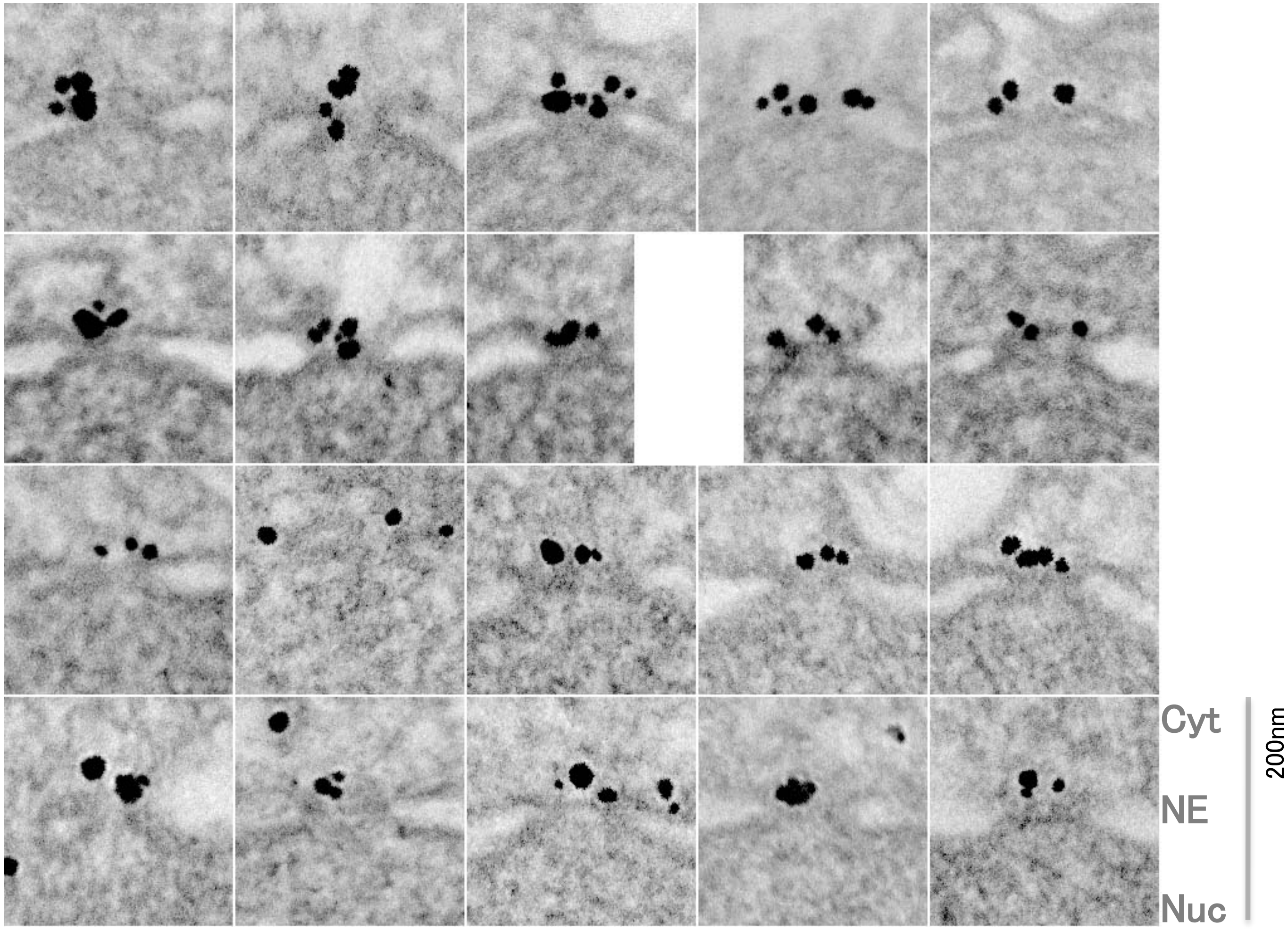

Nup146-GFP

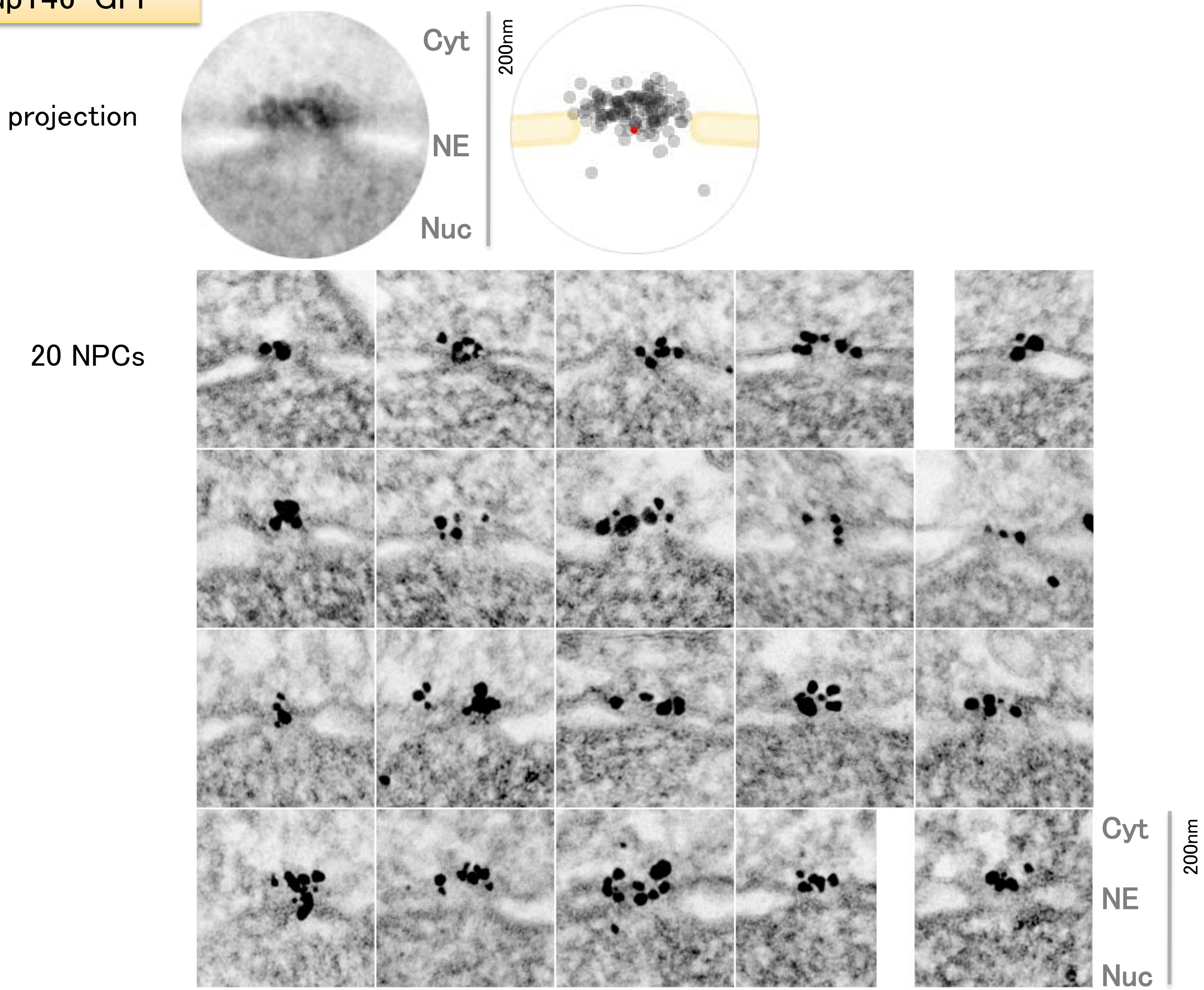

Amo1-GFP

projection

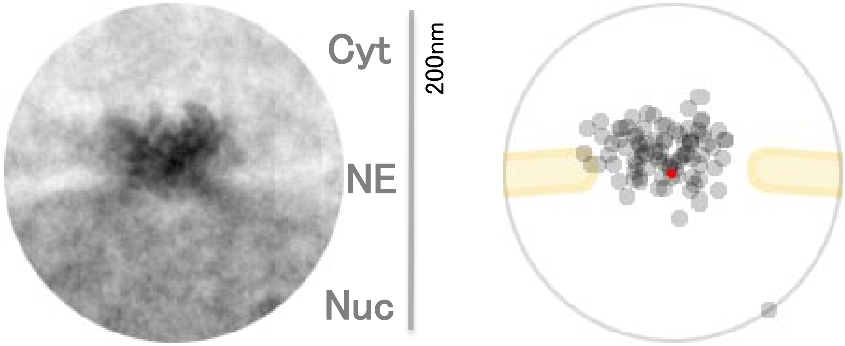

20 NPC

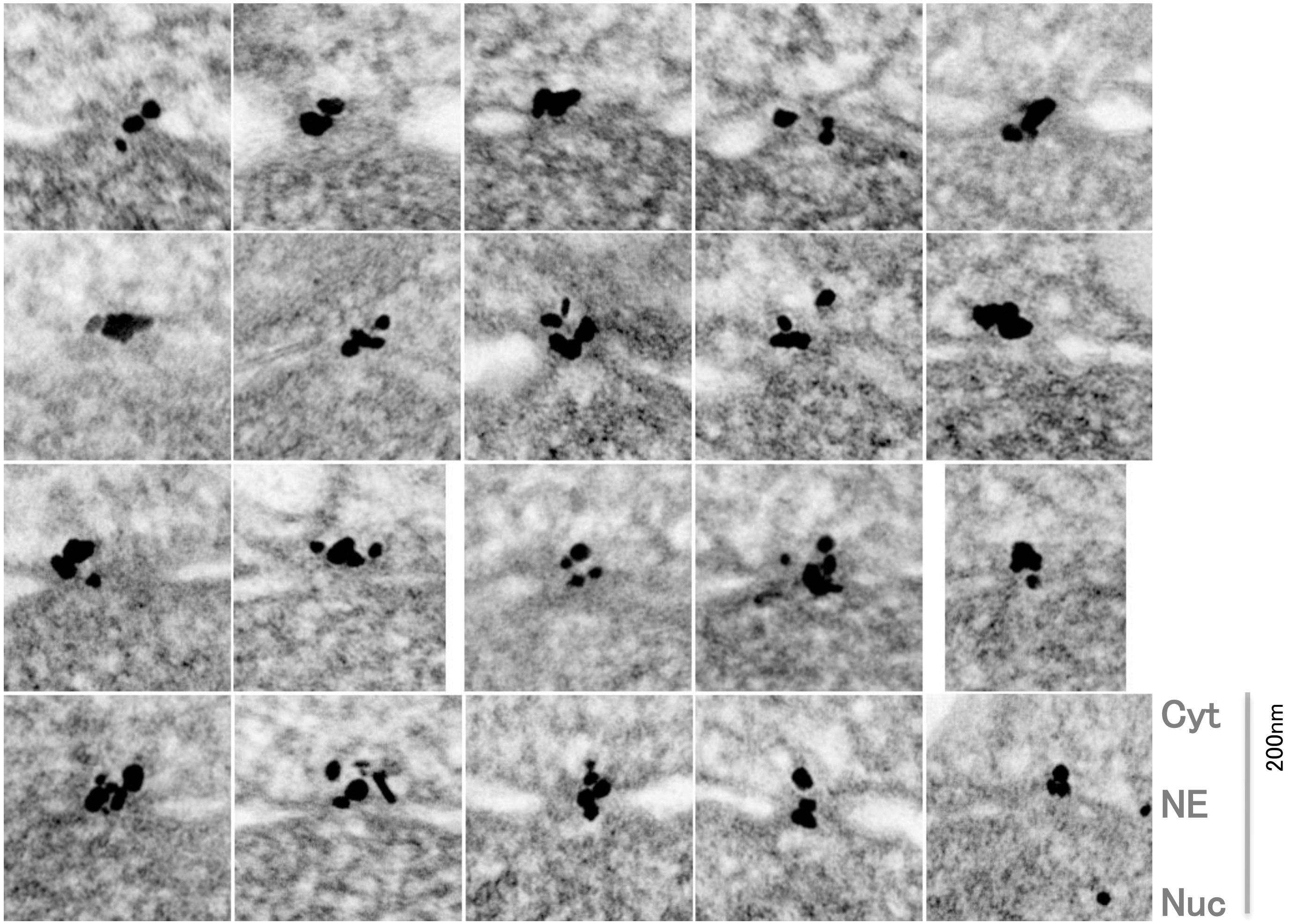

Transmembrane Nups

Cut11, Pom152, Pom34

Cut11-GFP

projection

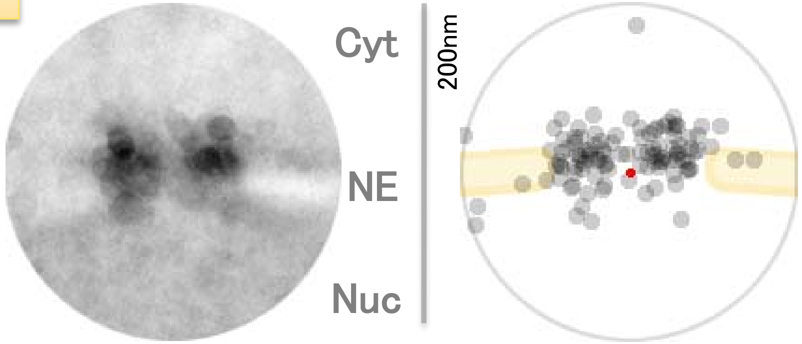

20 NPCs

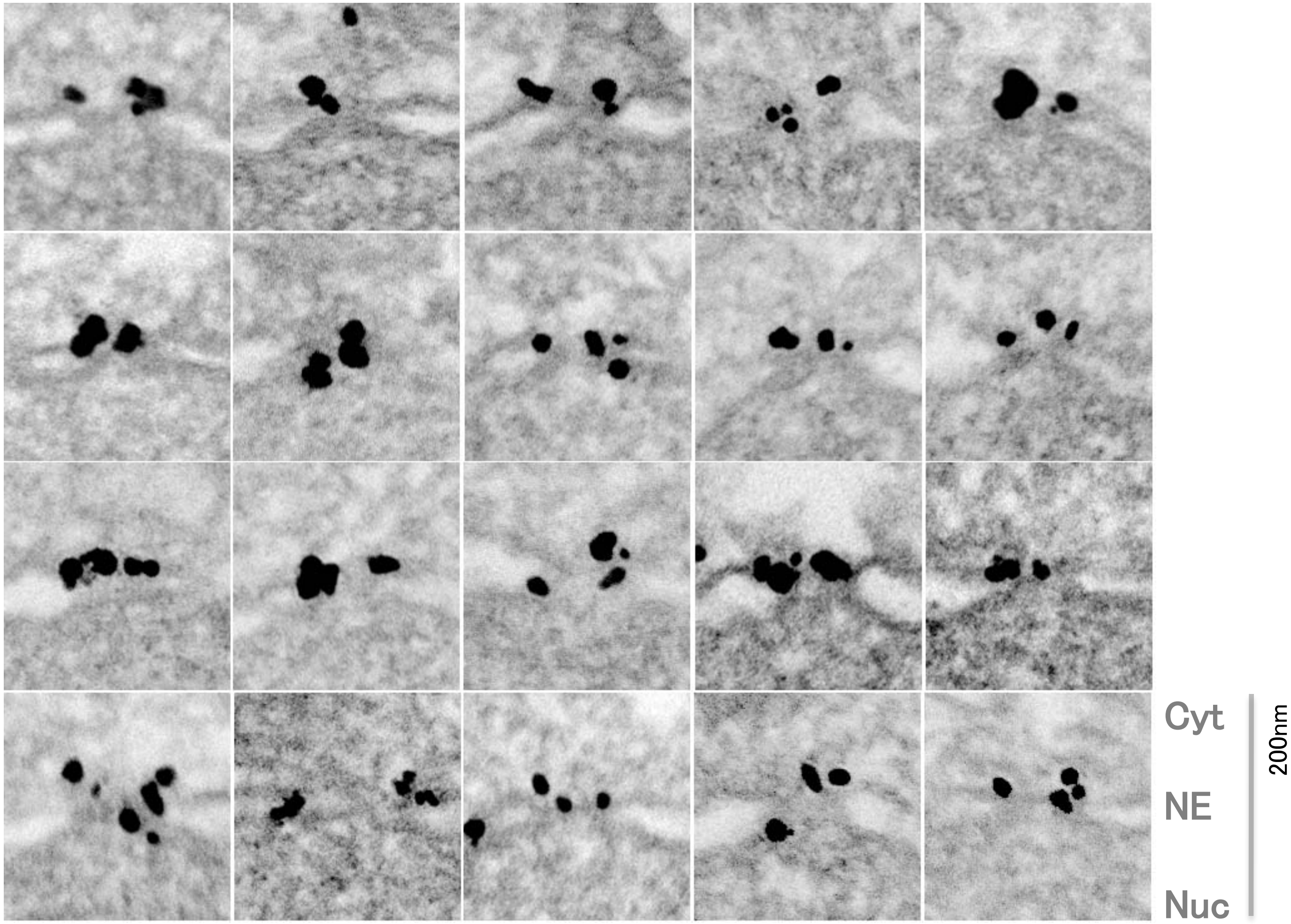

Pom152-GFP

projection

20 NPCs

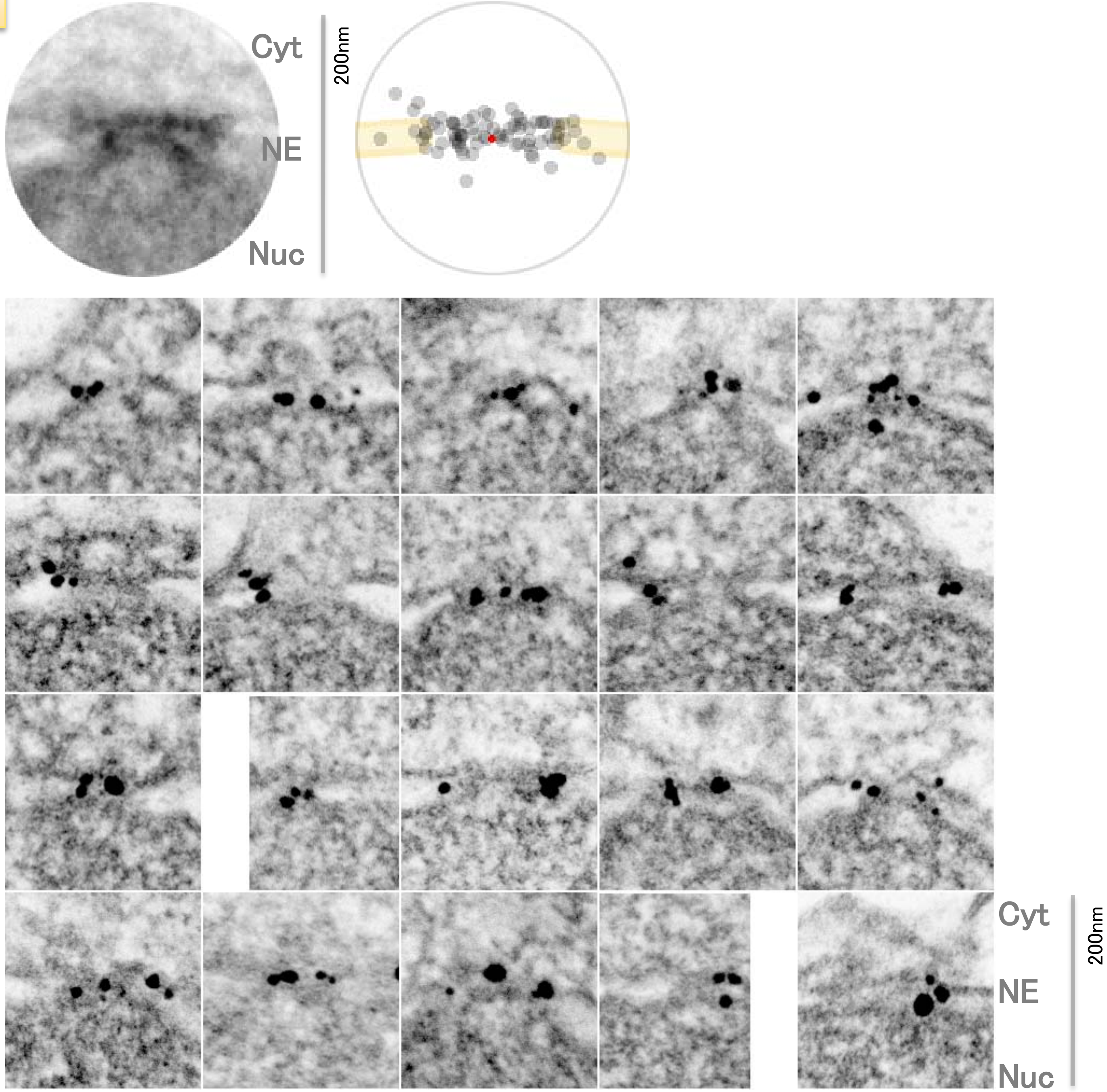

Pom34/Mug31-GFP

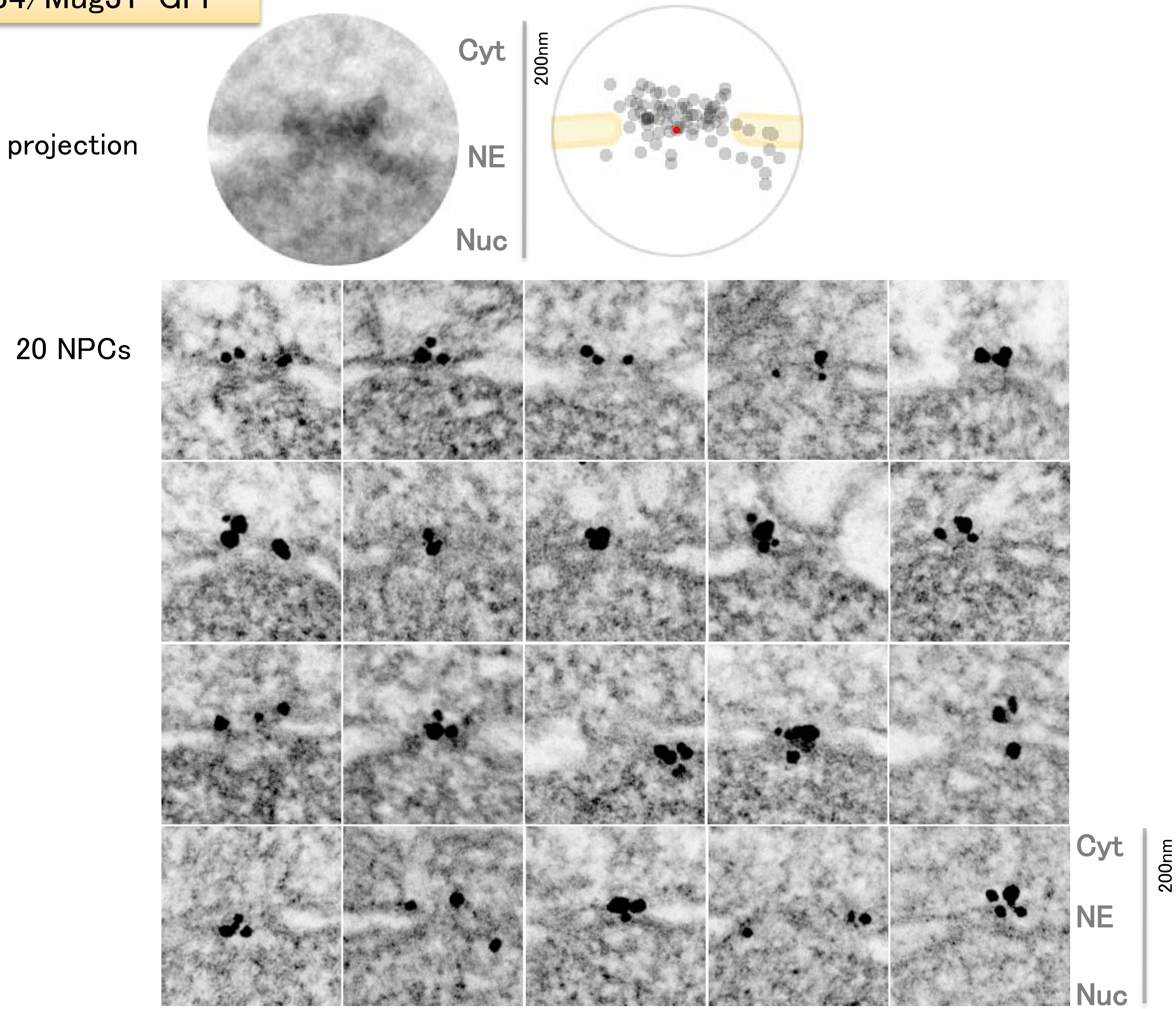

Nuclear basket Nups

Nup60, Nup61, Nup124, Alm1

Nup60-GFP

projection

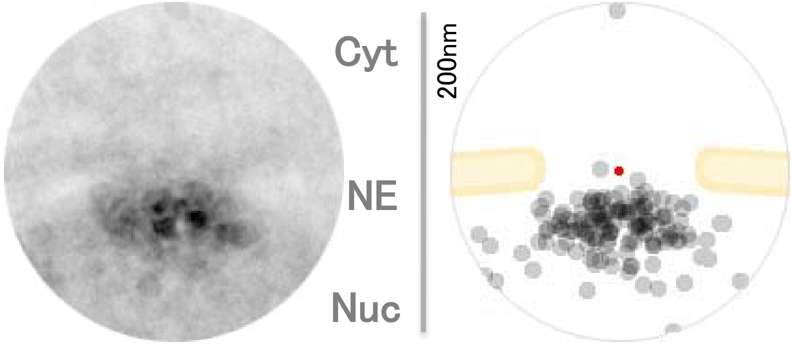

20 NPCs

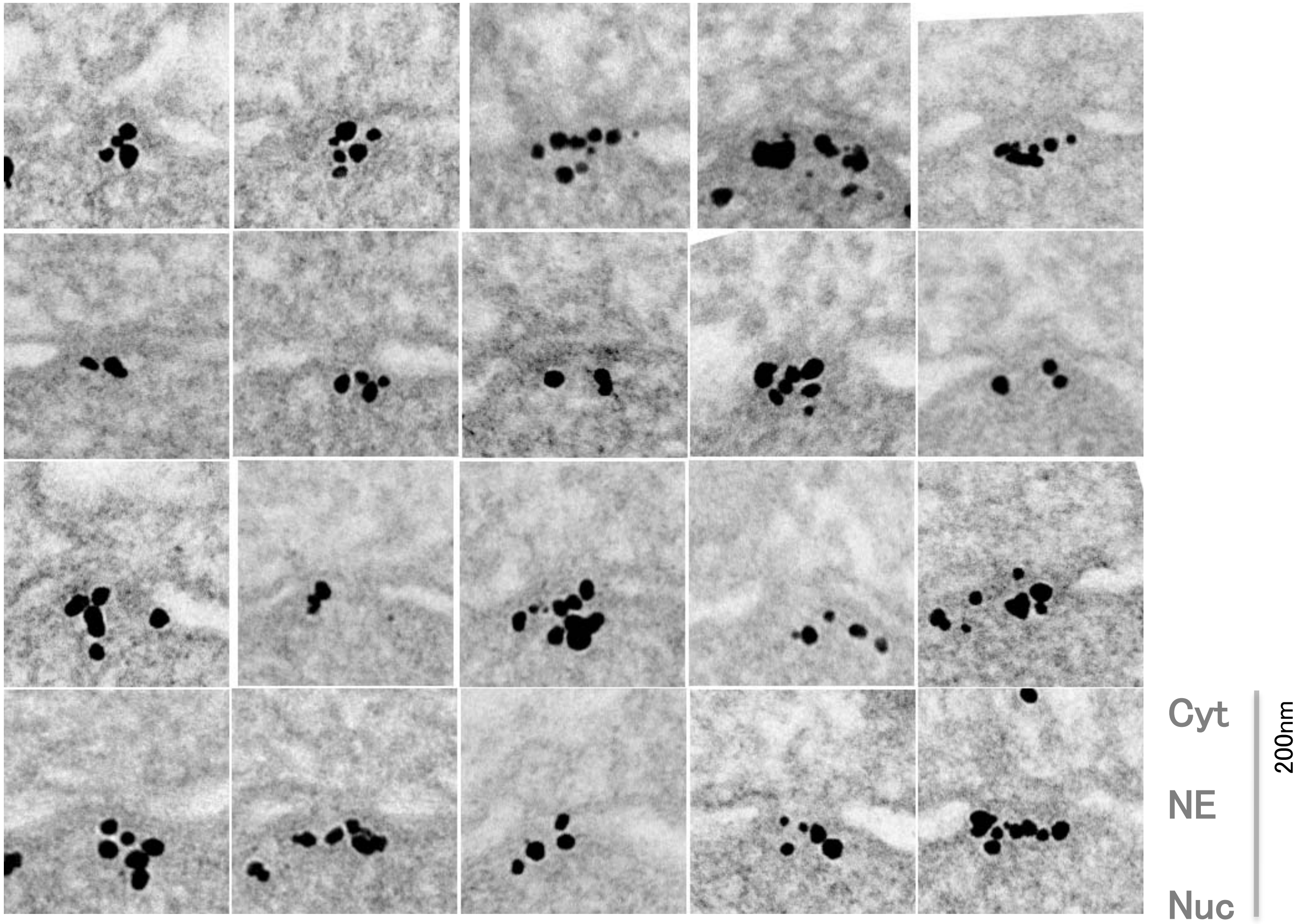

GFP-Nup61

projection

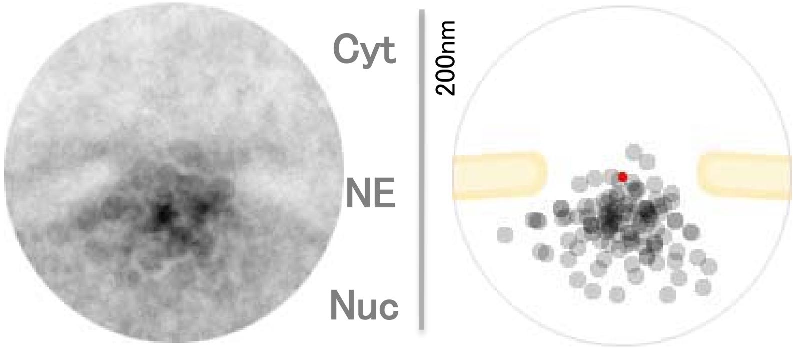

20 NPCs

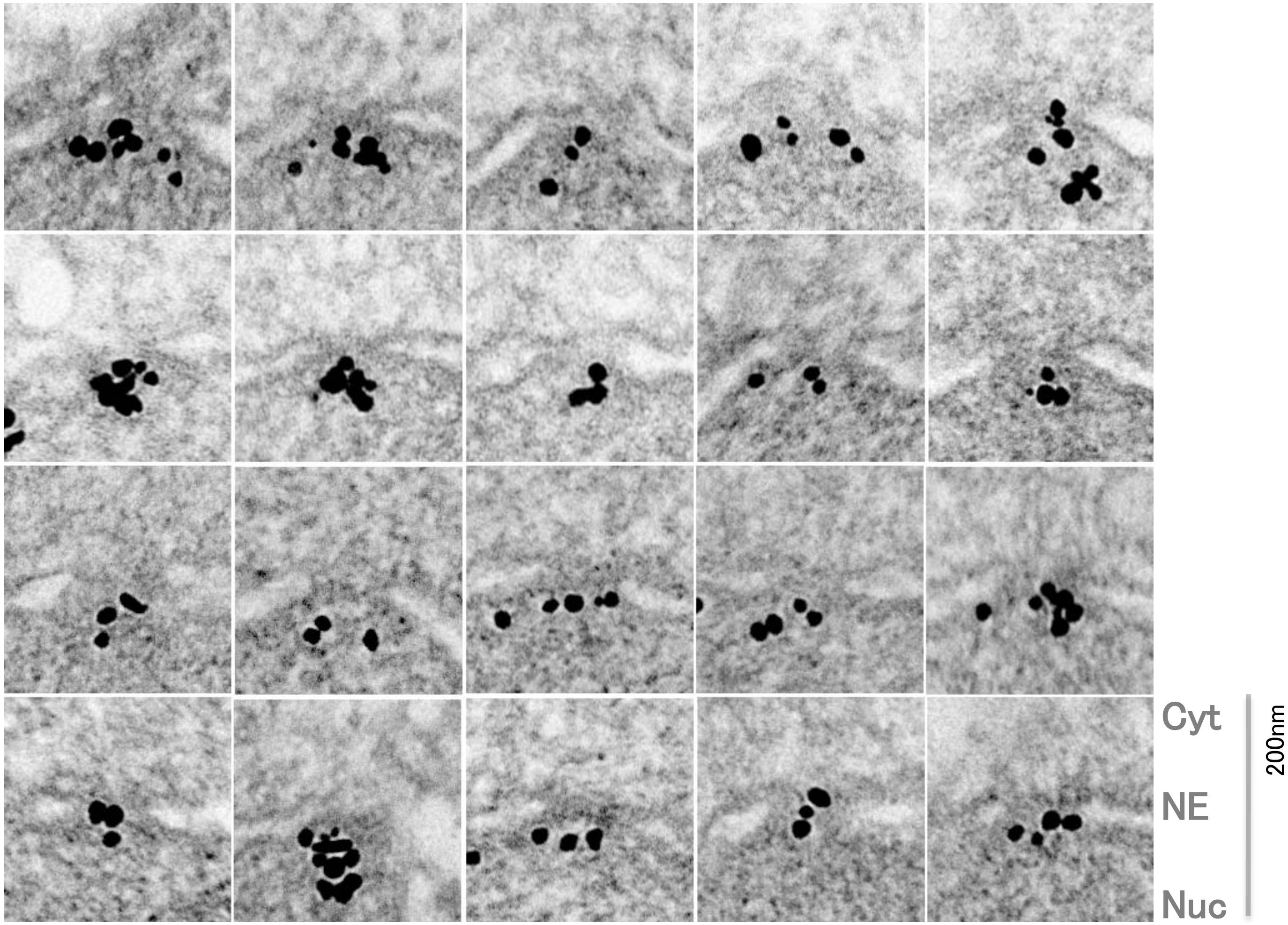

GFP-Nup124

projection

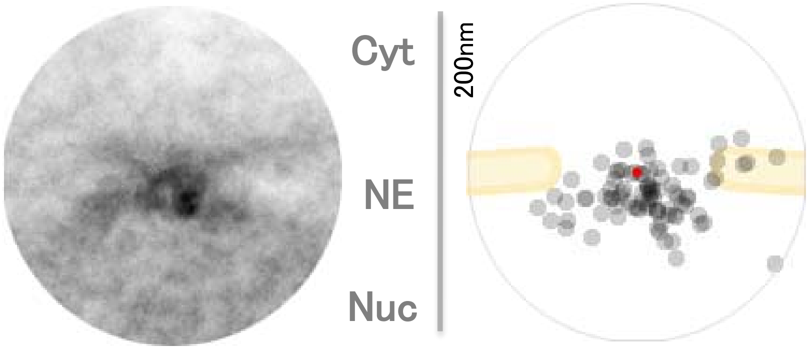

20 NPCs

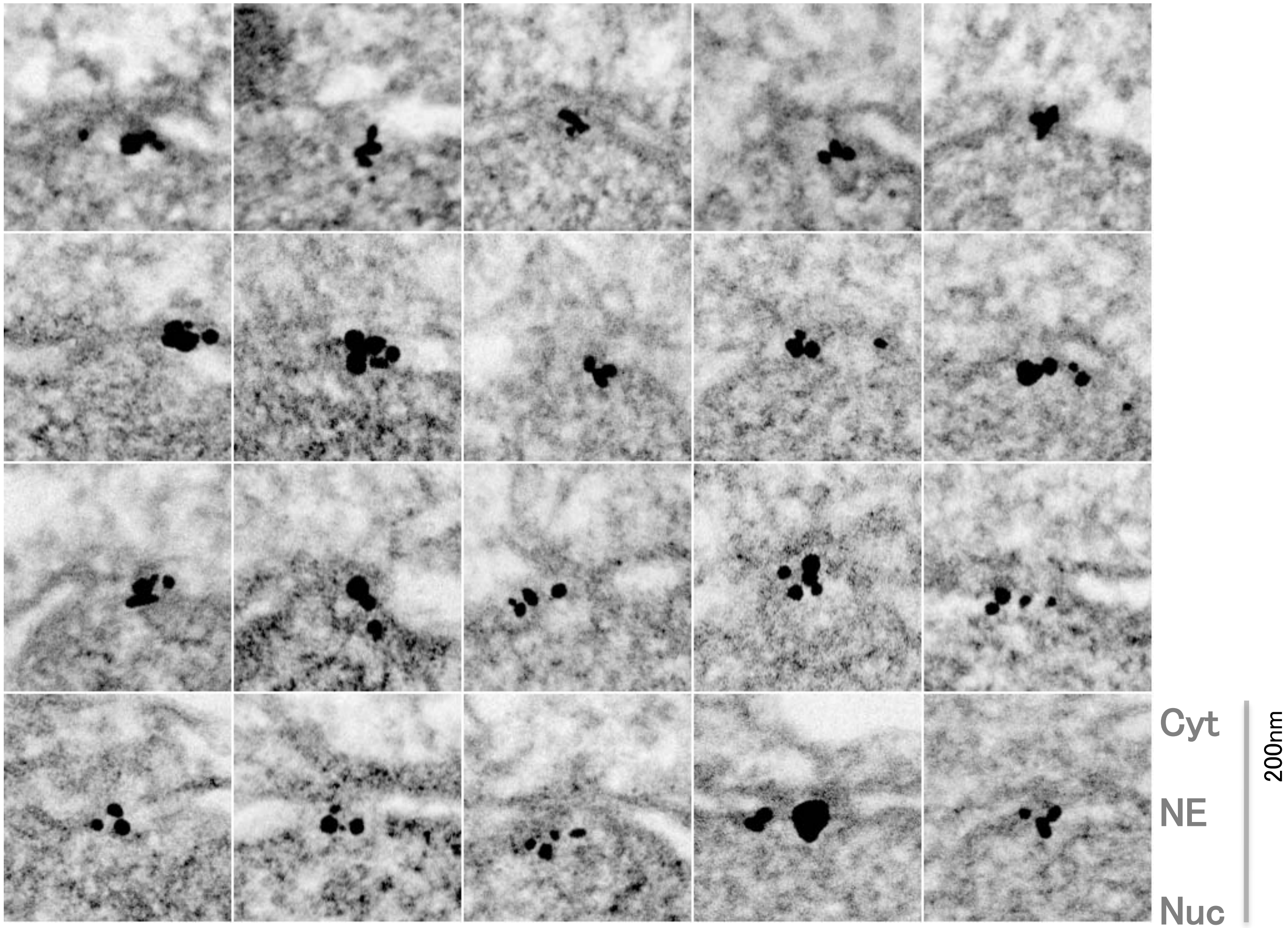

Alm1-GFP

projection

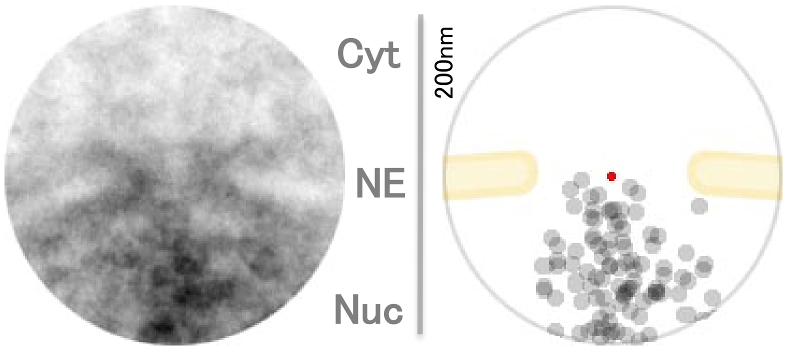

20 NPCs

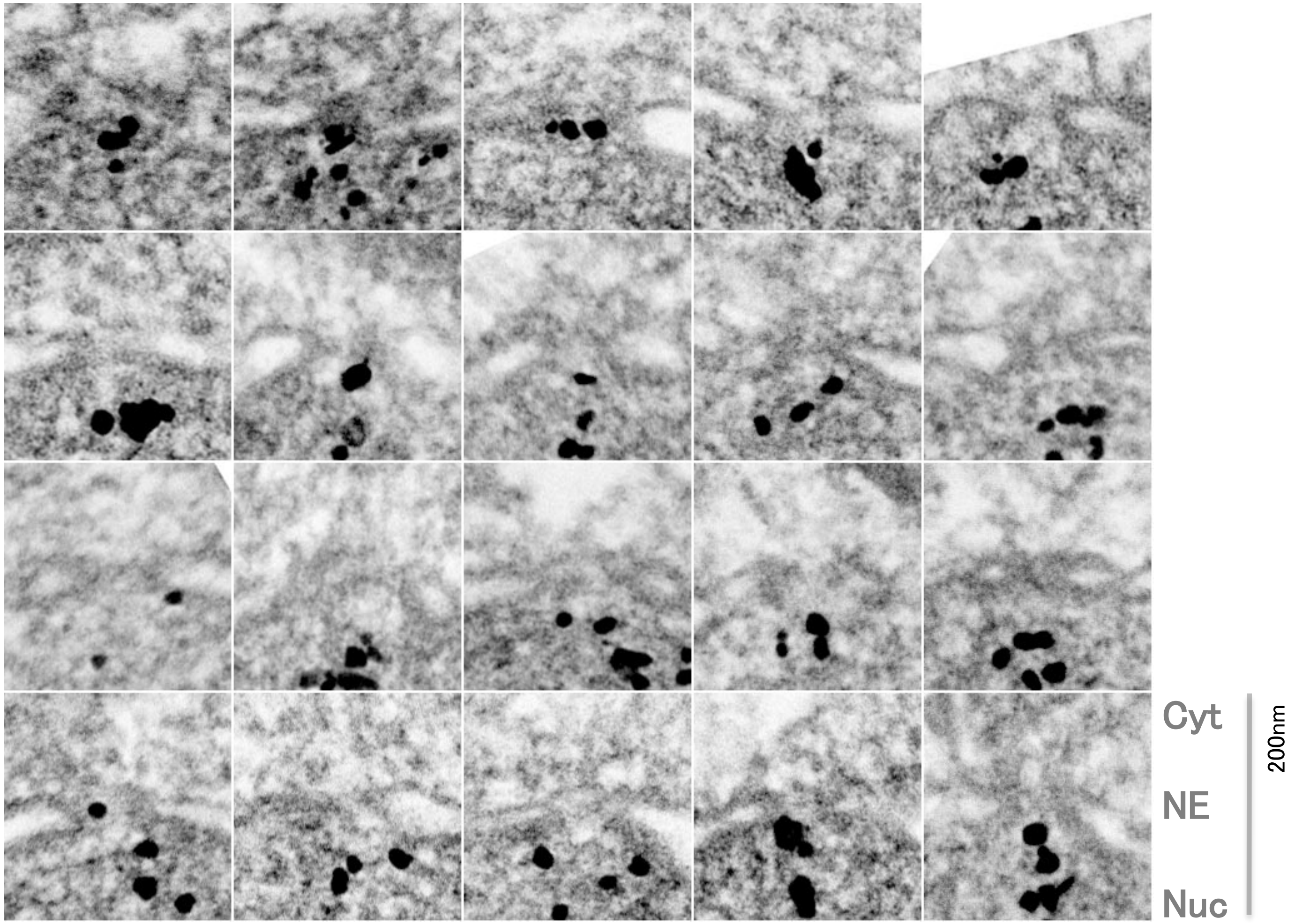

Supplement: S11 Dataset — (PDF) [file pgen.1008061.s022.pdf]
